# Unraveling the Chemistry of *meso*-Cl Tricarbocyanine Dyes in Conjugation Reactions for the Creation of Peptide Bonds

**DOI:** 10.1021/acsbiomedchemau.2c00053

**Published:** 2022-11-08

**Authors:** Rüdiger M. Exner, Fernando Cortezon-Tamarit, Haobo Ge, Charareh Pourzand, Sofia I. Pascu

**Affiliations:** † Department of Chemistry, University of Bath, Claverton Down Road, BA2 7AY Bath, U.K.; ‡ Department of Pharmacy and Pharmacology, University of Bath, Claverton Down Road, BA2 7AY Bath, U.K.; § Centre of Therapeutic Innovations, University of Bath, Claverton Down Road, BA2 7AY Bath, U.K.

**Keywords:** NIR fluorescence, meso-Cl cyanine dyes, keto-polymethines, confocal fluorescence imaging, EuK, [7,13]bombesin, d-glucosamine conjugation

## Abstract

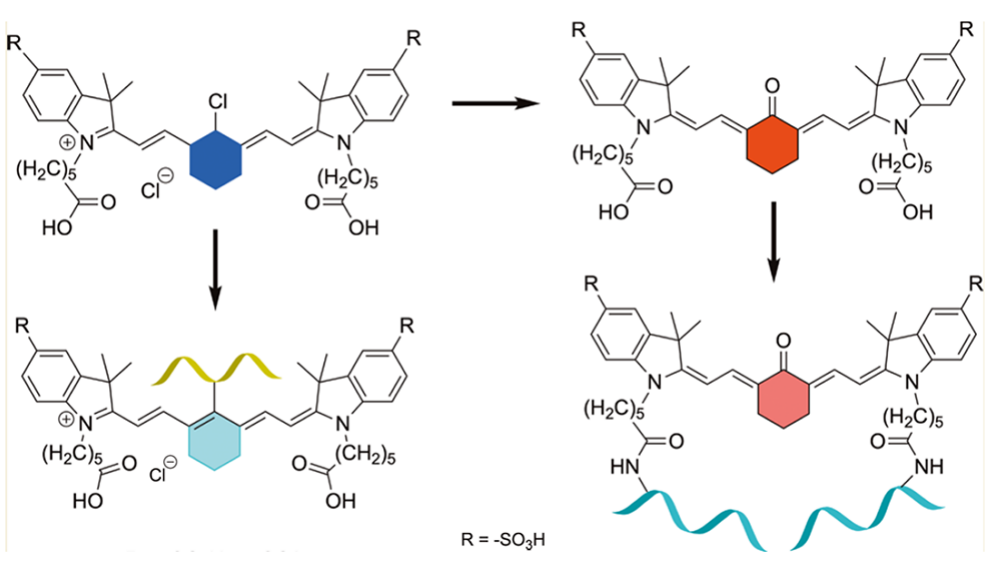

Tricarbocyanine dyes have become popular tools in life sciences and medicine. Their near-infrared (NIR) fluorescence makes them ideal agents for imaging of thick specimens or *in vivo* imaging, *e.g.*, in fluorescence-guided surgery. Among other types of cyanine dyes, *meso*-Cl tricarbocyanine dyes have received a surge of interest, as it emerged that their high reactivity makes them inherently tumor-targeting. As such, significant research efforts have focused on conjugating these to functional moieties. However, the syntheses generally suffer from low yields. Hereby, we report on the reaction of *meso*-Cl dyes with a small selection of coupling reagents to give the corresponding keto-polymethines, potentially explaining low yields and the prevalence of monofunctionalized cyanine conjugates in the current state of the art of functional near-infrared dyes. We present the synthesis and isolation of the first keto-polymethine-based conjugate and present preliminary investigation in the prostate cancer cell lines PC3 and DU145 by confocal microscopy and discuss changes to optical properties in biological media.

The development of functional near-infrared (NIR)-emitting fluorescent probes for image-guided surgery as well as multimodal imaging and personalized medicine is of great interest for biomedical applications and life sciences research. Some of the essential design requirements for these imaging probes for *in vivo* applications have recently been reviewed.^1−3^ In the NIR region of the electromagnetic spectrum, scattering coefficients, absorption, and autofluorescence of biological tissues reach local minima, resulting in overall low attenuation and allowing for larger penetration depths of the emitted light.^4−6^ Most prominent commercially accessible dyes for imaging in this region are those based on pentamethine and heptamethine cyanine frameworks, as well as the closely related tricarbocyanine dyes.^1,2,7−10^ The latter offer an additional handle for functionalization if the *meso*-position features a halide.^8,11^ Such dyes are highly reactive, with substitution reactions in the *meso*-position believed to proceed *via* an S_RN_1-type mechanism.^12^ In this context, derivatization of this Cy7 site can drastically alter the photophysical properties,^11^ due to substantial changes of the charge distribution along the polymethine backbone.^13^ In addition, this high reactivity gives the *meso*-Cl dyes inherent cancer-targeting properties, as reactions with thio nucleophiles, like cysteine sidechains in proteins and peptides, occur under physiological conditions.^14,15^ It has been reported that the reaction of *meso*-substituted NIR dyes with human serum albumin (HSA) may be responsible for the tumor-targeting properties observed *in vivo*. Such bioconjugates would readily form *in vivo.*(16,17) In this context, it has been of interest to investigate the role of subsets of endothelial cells, such as the recently described nanoparticle-transporting endothelial cells (NTECs).^18^ Additionally, m*eso*-substitution reactions have been applied to prepare targeted NIR-emitting probes,^8,19−21^ improve photophysical properties, or introduce functionalities for the design of activatable fluorescent probes.^22,23^ Mellanby et al. demonstrated the potential of *meso*-*N*-triazole-functionalized dyes for long-term imaging,^24^ and Wang et al. demonstrated the emergence of targeted, multimodal imaging probes using the *N*-triazole scaffold.^25^

We aimed to synthesize bioconjugates of *meso*-Cl dyes with a selection of standard peptide coupling agents and to incorporate tumor-homing targeting groups (Scheme 1). We focus hereby on addressing the synthetic challenges which were raised by the laboratory-scale synthesis and functionalization of dye **1**, a sulfonated analogue of the popular **MHI-148** (Figure 1), and the isolation and characterization of new bioconjugates derived from this Cy7 framework. Synthetic avenues targeted at a scalable peptide coupling chemistry were explored hereby with an aim to access a new toolkit for the assembly of modular probes for fluorescence imaging. Taken together, these observations prompted our curiosity-driven investigation aiming to describe the reasons behind the low conjugation yields at the peptide bond formation and analytical purification under typical reverse-phase chromatography conditions (including the use traces of trifluoroacetic acid (TFA) in the reverse-phase high-performance liquid chromatography (HPLC)) as well as the prevalence of monofunctionalized cyanine conjugates in the current state of the art of functional NIR dyes. Literature accounts of amide bond formation from *meso*-Cl dyes report low yields, and mostly monofunctionalization of dyes.^32^ Laboratory-scale syntheses of water-soluble dyes were complicated by difficult separations in aqueous environment and low yields which appeared to be caused by side reactions and decompositions during purification and HPLC analysis. To obtain the *meso*-Cl tricarbocyanine **1**, an early synthetic procedure (reported in 2004) was followed.^28^ However, in our hands, material of sufficient purity was not forthcoming following this protocol. As such, we developed a modified synthetic purification procedure (reliant on the selective precipitation of **1** from dilute hydrochloric acid, see the Supporting Information) which enabled the facile separation from starting materials and by-products. The optical properties of compound **1** were subsequently determined (see the Experimental Section and the Supporting Information): overall, all spectroscopic measurements are in agreement with recent literature of similar dyes in aqueous systems and broadly comparable to the recently developed **QuatCy** and the established **MHI-148** dye (Figure 1).^29^*In vitro* investigations of **1** in living cells (specifically, in the common PC3 cell line, derived from the bone metastasis of a stage 4 prostate cancer patient) indicated no significant inhibition of cell metabolism or growth by the compound used in concentrations of up to 250 μM, similar to other results highlighting the low cytotoxicity of sulfonated cyanines.^30,31^

**Figure 1:**
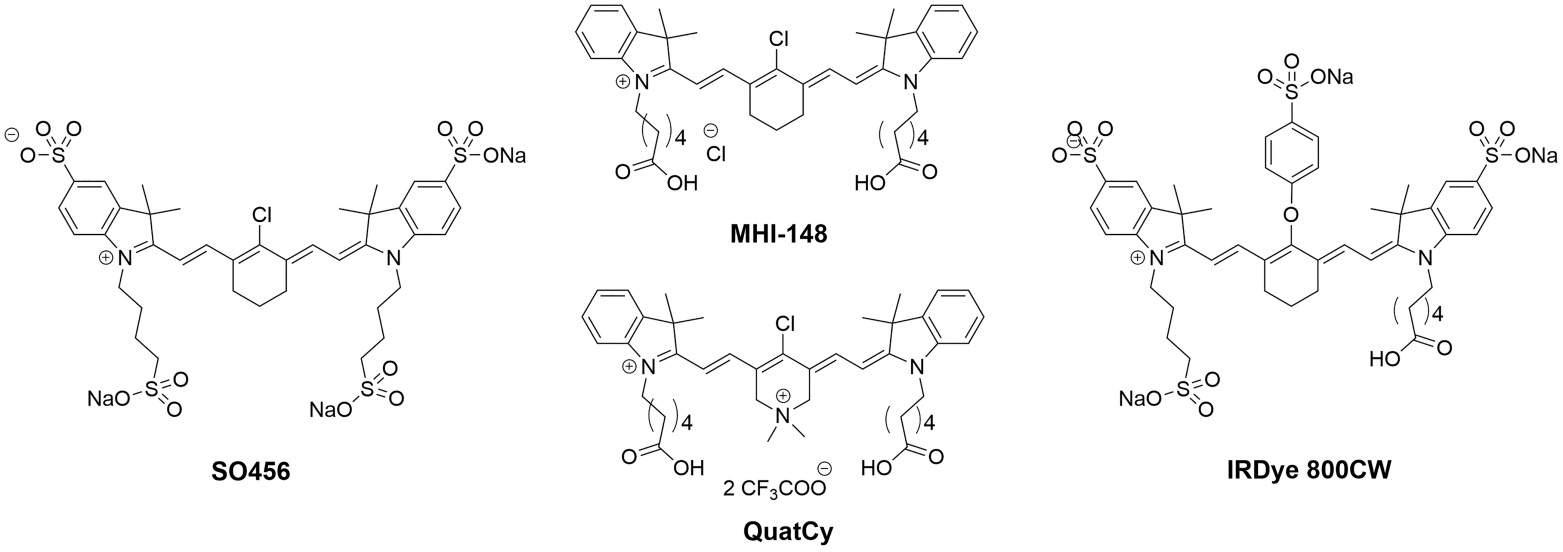
Overview of commonly surveyed *meso*-substituted tricarbocyanine dyes. Derivatives of the commercial **SO456** and **IRDye 800CW** dyes are currently used in clinical trials.^20,26,27^

**Scheme 1:**
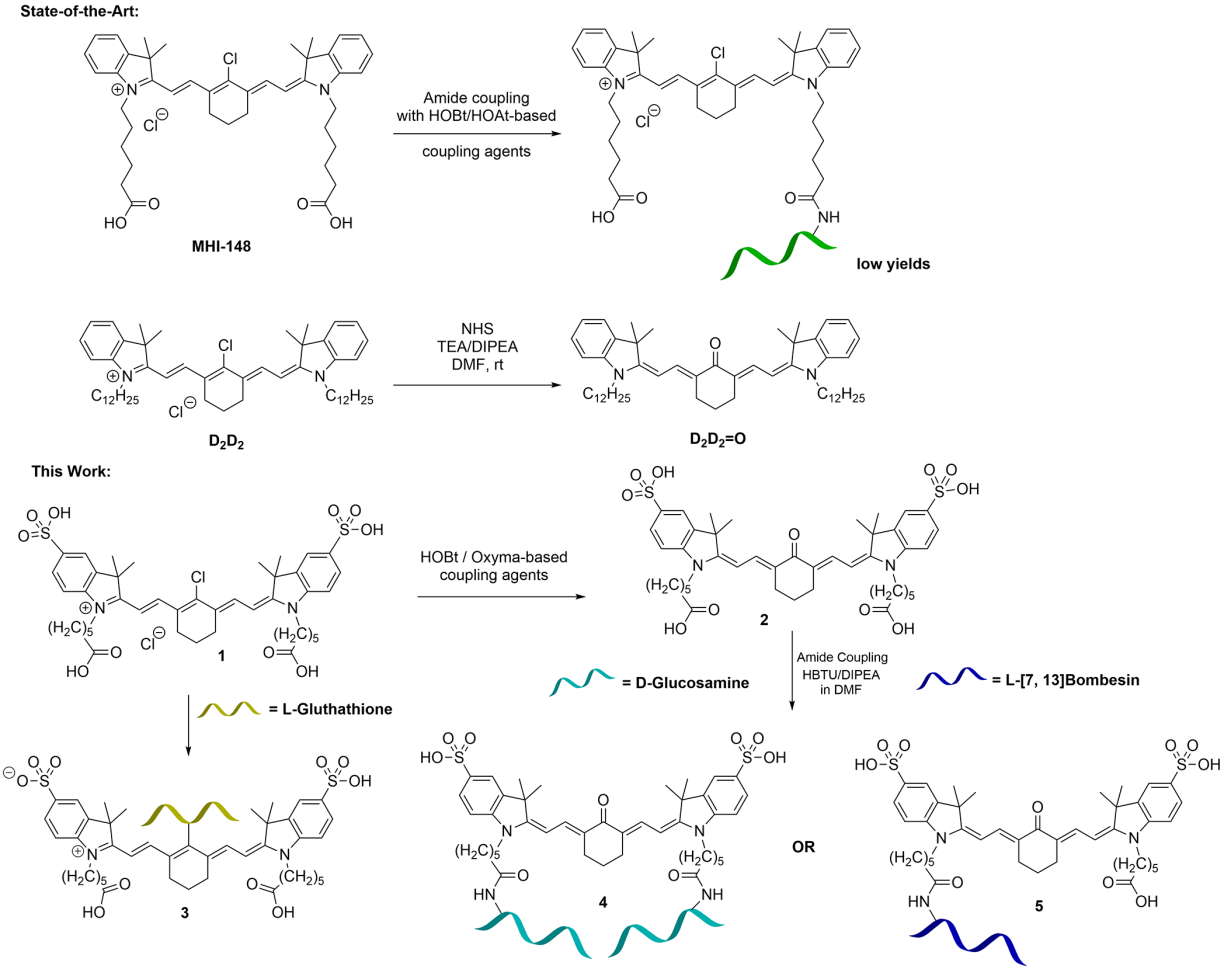
Overview of This Work and Related Coupling Reactions in the State of the Art Colored helix is a general representation of functionalized substrates featuring amine bonds; the original motivation was the incorporation of the EuK tag (or LysCOGlu) using standard peptide coupling agents.

We then aimed to synthesize a library of imaging agents incorporating the cyanine framework **1** as a highly modular building block for fluorescence imaging of various cancer cells, using coupling reactions of this *meso*-Cl dye with glucosamine, a simple [7,13]bombesin peptide fragment, or the urea-based derivative EuK (or LysCOGlu).

As mentioned above, literature accounts of *meso*-Cl tricarbocyanine dye conjugates obtained through amide bond formation with demanding reaction partners typically report low yields^32^ and the state of the art also predominantly features monofunctionalized derivatives (Scheme 1). The same is not true, however, for cyanine dyes that do not feature *meso*-Cl substituents, where coupling chemistry was reported to proceed more successfuly.^33,34^ The reasons for this feature or attempts at explaining the nature of the low yields have not been fully explored, and the outcomes of coupling chemistry involved therefore confined this otherwise promising synthetic building block′s functionalization chemistry to the realm of analytical, rather than synthetic scaled-up approaches. Hereby, upon attempting the standard peptide coupling conjugation reactions to deliver various bioconjugates, we noticed that the color of the solutions changed drastically during the reaction (see Figure 2 and the Supporting Information). For the case study of coupling one or two EuK tags (denoted LysCOGlu) to the −COOH groups of **1** using the reagent PyBOP, the reaction mixture was monitored by ultraviolet–visible (UV–vis) spectroscopy in the region of 350–900 nm. The absorption spectra were recorded (a) before and (b) after the peptide coupling reaction was carried out, and the corresponding spectra are depicted in Figure 2. This shows the absorption assignable to **1** and that of the resulting postcoupling crude reaction mixture. The intense band at *ca.* 560 nm suggested the formation of the corresponding keto-polymethine as a side product.^13,35^ Earlier work on pH sensors considered the conversion of its cyanine ketone derivatives to hydroxy cyanines upon protonation and established that the inherent visible light absorption of a simple keto-polymethine type **MHI-148-O** (with λ_max_ = 535 nm) shifts significantly to the NIR region (with λ_max_ = 709 nm) due to pH variations.^36^ Attempts to isolate the desired *meso*-Cl dye conjugate **1-EuK** were challenging and typically gave indications of the formation of the desired compound in very low yields (<10%) or gave only some evidence in spectroscopic measurements of the formation thereof. This is in line with the observations by other authors for similar approaches on peptides.^29,32^ The peptide coupling reactions endeavored hereby to obtain *meso*-Cl dye bioconjugates on a laboratory scale, which simultaneously retain the −Cl site and incorporate at least one conjugated peptide bond-linked functionality, only resulted in the isolation of traces of the desired conjugates on an analytical scale. Evidence for the formation of such *meso*-Cl bioconjugates was gathered by mass spectrometry (see the Supporting Information). It is well-known that the reaction of *meso*-Cl tricarbocyanine dyes with *N*-hydroxysuccinimide (NHS) gives rise to keto-polymethines.^36^ Analogous functionalization reactions upon treatment with other coupling reagents have not been described. Upon testing the stability of **1** in DMF, in the presence of *N*-hydroxysuccinimide (NHS), 1-hydroxybenzotriazole (HOBt), or *K*-Oxyma Pure, we determined that all three reagents lead to the formation of the keto-polymethine **2**, as shown in Figure 3. Apart from the main product **2**, other side products seem to be produced for coupling chemistry reactions of the type shown in Figure 2, and which have not been unambiguously identified despite extensive mass spectrometry investigations of the crude reaction mixtures (Supporting Information). The presence of side products was most apparent from reactions with HOBt, and reactions with both NHS and *K*-Oxyma Pure yielded unassignable side products. A possible reason may be that intermediates formed with HOBt may be more reactive or that water (from the addition of HOBt·H_2_O) may facilitate their formation.

**Figure 2:**
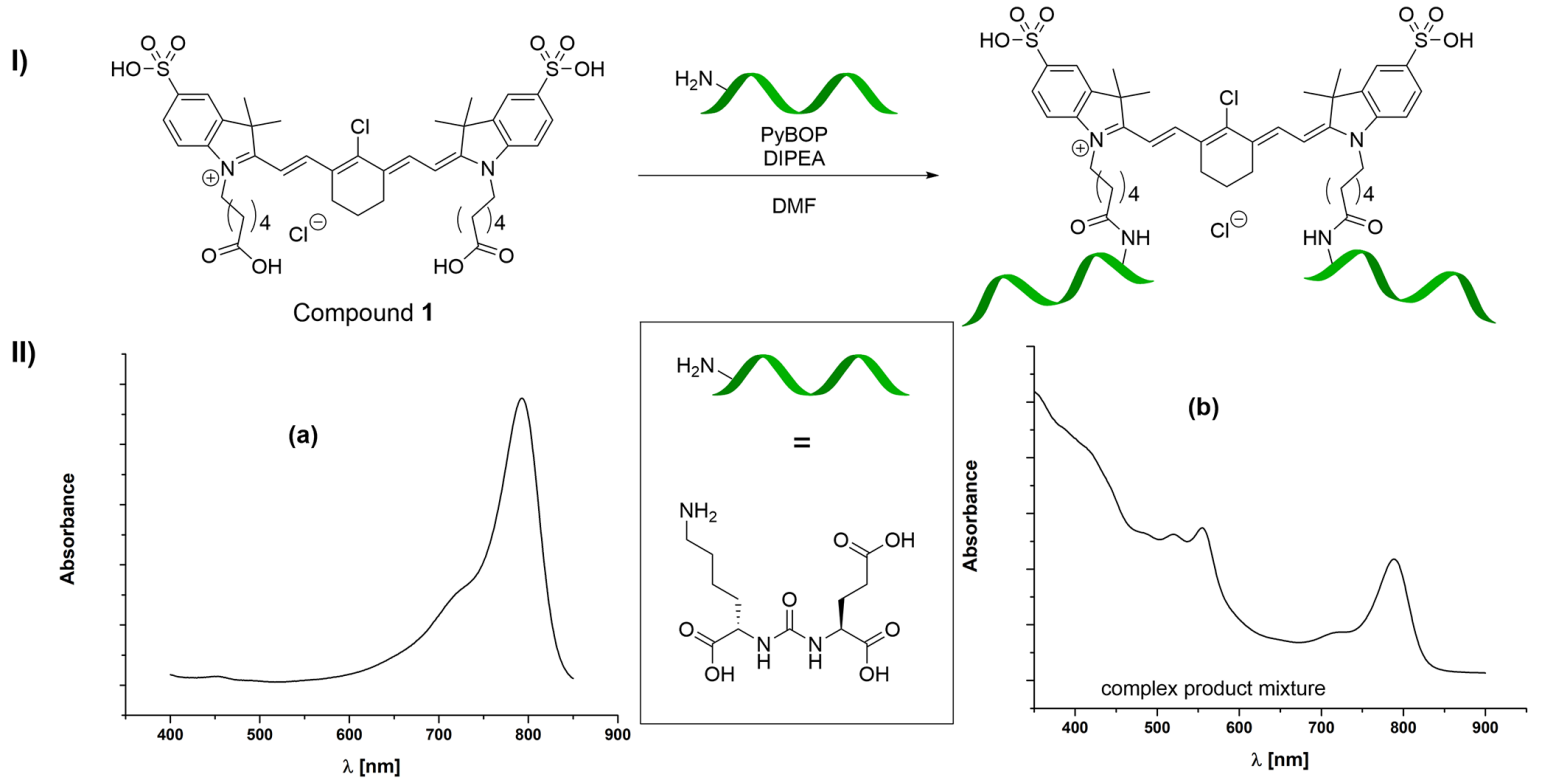
(I) Schematic representation for the attempted reaction formation of a typical amide bond from compound **1**, using standard coupling agents. (II) Excitation spectra of the Compound **1** and that of the corresponding reaction mixture toward the amide bond formation and the synthesis of **1-EuK** using EuK tag (or LysCOGlu, inset) in the presence of PyBOP. Spectra were recorded (a) before and (b) after the peptide coupling reaction was carried out. Further details are given in the Supporting Information.

Compound **1** has limited solubility in common organic solvents: it is possible that certain cyanine/solvent combinations could limit the formation of the keto-polymethine or indeed lead to other products or product mixtures. The underlining process for its reactivity in coupling chemistry is expected to involve the addition of *N*-hydroxy-based coupling agents to the *meso*-Cl position, followed by the N–O bond homolysis to proceed to the ketone product, although the exact mechanism has not been established to date.

These coupling reactions were performed at room temperature and under conditions suitable for coupling chemistry for the formation of bioconjugates: as such further optimization and work with the control of the kinetic rather than thermodynamic factors involved may be necessary to fully assign the transformation pathways involved.

Interestingly, compound **1** reacted with glutathione in a straightforward manner, giving rise to a discrete, new *meso*-substituted compound (**3**, which was isolated and fully characterized spectroscopically, as shown in the Experimental Section), whereas compound **2** did not react with glutathione under the same conditions, indicating stability toward endogenous N- and S-based nucleophiles.

Further investigations on the isolated keto-polymethine **2** indicated that the acidic pH of the initially used eluent mixtures containing 0.1% trifluoroacetic acid (TFA) led to protonation and subsequently to decomposition of dye **2** and its derivatives, which we postulate as the reason deemed to complicate purification by semiprep HPLC. We observed that the use of TFA, at concentrations commonly used in standard HPLC methods (0.1 vol %), leads to protonation, evident from a significant bathochromic shift in the absorption spectroscopy, and subsequent decomposition, evidenced by absorption spectroscopy and mass spectrometry. We also noted that the use of traces of TFA in the standard HPLC purification leads to decomposition, whereas mild acid conditions confirm the expected conversion of the cyanine ketone derivatives to hydroxy cyanines (Supporting Information). However, this well-documented pH dependence did not form the topic of this investigation into coupling chemistries involving the peptide bond formation at the exocyclic substituents. We discarded photobleaching as the primary reason of decomposition for the protonated compound, as samples dissolved in water and acidified with 0.1 vol % TFA decomposed at a similar rate, irrespective of whether they were exposed to light or kept in the dark (Figures 3 and S12–S14). In organic solvents, their stability was markedly increased, suggesting an involvement of water in the decomposition. Based on these observations, a slightly modified work-up procedure was subsequently used, as follows: The dye was loaded onto the cartridge again and washed with a 3 M solution of NaCl in water, before being eluted with an unmodified mobile phase (deionized water/acetonitrile gradient, 0–50% CH_3_CN). This procedure allowed the isolation of the new dye, compound **2** on a milligram scale, which was then characterized by ^1^H NMR spectroscopy, including DOSY, high-resolution mass spectrometry (ESI-HRMS, negative mode), UV–vis, and fluorescence spectroscopies, and deemed to be of purity >95% by integration in HPLC. Optimized geometries for simplified analogues of **1** and **2**, featuring *N*-methyl substituents, instead of *N*-hexanoic acid substituents, were investigated by computational approaches (TDDFT, with details given in the Supporting Information).

**Figure 3:**
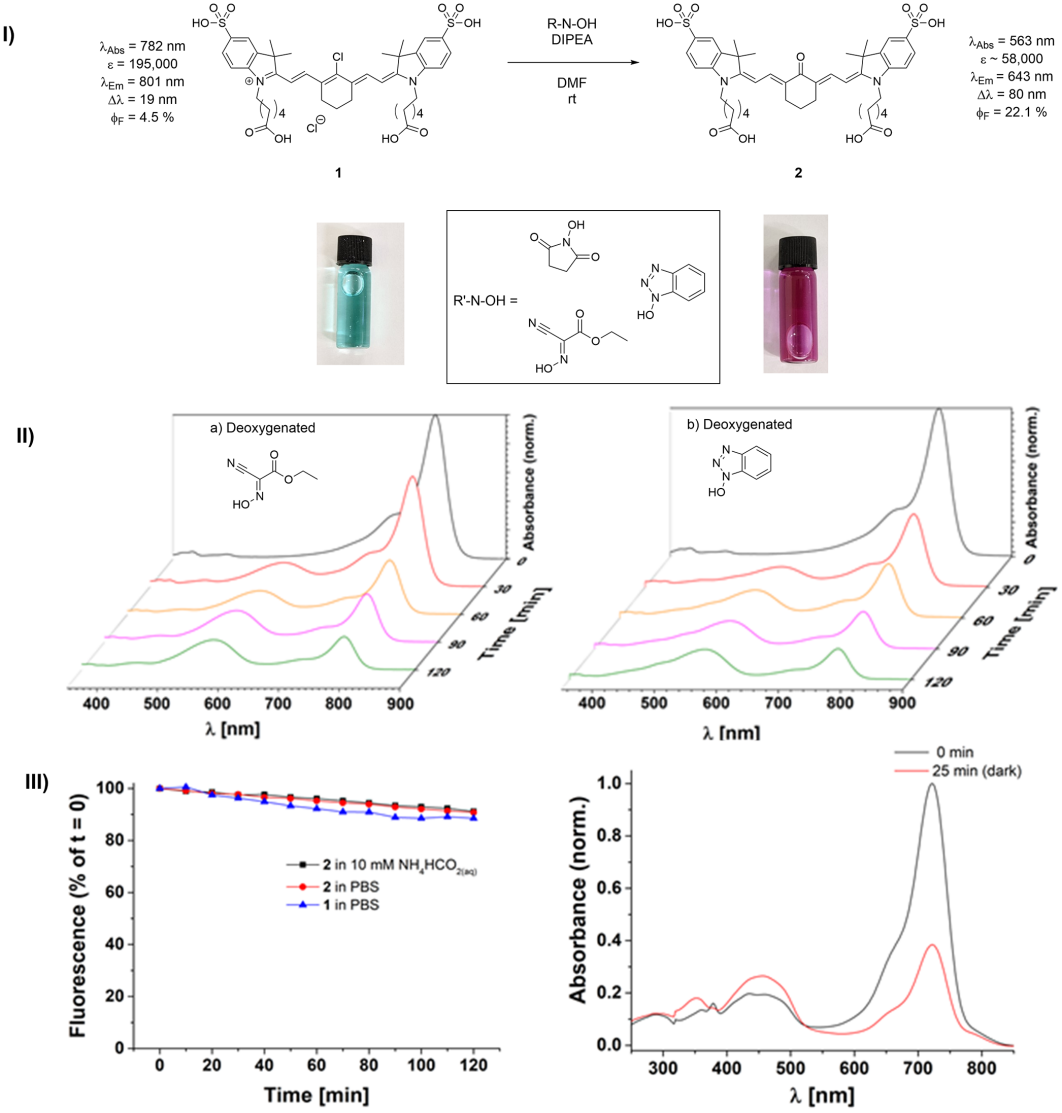
(I) Proposed reaction of *meso*-Cl dyes with *N*-hydroxy-based coupling agents and corresponding spectroscopic evidence upon formation of keto-polymethine as the main product. (II) Reactions monitoring through UV–vis spectroscopy: normalized absorption spectra in 30 min intervals of reactions carried out in deoxygenated solutions containing 3.6 mM **1**, 20 mM DIPEA, and 2 equiv of coupling agent, i.e., (a) [7.2 mM] *K*-Oxyma Pure in deoxygenated DMF at room temperature and (b) [7.2 mM] HOBt·H_2_O in deoxygenated DMF at room temperature. (III) Left: kinetic stability observations using fluorescence emission spectroscopy of **1** in PBS and **2** in various buffers (10 mM ammonium formate in water or PBS), and right: UV–vis spectroscopy suggested the decomposition of **2** in acidic environments (0.1 vol % TFA in water).

Measurements of the optical properties of **2** in PBS suggested a molecular brightness of ∼12,800 at low μM concentrations: a comparison with Compound **1** is given in Figure 3. For a wider comparison, the molecular brightness of the clinically used PpIX gives values below 10,000 in phosphate buffered saline (PBS) and fetal bovine serum (FBS).^37^ The presence of the oxo-substituted framework does not lead to NIR-emitting fluorophores; however, they may be of interest as microscopy tools in a wide range of life sciences applications, *e.g.*, biochemical research tools. Given the success of fluorescein and protoporphyrin IX in fluorescence-guided surgery, the lack of NIR fluorescence may not be prohibitive of *in vivo* use.^38,39^ The related *meso*-amine dyes, which also show blue-shifted absorption and emission profiles, tend to give lower molecular brightness but are of interest to biomedical applications.^11,40,41^ MTT assays with dye **2** indicated that this does not cause a reduction in metabolic activity in PC3 cells (Figure S42) and laser confocal imaging indicated a rather low cellular uptake overall (Figure 4 and the Supporting Infomation).

**Figure 4:**
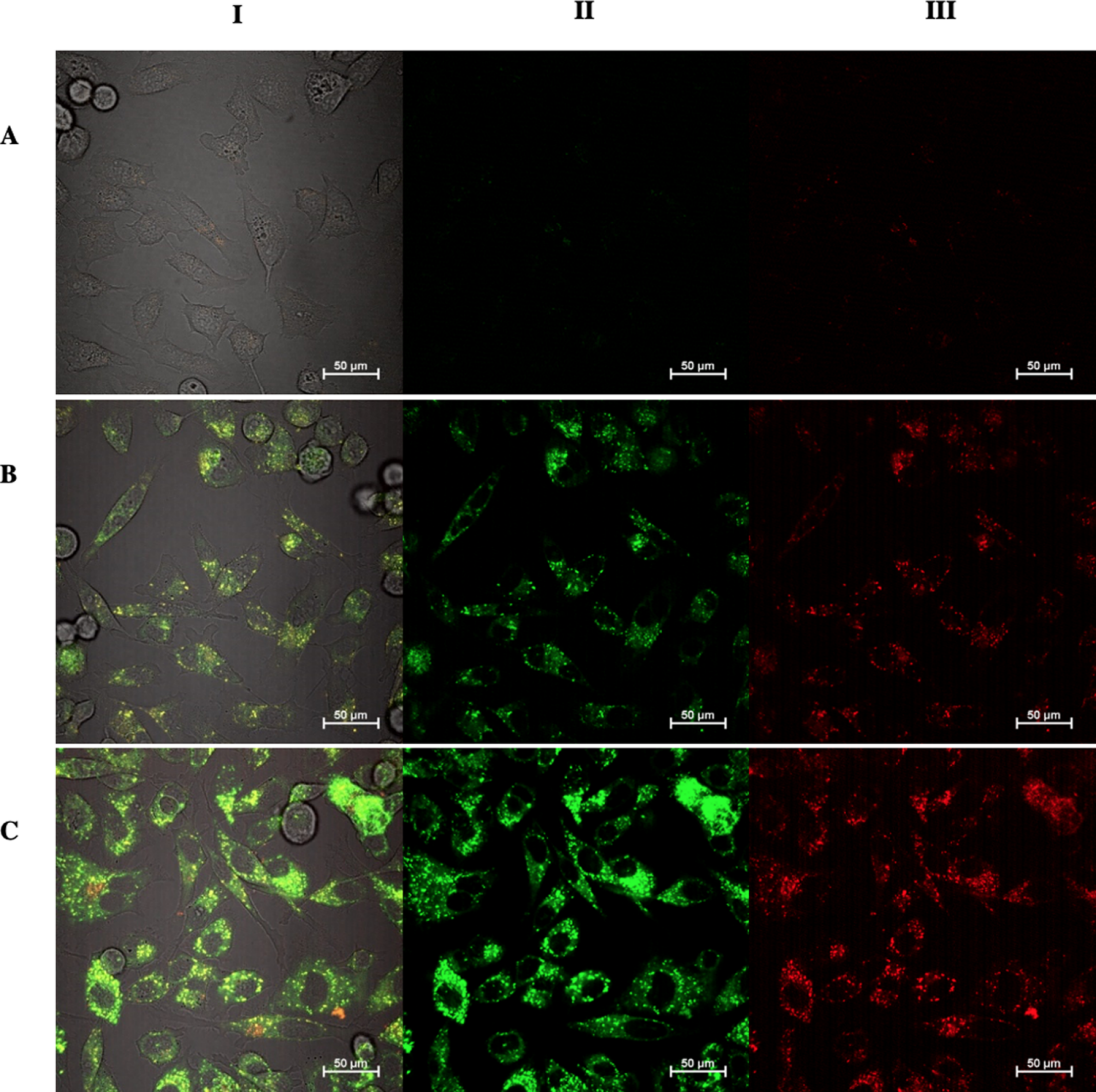
Laser scanning confocal fluorescence imaging of the uptake of **2** and **4** in PC3 cells after 2 h incubation, 37 °C (final concentration 100 μM), at excitation 488 nm. (A) Control experiments, (B) compound **2**, and (C) compound **4**. Emission channels from left to right: (i) overlay of blue, green, and red emission channels with DIC channel; (ii) green channel (λ_em_ = 516–530 nm); and (iii) red channel (λ_em_ = 516–530 nm). The blue channel did not show any emission and was omitted. Scale bar = 50 μm. Additional micrographs at 10 μM concentration in PC3 and DU145 lines, as well as other incubation times are given in the Supporting Information.

Interestingly, upon treating the well-studied, commercial dye **MHI-148** with either HOBt or *K*-Oxyma in DMF, the reactions with these standard coupling agents appeared to proceed much faster than for the case of **1**, and typically reached completion within 30 min, giving rise to the analogous species to **2** on an analytical scale (Supporting Information and Figure S1). The scale-up reaction protocols for the resulting keto-polymethine derivative of **MHI-148** (denoted **MHI-148-O**) proved difficult in our hands. The exclusive use of carbodiimide coupling agents failed to give sufficient conversions and instead appeared to lead to the formation of *N*-acylisoureas as side products (Figures S2–S5). Attempts at forming cyanine conjugates using alternative coupling agents, such as propylphosphonic anhydride (T3P), failed. As expected, the solubility of the resulting product emerging from the oxidation of **MHI-148** in PBS was greatly reduced with respect to that of compound **2**. Also, unlike the case of **2**, for the product resulting from the oxidation of **MHI-148**, no fluorescence emission was observed in PBS (Figures 5 and S51): we postulate that a significant level of aggregation occurs in aqueous solutions. This was also suggested by the fact that the same substance in methanol did show a fluorescence response, in agreement with a keto-polymethine structure. Confocal fluorescence images were obtained from living cancer cells (PC3) treated with compound **2** as well as with its non-sulfonated analogue, **MHI-148-O**. Images were recorded in 1 min intervals over 20 min incubation with dyes at 37 °C. Fluorescence microscopy experiments showed only limited emission from cells incubated with **2**. However, at higher concentrations (100 μM) and after longer incubation times (2 h) at 37 °C, a visible cellular uptake was observed for **2** (Figure S49). The low uptake of highly polar keto-polymethines into human cell lines was previously observed with structurally related dyes, and our results further confirm this for prostate cancer cells.^35^ The oxo form of the ubiquitous **MHI-148** (the nonsulfonated analogous of compound **2**, **MHI-148-O**) appears to be readily taken up by cancer cells even at 10 μM concentrations. Other authors have previously noted the influence of structure and charge on the pharmacokinetics of cyanine dyes and cyanine–antibody conjugates.^42,43^ Further cellular imaging micrographs are given in the Supporting Information (Figures S44–S50).

**Figure 5:**
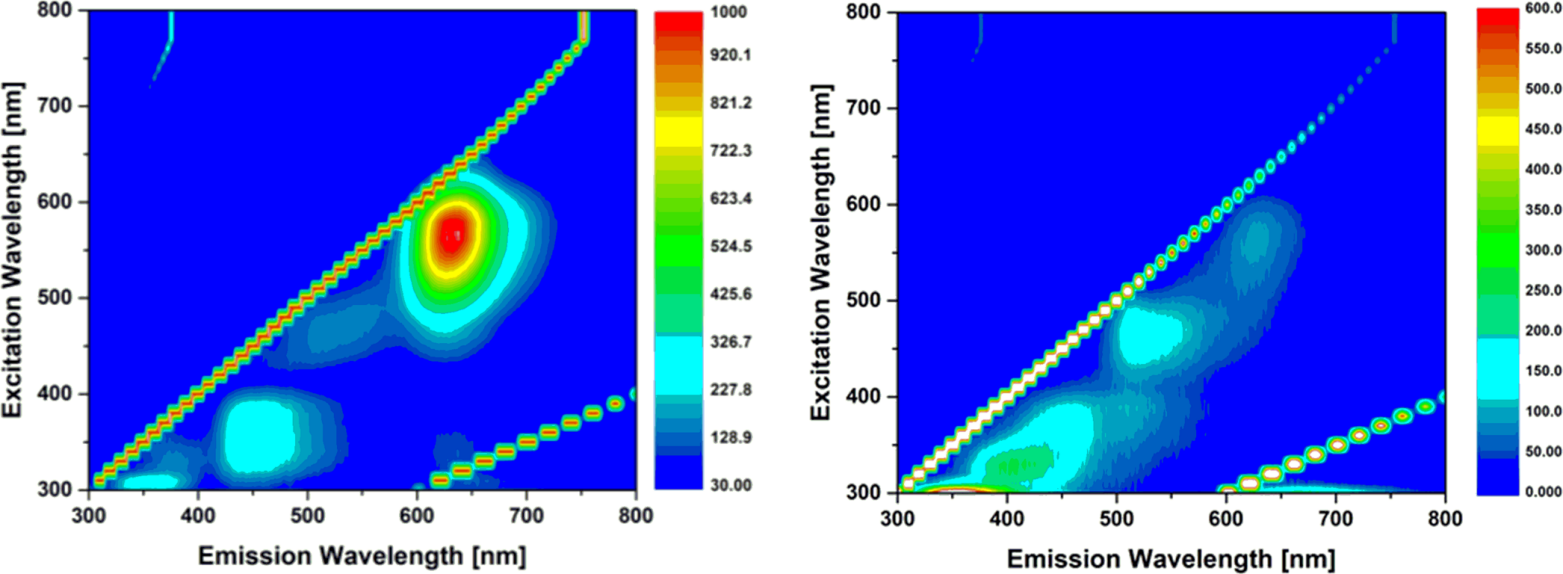
Excitation–emission maps of compound **2** (left) and of the oxo form of **MHI-148**, **MHI-148-O**, (right) at 2 μM conc. in 10% serum medium (10% fetal bovine serum in RPMI) showing a significant blue shift of emission maxima and a significant reduction in the NIR emission properties.

Also, the excitation–emission maps (EEMs) of the merocyanine **2** and the product of the **MHI-148** oxidation, **MHI-148-O**, showed contrasting emission–excitation profiles when measured in PBS or 10% serum medium, as shown in Figure 5. The results suggest that in the presence of proteins, supramolecular interactions occur which lead to the emergence of a distinct, hypsochromically shifted absorption and emission profile (also see the Supporting Information, Figure S51). Pascal et al. previously hypothesized that this emission stems from aprotic environments around the dye and may be used to probe intracellular environments.^35^ Specifically, our experiments suggest that the aggregation to hydrophobic surface regions of proteins may be the central reason for the observed spectroscopic behavior in aqueous environments.

Keto-polymethines may well be used as dyes in multiphoton imaging applications in their own right, as indicated in the state of the art.^35^ Therefore, an in-depth incursion into the formation and isolation of new, water-soluble functionalized keto-polymethine dyes as alternatives to their *meso*-Cl counterparts was deemed worthwhile. A new representative of this class, compound **2**, already showed the advantage that it could be isolated on a milligram scale, in HPLC purity of above 95%. Following the optimization of work-up procedures, its functionalization protocols were developed (see the Experimental Section) and the new, symmetric, glucosamine-functionalized derivative, conjugate **4,** was also synthesized and purified (Figure 6, Experimental Section, and Supporting Information). Its characterization was performed using HPLC, ESI-HRMS, and NMR in line with the standard approaches for other Cy7-based dyes, commercial or noncommercial, and the purity was deemed above 95% according to HPLC quantification at 560 nm detection. The cellular uptake of compound **4** was also investigated under similar conditions to those applied for its precursor, compound **2**. Following cellular uptake experiments in living PC3 cells, statistical analysis of these *in vitro* revealed very limited uptake of compound **4** at low concentration (10 μM) and 30 min incubation time, and this was enhanced at 100 μM concentration and 2 h incubation time at 37 °C (Figure 4). The fact that compound **4** was not internalized more efficiently and rapidly by living cells at the low concentrations tested may be related to the high hydrophilicity of the large cyanine backbone. Previously, it has been reported that water-soluble keto-polymethines were not efficiently taken up by T24 cells and that the uptake of a Cy5.5 glucosamine conjugate was not glucose transporter (GLUT) specific in certain cell lines.^35,44,45^

**Figure 6:**
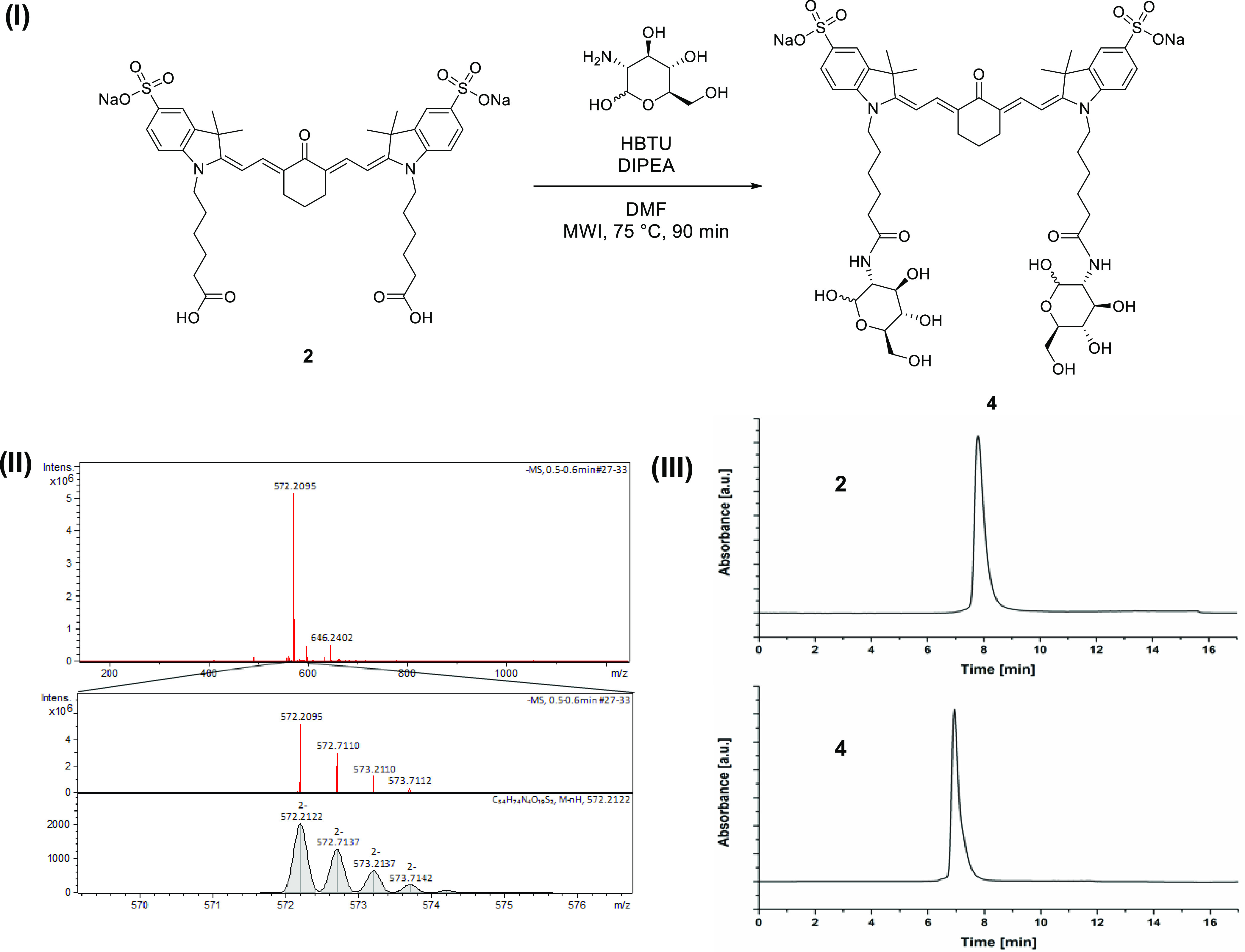
Overview of (I) synthesis of compound **4**, (II) mass spectrometry (ESI-MS, neg. ionization, loop injection) of the isolated product, and (III) analytical HPLC trace for compounds **2** and **4** at 560 nm detection (see the Experimental Section for details on the HPLC methods).

Additionally, the synthesis of a new bioconjugate from the peptide coupling reaction of compound **2** and the [7,13]bombesin fragment led to isolation of compound **5** on the analytical scale, and its characterization only by ESI-HRMS and HPLC. (Supporting Information, Figures S38 and S39). The scale-up synthesis for this compound and the assessment of its ability to target prostate cancer cell lines expressing the gastrin-releasing peptide receptor (GRPR) will be the focus of future work. As highlighted above, the development of small-molecule conjugates of cyanine dyes has been an ongoing endeavor for several decades; however, only very few *meso*-Cl dye conjugates have been described in the literature.^5^ Our results seem to suggest that the reason for this, apart from the generally challenging nature of indocyanine chemistry, resides in the high reactivity of the *meso*-Cl center of these dyes. Future work in our group will focus on alternative methods of synthesizing conjugates of *meso*-Cl dyes: our trials toward the synthesis of peptide bioconjugates of **1** without the use of *N*-hydroxy-based agents also gave mixtures of multiple species. Mass spectroscopy analysis of isolated fractions suggested the presence of *N*-acylurea side products following peptide coupling chemistry protocols (see the Supporting Information): the formation of these may well be suppressed by solvents with lower dielectric constant. Using more recently developed methods to form amide bonds, such as the method developed by Mishra et al.,^46^ may be a way to address the difficult syntheses of conjugates of *meso*-Cl tricarbocyanines. We postulate that the nonpolar solvents necessarily used in many of these reactions would likely be incompatible with the nature of sulfonated cyanine dyes.

In conclusion, we recognized that *meso*-Cl-substituted NIR dyes react with a variety of coupling reagents to give the corresponding keto-polymethines as the major product, as well as other decomposition species, which could not be fully identified in the course of this work. The resulting keto-polymethines are unstable in acidic environments, which may be the reason why these, and their conjugates, have so far been elusive, and the reports on these species in the current literature remain surprisingly scarce, despite the widespread need for dyes of relevance to clinical imaging applications. As stated above, a recent review by Mieog et al. discussed the requirements for developing agents for fluorescence-guided surgery, with a particular focus on biomolecules.^3^ To address this limitation of the state of the art, we developed and reported hereby on our new approach and underlining synthetic method, *e.g.*, to isolate and characterize the keto-polymethines, and their rationally designed, synthesized, and characterized new conjugates, which were discussed hereby. We further investigated the optical properties of some keto-polymethines in biological media, revealing increased fluorescence as well as supramolecular chemistry, which may involve aggregation to proteins. The findings described here are of relevance in the efforts to improve the development of robust and scalable synthetic methodologies for *meso*-Cl cyanine functionalization. This will lead to discrete, well-understood bioconjugated and functional probes that open opportunities for new synthetic chemistry for multimodality imaging applications.

## Experimental Section

### General Experimental Methods

All reactions were performed under ambient atmosphere unless otherwise mentioned. Solvents were peptide (*N*,*N*-dimethylformamide, DMF, and dimethyl sulfoxide, DMSO) or HPLC (acetonitrile, methanol, ethanol) grade. Reagents were obtained commercially and used without further purification. Degassed solvents were obtained using three consecutive freeze–pump–thaw cycles. Deoxygenated solvents were obtained by bubbling argon gas through the respective solvent for 10 min. Solid-phase peptide synthesis (SPPS) was performed using a Biotage Alstra Initiator +. For synthetic operations, 30 mL reactor vials were used according to manufacturer specifications. Fmoc-protected l-amino acids were dissolved in DMF, for a final concentration of 0.6 mol/L. Syntheses of known dyes, compound **1** and **MHI-148**, are based on adapted protocols based on the literature methods,^47−51^ and these are detailed in the Supporting Information.

Chromatography was performed using a Biotage Isolera system equipped with a reverse-phase C_18_-silica cartridge (Sfar Bio C18 – Duo 300 Å 20 mm, 30 g). A gradient of acetonitrile and water or methanol and water was used to purify and elute compounds. For details on buffers used, the reader is referred to the relevant experimental procedure. To remove excess ammonium formate from purified *meso*-oxo dyes, they were eluted once more using an unbuffered mobile-phase. High-performance liquid chromatography (HPLC) was performed using a Dionex UltiMate 3000 preparative system, equipped with a 50 μL loop for analytical and a 2 mL loop for semipreparative work and an eight-channel UV–vis detector (UltiMate 3000 Diode Array Detector). For analytical work, a C_18_-silica column by Hamilton (PRP1, internal diameter 4.1 mm, length 150 mm, particle size 10 μm, pore size 100 Å) was utilized. Integration of chromatograms was performed using commercial Chromeleon software. Methods used were as follows: method A: water/methanol (10 mM ammonium formate in each solvent): 0–1 min, 95:5; 1–5 min, gradient to 25:75; 5–10 min, 25:75; 10–14 min, gradient to 95:5; and 14–19 min, equilibration at 95:5. Method B: water/acetonitrile gradient (0.1 vol % TFA in each solvent): 0–1.5 min, 95:5; 1.5–8.5 gradient to 25:75; 8.5–11.5 min, 25:75; 11.5–12.5 min, gradient to 95:5; and 12.5–16.5 min, equilibration at 95:5.

The final sulfonated dye compounds isolated on a laboratory scale display a purity of >95% (NMR, ESI-HRMS, and HPLC analysis, taken together). The specific batches of the as-synthesized compound **1** and of its new derivatives **2**, **3**, and **4** which were used for biochemical investigations hereby were of analytical purity by integration in HPLC using the Chromeleon software (>95%). All spectroscopic data including NMR, HPLC, and corresponding ESI-HRMS with matched isotopic patterns are given below and in the Supporting Information.

NMR spectra were recorded on a Bruker Neo (400 MHz) spectrometer with SampleCase sample changer. Spectra were measured at 400.13, 376.49, and 100.61 MHz for the acquisition of ^1^H, ^19^F, and ^13^C, respectively. Chemical shifts are reported in ppm, with the solvent residual peak used as an internal standard: DMSO-*d*_6_, δ = 2.50 for ^1^H NMR spectra and δ = 39.52 for ^13^C; CD_3_-OD, δ = 3.31 for ^1^H NMR spectra and δ = 49.00 for ^13^C. Data is reported as follows: s = singlet, d = doublet, t =triplet, m = multiplet, and br = broad. Coupling constants are given in Hz.

High-resolution mass spectra of cyanine dyes were recorded on a Bruker MAXIS HD ESI-QTOF. Parameters are given in the Supporting Information. For mass spectrometry of starting materials, intermediates, and peptides, an automated Agilent QTOF was used. For peptides, an HPLC/MS method was chosen, eluting the injected sample over a reverse-phase C8-HPLC column, with a water/acetonitrile mobile phase (0.1% formic acid). Analysis of mass spectrometry data was performed using commercial Bruker and Agilent software. Deviations between measured *m*/*z* values and predicted *m*/*z* values are given as absolute values in ppm. Fourier-transform infrared (FTIR) spectra were recorded on a PerkinElmer Spectrum 100 IR spectrometer with an attenuated total reflection (ATR) module. The signals listed are those unambiguously assigned to a functional group.

UV–vis spectra were recorded on a PerkinElmer Lambda 650 spectrometer using quartz cuvettes with a path length of 1 cm. Solutions were prepared by dissolving weighed samples of the purified compounds in the appropriate solvent. Dilutions were prepared using standard Eppendorf pipettors. Extinction coefficients were determined with at least four different concentrations using a linear regression according to the Beer–Lambert law. To assess stability toward endogenous species, dilutions were prepared using deionized water either 10 mM glutathione or 1 mM l-ascorbic acid, and the samples were kept in the dark. At various time points (*t* = 0, 2, 4, 8, 24, 72 h), 60 μL aliquots from these solutions were taken, diluted to 1.5 mL, and their absorption was measured.

Fluorescence spectra were recorded on a PerkinElmer LS55 fluorescence spectrometer using quartz cuvettes with a path of 1 cm × 1 cm. Fluorescence maps (EEMs) were recorded with 10 nm excitation increments at a scan speed of 100 nm/min. Relative fluorescence quantum yields were determined with both the PerkinElmer LB650 and the PerkinElmer LS55 spectrometers.

Cell lines used in MTT assays and live cell imaging were prostate cancer cells (PC3 and DU145), obtained from the American Type Cell Culture (ATCC). Cell culturing experimental details are given in the Supporting Information. Before microscopy experiments, cells were seeded onto sterile glass dishes and incubated for 48 h before addition of fluorescent compounds to allow them to adhere to the surface. Confocal microscopy was performed using a Nikon Eclipse Ti2-E inverted confocal microscope with an LU-N3 laser unit (405, 488, and 561 nm).

#### Compound **1**

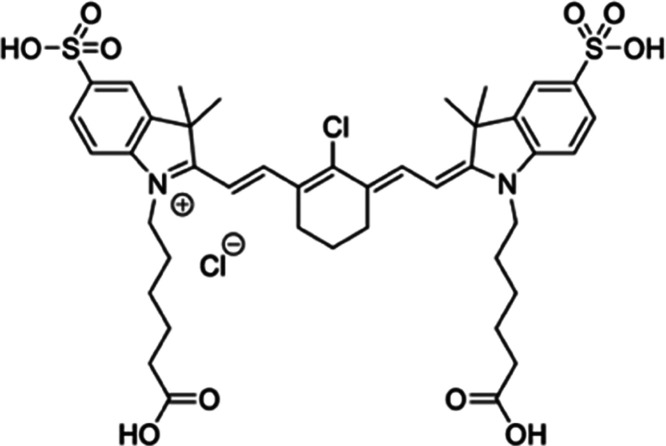



Compound **1** was isolated on a 1.35 g scale overall, 18% yield using an adapted multistep protocol, as described in the Supporting Information.^47−49^

^1^H NMR (400.13 MHz, DMSO-*d*_6_, 298 K): δ = 8.25 (d, 3JHH = 14.0 Hz, 2H), 7.81 (d, 4JHH = 1.6 Hz, 2H), 7.67 (dd, JHH = 8.2, 1.6 Hz, 2H), 7.39 (d, 3JHH = 8.2 Hz, 2H), 6.33 (d, 3JHH = 14.0 Hz, 2H), 4.21 (t, 3JHH = 7.5 Hz, 4H), 2.70 (d, 3JHH = 6.3 Hz, 4H), 2.20 (t, 3JHH = 7.2 Hz, 4H), 1.95–1.78 (m, 2H), 1.78–1.69 (m, 4H), 1.67 (s, 12H), 1.60–1.48 (m, 4H), 1.47–1.33 (m, 4H).

^13^C{^1^H} NMR (100.16 MHz, DMSO-*d*_6_, 298 K): δ = 174.8, 172.9, 148.5, 146.0, 143.4, 142.6, 141.0, 127.1, 126.7, 120.4, 111.2, 102.5, 49.5, 44.3, 34.0, 27.9, 27.2, 26.3, 26.1, 24.7, 20.8.

ESI-MS: negative mode [*m*/*z*] calculated for C_42_H_51_ClN_2_O_10_S_2_ [M – H]^−^ = 841.2601; found, 841.2579; deviation = 2.62 ppm. Calculated for C_42_H_50_ClN_2_O_10_S_2_ [M – 2H]^2–^ = 420.1264; found, 420.1250; deviation = 3.33 ppm.

UV–vis (PBS): λ_max_(Abs) [nm] (ε [M^–1^ cm^–1^]) = 782 (195,000).

Fluorescence spectroscopy (PBS): λ_Exc_ [nm] = 770, λ_maxEm_ [nm] = 803, ϕF = 0.045 (reference: ICG).

Analytical HPLC: Hamilton C_18_-silica, method A, *t*_R_ = 7.49 min, method B, *t*_R_ = 8.37 min (*ca.* 98% at 780 nm).

#### Compound **2**

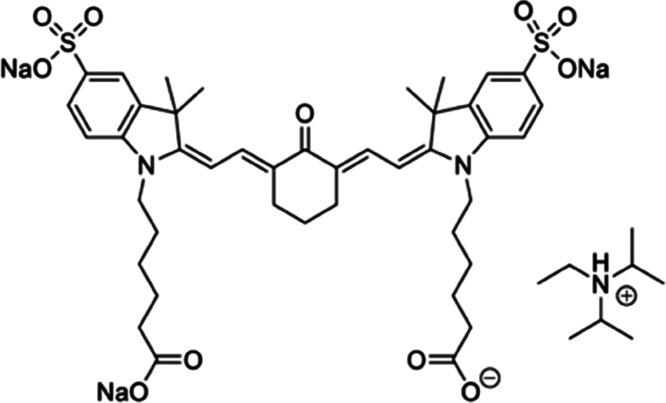



In a 25 mL round-bottom flask, dye **1** (87 mg, 99 μmol) was dissolved in 4 mL of peptide-grade DMF. To this, 83 μL (*ca.* 495 μmol) of DIPEA and 68 mg (594 μmol) of *N*-hydroxysuccinimide were added. The resulting mixture was stirred for 1 h at room temperature, before it was poured into 25 mL of deionized water. The obtained aqueous solution was loaded onto a reverse-phase flash chromatography cartridge and washed with a 3 M solution of sodium chloride in water, before the product was eluted using a water/acetonitrile gradient. Extensive purification by reverse-phase flash chromatography (water/methanol, with 10 mM NH_4_HCO_2_) was also performed on several batches. To remove traces of ammonium from the isolated product, it was loaded onto the reverse-phase cartridge again, washed with 0.1 M NaCl, and subsequently eluted with a water/acetonitrile gradient (unbuffered). As a result of this final purification step, it was assumed that all dyes are obtained as sodium salts. Analysis showed that compound **2** has been isolated in the solid state as a mixed salt with 0.8 equivalents of DIPEA. The obtained product was concentrated under reduced pressure and finally lyophilized to yield **2** as a red powdery solid (56 mg, 65% yield).

^1^H NMR (400.13 MHz, CD_3_OD, 298 K): δ = 8.14 (d, 3JHH = 12.8 Hz, 2H), 7.72 (dd, JHH = 8.3, 1.7 Hz, 2H), 7.70 (d, 4JHH = 1.7 Hz, 2H), 6.89 (d, 3JHH = 8.3 Hz, 2H), 5.65 (d, 3JHH = 13.2 Hz, 2H), 3.81 (t, 3JHH = 7.6 Hz, 4H), 2.67–2.58 (m, 4H), 2.21 (t, 3JHH = 7.5 Hz, 4H), 1.91–1.84 (m, 2H), 1.78–1.65 (m, 20H), 1.51–1.44 (m, 4H). (DIPEA signals not listed here.)

^13^C{^1^H} NMR (100.16 MHz, CD_3_OD, 298 K, chemical shifts determined by HSQC and HMBC): δ = 164.6, 140.6, 139.9, 135.5, 127.5, 120.6, 107.7, 94.9, 47.9, 43.5, 38.2, 28.9, 27.9, 27.0, 26.6, 23.7. (DIPEA signals not listed here, some signals could not be fully assigned from the correlation spectra).

ESI-MS: positive mode (*m*/*z*) calculated for C_42_H_50_N_2_O_11_S_2_ [M – 2H]^2–^ = 411.1433; found, 411.1413; deviation = 4.86 ppm.

UV–vis (PBS): λ_maxAbs_ [nm] (ε [M^–1^ cm^–1^]) = 563 (*ca.* 58,000).

Fluorescence (PBS): λ_Exc_ [nm] = 540, λ_maxEm_ [nm] = 641, ϕF = 0.221 (reference: rhodamine B).

Analytical HPLC: Hamilton C_18_, method A, *t*_R_ = 7.79 min (integration *ca.* 99% at 560 nm).

#### Compound **3**

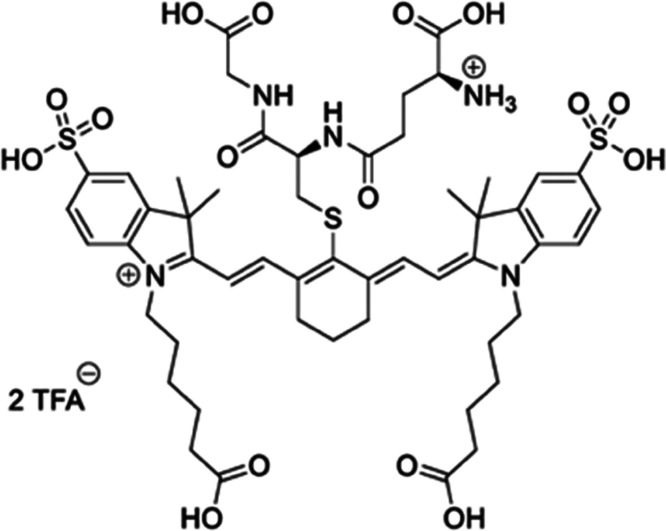



A 7 mL vial was charged with 25 mg (29.6 μmol) of **1**, 4 mL of phosphate-buffered saline, and 25 mg (81 μmol) of reduced glutathione. The mixture was stirred for 4 h at room temperature. Subsequently, the dark green solution was loaded onto a reverse-phase chromatography column (C_18_-silica). The product was eluted using a water/acetonitrile gradient (0% ACN to 50% ACN, 0.1 vol % TFA in water). After removing most of the organic solvent under reduced pressure, the remaining aqueous solution was dried by lyophilization to yield compound **3** as a dark green solid (36 mg, 92% yield).

^1^H NMR (400.13 MHz, DMSO-*d*_6_, 298 K): δ = 8.58 (d, *J* = 14.0 Hz, 2H), 8.50 (q, *J* = 7.9, 6.7 Hz, 1H), 8.33 (t, *J* = 6.0 Hz, 1H), 8.29–8.22 (m, 2H), 7.76 (s, 1H), 7.65 (dd, *J* = 8.2, 1.6 Hz, 2H), 7.35 (d, *J* = 8.4 Hz, 2H), 6.28 (d, *J* = 14.0 Hz, 2H), 4.56–4.45 (m, 1H), 4.24–4.09 (m, 4H), 3.95–3.86 (m, 1H), 3.75–3.69 (m, 2H), 3.15–3.01 (m, 4H), 2.68–2.57 (m, 4H), 2.40–2.29 (m, 3H), 2.20 (t, 3JHH = 7.2 Hz, 4H), 2.03–1.92 (m, 2H), 1.90–1.78 (m, 2H), 1.77–1.49 (m, 20H).

ESI-MS: negative mode [*m*/*z*] calculated for C_52_H_67_N_5_O_16_S_3_ [M – H]^−^ = 1113.3745; found, 1113.3700; deviation = 4.04 ppm. Calculated for C_52_H_66_N_5_O_16_S_3_ [M – 3H]^2–^ = 555.6800; found, 555.6806; deviation = 1.08 ppm.

UV–vis (PBS): λ_max_(Abs) [nm] (ε [M^–1^ cm^–1^]) = 793 (198,000).

Fluorescence spectroscopy (PBS): λ_Exc_ [nm] = 740, λ_maxEm_ [nm] =, ϕF = 0.041 (reference ICG).

Analytical HPLC: Hamilton C_18_, method B, *t*_R_ = 7.62 min (integration 96% at 780 nm).

#### Compound **4**

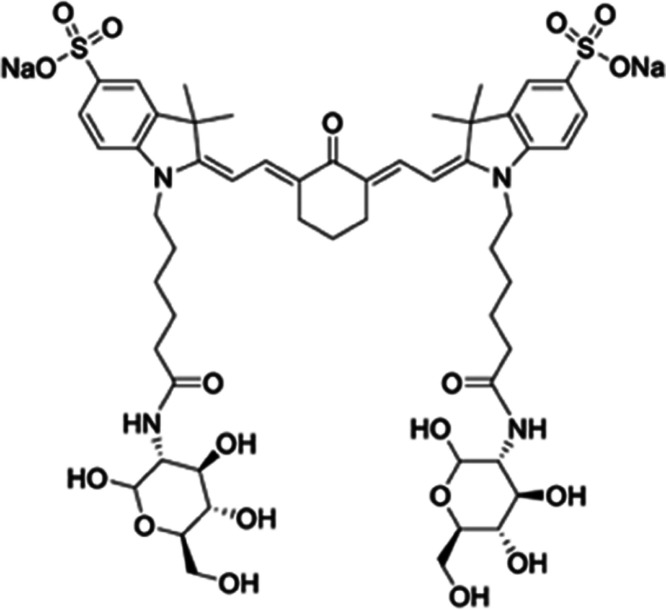



In a microwave vial, compound **2**, synthesized as above (12 mg, 14 μmol), was dissolved in 2.5 mL of DMF. To this, HBTU (30 mg, 79 μmol) was added, followed by DIPEA (23 μL, *ca.* 140 μmol) and glucosamine hydrochloride (20 mg, 92 μmol). The mixture was heated to 75 °C under microwave irradiation for 90 min. After cooling to room temperature, the mixture was poured into 20 mL of deionized water. The solution was loaded on a reverse-phase flash chromatography column and eluted with a water/MeOH gradient (10 mM ammonium formate buffer in mobile phases). The obtained product fraction was concentrated under reduced pressure to remove excess methanol. The obtained aqueous solution was loaded onto a reverse-phase flash chromatography cartridge and washed with a 3 M solution of sodium chloride in water before the product was eluted with unbuffered methanol. The obtained product was concentrated under reduced pressure and finally lyophilized to yield compound **4** as a red solid (11 mg, 66%).

^1^H NMR (400.13 MHz, CD_3_OD, 298 K): δ = 8.14 (d, 3JHH = 13.2 Hz, 2H), 7.74–7.70 (m, 4H), 6.91–6.86 (m, 2H), 5.65 (d, 3JHH = 13.3 Hz, 2H), 3.87–3.76 (m, 8H), 3.72–3.67 (m, 2H) 2.64–2.59 (m, 4H), 2.31–2.18 (m, 8H), 1.90–1.84 (m, 2H), 1.76–1.65 (m, 24H), 1.48–1.42 (m, 4H).

ESI-MS: negative mode (*m*/*z*) calculated for C_54_H_72_N_4_O_19_S_2_ [M – 2H]^2–^ = 572.2122; found, 572.2095; deviation = 4.72 ppm.

UV–vis (PBS): λ_maxAbs_ [nm] (ε [M^–1^ cm^–1^]) = 562 (approx. 56,000).

Fluorescence (PBS): λ_Exc_ [nm] = 540, λ_maxEm_ [nm] = 641, ϕF = 0.204.

Analytical HPLC: Hamilton C_18_, method A, *t*_R_ = 6.83 min (integration *ca.* 98% at 560 nm).

#### Compound **5**

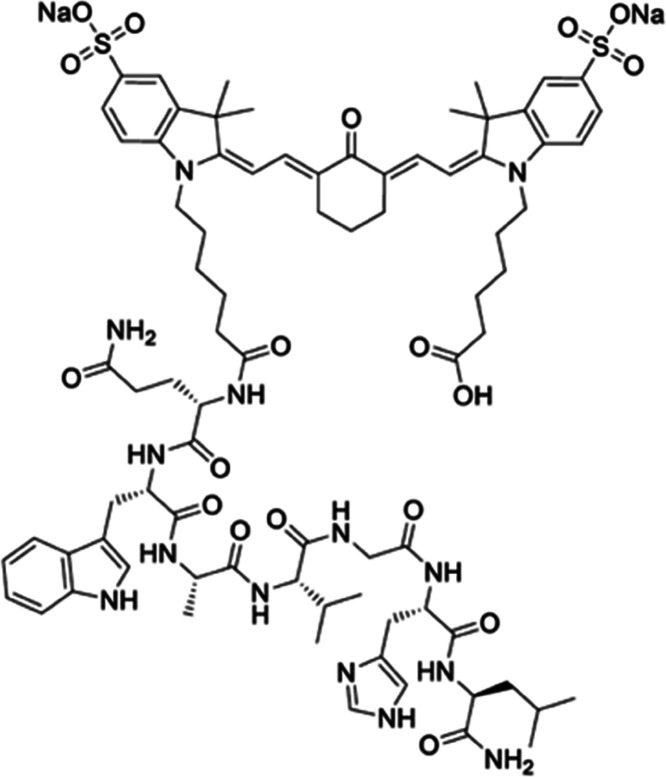



In a microwave vial, compound **1** (6 mg, 6.8 μmol) was dissolved in 1.5 mL of DMF. To this, HBTU (3 mg, 7.9 μmol) was added, followed by DIPEA (3 μL, 17 μmol) and [7, 13]bombesin (5 mg, 5.4 μmol). The mixture was heated to 50 °C under microwave irradiation for 90 min. After cooling to room temperature, the mixture was poured into 20 mL of deionized water and filtered over a pad of C_18_-silica, which was washed with a 1:1 mixture of acetonitrile and water to elute trapped product. The solution was loaded on a reverse-phase flash chromatography column and eluted with a water/MeOH gradient (10 mM ammonium formate buffer in mobile phases). The obtained product fraction was concentrated under reduced pressure to remove excess methanol. The obtained aqueous solution was loaded onto a reverse-phase flash chromatography cartridge and washed with a 3 M solution of sodium chloride in water before the product was eluted with a gradient of deionized water and methanol (0% MeOH to 75% MeOH). The obtained product was concentrated under reduced pressure and finally lyophilized to yield compound **5** as a red solid, isolated on the analytical scale only (3.7 mg). Integration in the analytical HPLC was approximate due to significant peak broadening assigned to protonation, in line with our previously reported conjugates of [7, 13]bombesin.^52^

ESI-MS: negative mode (*m*/*z*) calculated for C_80_H_104_N_14_O_19_S_2_ [M – 2H]^2–^ = 806.3553; found, 806.3515; deviation = 4.71 ppm.

Analytical HPLC: Hamilton C_18_, method A, *t*_R_ = 8.29 min (integration *ca.* 91–95% at 560 nm).

## Data Availability

The available data is included in the Supplementary Information published with the article.

## Supporting Information Available

File denoted the ESI in manuscript contains detailed description of experimental methods and characterization of new compounds, including HPLCs, ^1^NMR, UV–vis, fluorescence, and ESI-HRMS spectrometry data; additional cellular assays; imaging micrographs, and density functional theory (DFT) calculation details (PDF)

## Abbreviations

DIPEA: *N*,*N*-diisopropylethylamine
DMF: *N*,*N*-dimethylformamide
ESI-MS: electrospray ionization mass spectrometry
GLUT: glucose transporter
GRPR: gastrin-releasing peptide receptor
HOBt: *N*-hydroxybenzotriazole
HPLC: high-performance liquid chromatography
NHS: *N*-hydroxysuccinimide
NIR: near-infrared
NMR: nuclear magnetic resonance
PBS: phosphate-buffered saline
PyBOP: benzotriazol-1-yloxytripyrrolidinophosphonium hexafluorophosphate
