# Supplementary material for: Unraveling the Chemistry of meso-Cl Tricarbocyanine Dyes in Conjugation Reactions for the Creation of Peptide Bonds
Source: ACS Bio Med Chem Au. 2022 Nov 8;2(6):642–54. doi: 10.1021/acsbiomedchemau.2c00053 (PMC9782398; doi:10.1021/acsbiomedchemau.2c00053)

Supporting Information

Unraveling the Chemistry of meso-Cl Tricarbocyanine Dyes in Conjugation Reactions for the Creation of Peptide Bonds

Rüdiger M. Exner[a], Fernando Cortezon-Tamarit[a], Haobo Ge[a], Charareh Pourzand[b, c] and Sofia I. Pascu*[a, c]

[a] Rüdiger M. Exner, Dr. Fernando Cortezon-Tamarit, Dr. Haobo Ge, Prof. Sofia I. Pascu
Department of Chemistry
University of Bath
Claverton Down Rd., BA2 7AY, Bath, United Kingdom
E-mail: S.Pascu@bath.ac.uk

[b] Dr. Charareh Pourzand
Department of Pharmacy and Pharmacology
University of Bath
Claverton Down Rd., BA2 7AY, Bath, United Kingdom

[c] Prof. Sofia I. Pascu and Dr. Charareh Pourzand
Centre of Therapeutic Innovations
University of Bath
Claverton Down Rd., BA2 7AY, Bath, United Kingdom

Contents

Materials and Methods 3

Reagents and Solvents *3*

General Methods *3*

Cell Culturing and Microscopy *6*

Cell Culture Methods 6

Cell Viability Assays 6

Laser Confocal Fluorescence Microscopy 7

Experimental Section 8

Synthesis of **1** 10

Synthesis of **2** 16

Synthesis of **3** 16

Synthesis of **4** 17

Synthesis of **5** 17

MHI-148 and its Derived Keto-Polymethine 20

Kinetic Stability of Keto-Polymethine 2 41

Spectroscopic characterization Data for Compounds ***1-5*** 49

In Vitro Assays 65

Biological activity of investigated dyes using standard metabolic inhibition assays *65*

Optical Properties in Biological Media 74

DFT and TD DFT Calculations 75

References 86

Materials and Methods

Reagents and Solvents

All reactions were performed under ambient atmosphere and monitored by UV/VIS spectroscopy or analytical HPLC. Solvents were peptide (N,N-Dimethylformamide, DMF and dimethyl sulfoxide, DMSO) or HPLC (acetonitrile, methanol, ethanol) grade. Reagents were obtained commercially and used without further purification.

General Methods

Solid Phase Peptide Synthesis (SPPS) was performed using a *Biotage Alstra Initiator +*. For synthetic operations, 30 mL reactor vials were used according to manufacturer specifications. Fmoc-protected L-amino acids were dissolved in DMF, for a final concentration of 0.6 mol/L.

Flash Chromatography was performed using a *Biotage Isolera* system equipped with a reverse-phase C_{18}-silica cartridge (*Sfar Bio C18 – Duo 300 Å 20 µm, 30 g*). A gradient of 5 to 95 % acetonitrile in water was used to purify and elute compounds. For meso-Cl dyes, mobile phases were modified with 0.1 vol% trifluoroacetic acid, while for meso-O dyes, mobile phases were 10 mM solutions of ammonium formate. To remove excess ammonium formate from purified *meso*-O dyes, they were eluted once more using an unbuffered mobile phase. NMR spectra measured in DMSO-*d6* showed no signs of residual ammonium salts.

High Performance Liquid Chromatography (HPLC) was performed using a *Dionex UltiMate 3000* preparative system, equipped with a 2 mL loop and an eight-channel UV/VIS detector. For compounds 1-4, mobile phases were modified with 10 mM solutions of ammonium formate, as the use of the classical 0.1 % TFA mixture led to decomposition of the keto-polymethines. A C_{18}-silica column by Hamilton (PRP1, internal diameter 4.1 mm, length 150 mm, particle size 10 µm, pore size 100 Å) was used. All cyanine dyes eluted using method A: 0.9 mL/min, H_2O/MeOH (10 mM ammonium formate) in vol%; 0 - 1 min, 95/5; 1 - 5 min, 95/5 to 25/75; 5 -10 min, 25/75; 10-14 min, 25/75 to 95/5; 14 - 18 min, 95/5. For bombesin [7-13], method B was utilized: 0.9 mL/min H_2O/MeOH (0.1 vol% TFA in each solvent) in vol%: 0 - 1.5 min, 95/5; 1.5 – 8.5 gradient to 25/75; 8.5 – 11.5 min, 25/75; 11.5 – 12.5 min, gradient to 95/5; 12.5 – 16.5 min, equilibration at 95/5.

NMR spectra were recorded on a *Bruker Neo* (400 MHz) spectrometer with *SampleCase* sample changer. Spectra were measured at 400.13 MHz and 100.61 MHz for the acquisition of ^{1}H and ^{13}C, respectively. Chemical shifts are reported in ppm, with the solvent residual peak used as an internal standard: $(CD_3)_2SO$, δ = 2.50 for ^{1}H-NMR spectra and δ = 39.52 for ^{13}C; CD_3OD, δ= 3.35, 4.78 for ^{1}H-NMR spectra and δ = 49.3 for ^{13}C. Data is reported as follows: s = singlet, d = doublet, t =triplet, m = multiplet, br = broad. Coupling constants are given in Hz.

High resolution mass spectra were recorded on a *Bruker MAXIS HD ESI-QTOF*. Parameters used are given in Table S1.

Table S1. Parameters for HR-MS.

Parameter	Negative
Funnel 1 RF	300.00 Vpp
isCID Energy	0.0 eV
Ion Energy	3.0 eV
Collision Energy	3.0 eV
Collision RF	500.0 Vpp
Transfer Time	120 μs
Pre Pulse Storage	14.0 μs
End Plate Offset	500 V
Capillary	4500 V
Nebulizer	0.6 Bar
Dry Gas	6.0 L/min
Dry Temp.	260 °C

Errors relative to the simulated mass spectra are reported as absolute values.

FT-IR spectra were recorded on a *PerkinElmer Spectrum 100 IR* spectrometer with an attenuated total reflection (ATR) module. The signals listed are those unambiguously assigned to a functional group.

UV/VIS spectra were recorded on a *PerkinElmer Lambda 650* spectrometer using quartz cuvettes with a path length of 1 cm. Solutions were prepared by dissolving weighed samples of the purified compounds in the appropriate solvent. Dilutions were prepared using standard *Eppendorf®* pipettors. Extinction coefficients were determined with at least four different

concentrations using a linear regression according to the *Beer-Lambert* law. To assess stability towards endogenous species, dilutions were prepared using deionized water containing 100 μM of the respective dye, and 10 mM glutathione or 1 mM L-ascorbic acid or 50 vol% fetal bovine serum (FBS) as solvent. The samples were kept in the dark. At various time points (t = 0 h, 2 h, 4 h, 8 h, 24 h, 72 h), 60 μL aliquots from these solutions were taken, diluted to 1.5 mL and their absorption spectra were measured.

Fluorescence spectra were recorded on a *PerkinElmer LS55* spectrometer using quartz cuvettes with a path length of 1 cm x 1 cm. Fluorescence maps were recorded with 10 nm excitation increments at a scan speed of 100 nm/min. Relative fluorescence quantum yields were determined with both, the *PerkinElmer LB650* and the *PerkinElmer LS55* spectrometers. The following is a general procedure:

A sample of the dye was dissolved in an appropriate solvent, so that the absorption maximum had a value of A < 0.1. It is important to use such low concentrations to avoid inner filter effects, especially in dyes with small Stokes shifts. Subsequently, the emission spectrum of the dye, excited at an appropriate wavelength was measured.

Excitation wavelength and slit widths need to be kept constant during these measurements. The absorbance of the sample and the standard at the excitation wavelengths were determined. The emission spectra were integrated. Then, the relative fluorescence quantum yields could be determined using the formula:

$$\phi_F = \phi_{ref} \cdot \frac{\eta^2_{sample}}{\eta^2_{ref}} \cdot \frac{E_{sample}}{A_{sample}} \cdot \frac{A_{ref}}{E_{ref}} \qquad \textbf{(S-I)}$$

Where η is the refractive index of the respective solvent, E is the area under the curve of the emission spectrum, and A is the absorbance at the excitation wavelength.

In cases where the same solvent was used for sample and reference, the term

$$\frac{\eta^2_{sample}}{\eta^2_{ref}}$$

in equation **S-I** equals one and may be omitted. Indocyanine Green in DMSO (for **1**) and Rhodamine B in MeOH (for **2**) were used as references.

MHI-148 was synthesized according to a literature procedure.[1, 2]

Density Functional Theory (DFT) and time-dependent density functional theory (TDDFT) calculations were performed using the Amsterdam Density Functional (ADF) suite. All calculations performed in the gas phase, before using the continuous solvation model COSMO (conductor like screening model). It was used to model water as the solvent (dielectric constant

ε = 78.39, radius of rigid-sphere solvent molecules r = 1.93). Calculations were performed at the BLYP/TZP level of theory. No frozen cores were applied. Geometries were optimized and analytical frequencies calculated, before allowed singlet-singlet transitions were modelled at the TDDFT level of theory using the TDDFT+TB (tight binding) method [8-16].

Cell Culturing and Microscopy

Cell Culture Methods

Cell lines used in live cell imaging were prostate cancer cells PC3 and DU145 cells. All cell lines were obtained from American Type Cell Culture (ATCC). Cells were normally frozen at -196 °C in liquid nitrogen until required, then thawed quickly and incubated after the addition of fresh media at 37 °C under 5% carbon dioxide environment. All solvents, buffer solutions and media mentioned in the following section were warmed to 37 °C in the water bath prior to addition. Eagle's Minimum Essential Medium (EMEM) and Roswell Park Memorial Institute (RPMI) medium were used as culture media. All media contained activated fetal calf serum (FCS) (10% for both, PC-3 and DU145), 0.5% penicillin/streptomycin (10,000 IU mL^{-1}/10,000 mg mL^{-1}) and 2.5% l-glutamine.

Cell subculture was performed once or twice per week depending on the confluence of the cells in the flask, the supernatant was aspirated. All attached cells were washed twice using phosphate buffered saline (PBS, 2 x 10 mL), 3.5 mL of 0.25% trypsin in PBS was subsequently loaded and incubated at 37 °C for 5 minutes. After trypsinization, 7 mL serum medium was added to neutralize the excess trypsin and the solution was centrifuged at 1000 rpm for 5 minutes. Afterwards, the supernatant was aspirated and resuspended with 5 mL serum medium. Cells were counted in a haemocytometer and seeded appropriately for cell viability assays.

Cell Viability Assays

Cell Viability Assays were performed in the skin fibroblast cell line FEK4 and the prostate cancer cell line PC3. After cell splitting, cells were seeded on a sterile 96 well plate (7×10^3 cells per well) and incubated for 48 hours to adhere. Compounds were subsequently loaded at different concentration into wells and cultured for another 48 hours. The concentration used were 250 μM (1% DMSO, 99% culture media (10% FCS)), 100 μM, 50 μM, 10 μM, 1 μM, 0.5 μM, 0.1 μM and 1 nM. After 24 hours incubation, cells were washed

three times with PBS (1 mL) and 3-(4, 5-dimethylthiazol-2-yl)-2, 5-diphenyltetrazolium bromide (MTT) was added (0.5 mg/ mL, 1:9 PBS: serum-free medium (SFM)) followed by a two-hour incubation. After aspiration, DMSO (100 µL) was added and 96 well plates were read by an ELISA plate reader, Molecular Devices Versa Max (BN02877). The absorption wavelength was set at 570 nm, with 630 nm used as a reference.

Laser Confocal Fluorescence Microscopy

Prior to microscopy experiments, cells were seeded onto sterile glass dishes, and incubated for 48 h prior to addition of fluorescent compounds, to allow them to adhere to the surface. A stock solution of 10 mM (or, in other sets of experiments, 1 mM) solution of the dye in DMSO was added, for a final concentration of either 100 μM or 10 μM on the cells plate with 1% DMSO to 99% culture media. Fluorescence Microscopy was performed in the Bioimaging and Cell Analysis Suite of the Materials and Characterization facility (MC^2). Confocal microscopy was performed using a Nikon Eclipse Ti2-E inverted confocal microscope with an LU-N3 laser unit (405, 488 and 561 nm).

Experimental Section

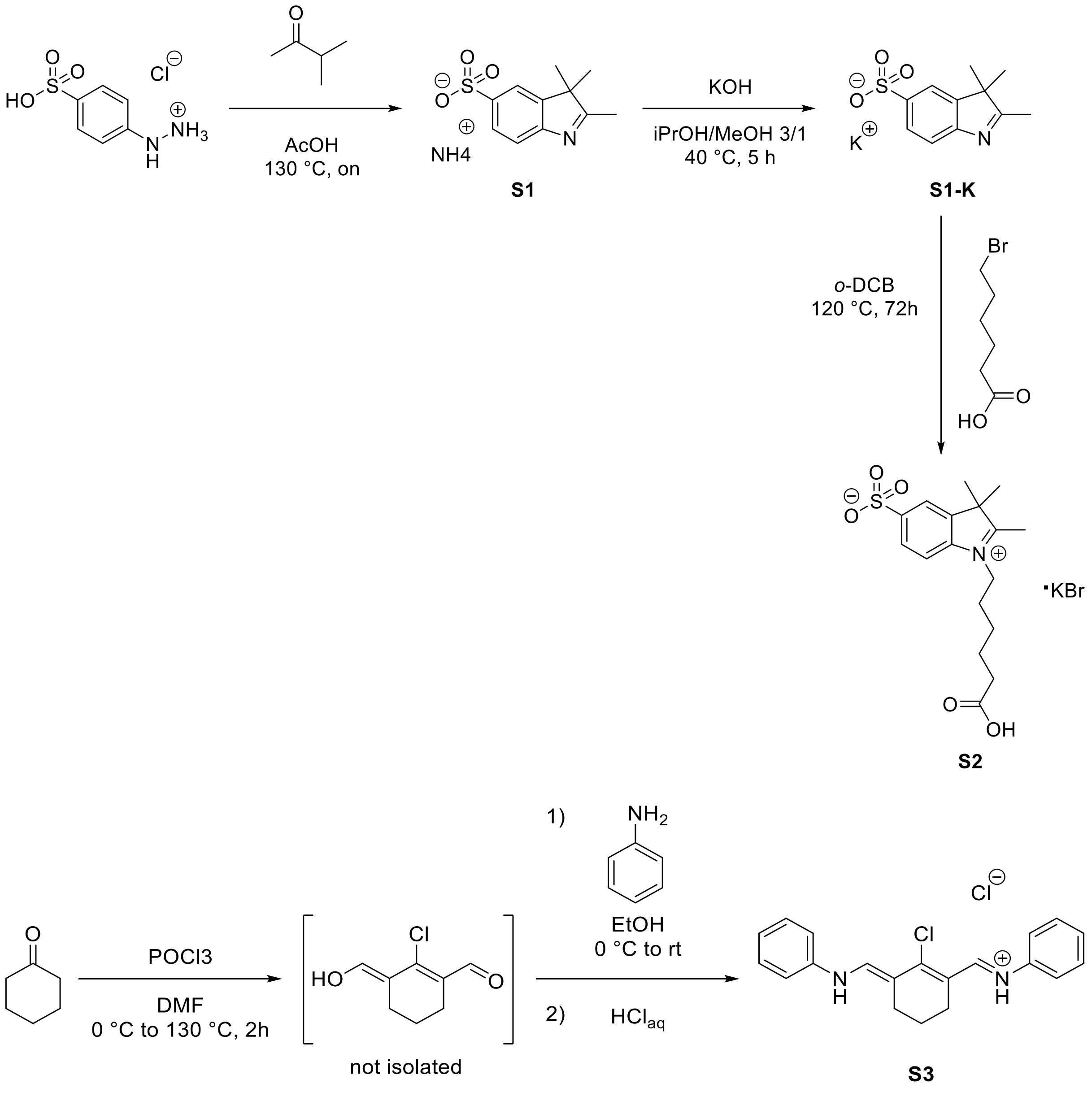

Scheme S1. Synthesis of precursors **S1**, **S2** and **S3** (*o*-DCB = *ortho*-dichlorobenzene, iPrOH = isopropyl alcohol, MeOH = methanol, AcOH = acetic acid, EtOH = ethanol, DMF = N,N-dimethylformamide).

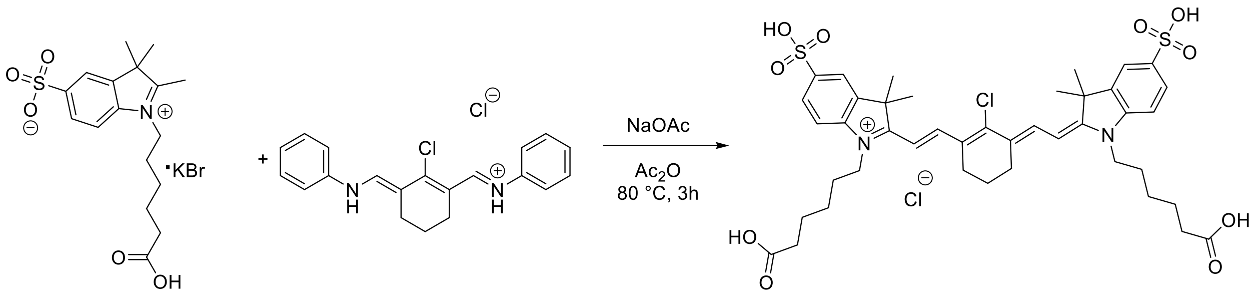

S2 **S3** **1** 19 %

Scheme S2. Synthesis of dye **1**.

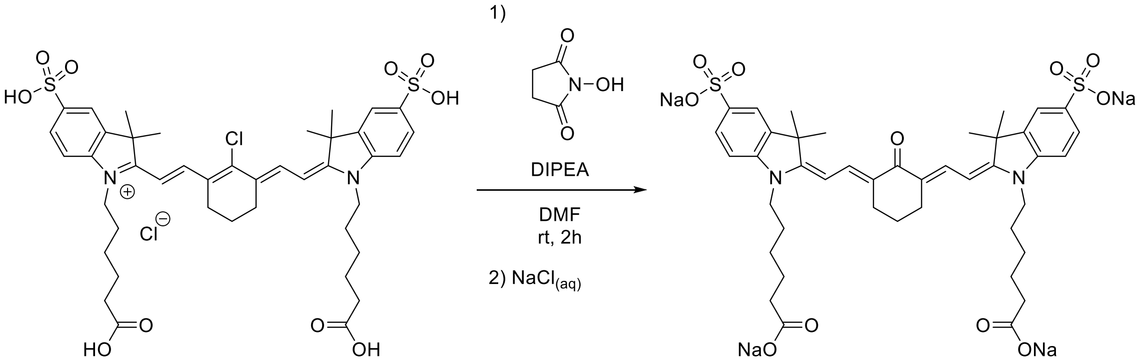

1 **2** 83 %

Scheme S3. Synthesis of dye **2** (DIPEA = diisopropyl ethyl amine).

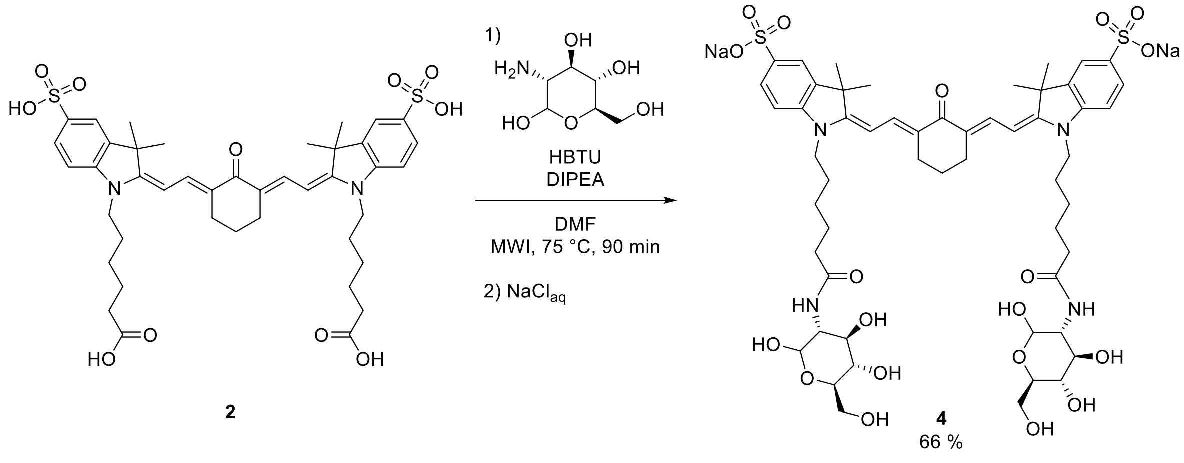

2 **4** 66 %

Scheme S4. Synthesis of dye-conjugate **4** (HBTU = 2-(1H-benzotriazol-1-yl)-1,1,3,3-tetramethyluronium hexafluorophosphate).

Synthesis of **S1**

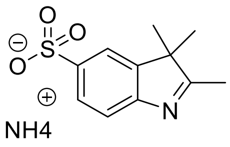

S1

A 250 mL round bottom flask was charged with 29.4 g (149 mmol) 4-hydrazinobenzenesulfonic acid hemihydrate, 17.0 mL (13.8 g, 160 mmol) 3-methyl-2-butanone and 180 mL of acetic acid. The mixture was heated to 115 °C overnight under vigorous stirring. In the course of the reaction the solids dissolve, and the initially beige suspension becomes a dark red solution. The mixture was poured into 300 mL of a 1:1 mixture of diethyl ether and ethyl acetate, and the precipitate collected by filtration. The collected solid was briefly dried. To remove traces of acetic acid left from the reaction, the solid was suspended in 200 mL of methanol, stirred vigorously for 15 minutes, and reprecipitated by addition of a 2:1 mixture of ethyl acetate and diethyl ether. The precipitate was collected again and dried in an oven at 60 °C overnight, to yield **S1** as an amorphous pink powder (22.42 g, 64 %).

^{1}H NMR (400.13 MHz, DMSO-*d*6, 298 K): δ = 7.76 (s, 1H), 7.63 (d, $^{3}J_{HH}$ = 8.0 Hz, 1H), 7.44 (d, $^{3}J_{HH}$ = 8.0 Hz, 1H), 7.11 (t, $^{1}J_{NH}$ = 51.0 Hz, 4H), 2.40 (s, 3H), 1.35 (s, 6H).

^{13}C {^{1}H} NMR (100.16 MHz, DMSO-*d*6, 298 K): δ = 192.6, 148.3, 146.5, 144.1, 125.6, 119.8, 117.1, 53.6, 22.2, 15.1.

ESI-MS – positive mode (m/z) calculated for $C_{11}H_{12}NO_3S$ [M^{+}] = 238.0545; found, 238.0545

Synthesis of **S2**

Step 1

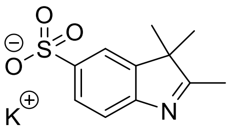

S1-K

A 250 mL round-bottom flask, equipped with a magnetic stirring bar, was charged with 7.82 g (30.5 mmol) of **S1** and 2.21 g (39.4 mmol) of potassium hydroxide. To this, 100 mL of a 1:2 mixture of methanol and isopropanol were added, and the resulting mixture was vigorously stirred and maintained at 50 °C in an open flask for 5 hours. After the first ten minutes, a color change from pink/red to orange could be observed. A litmus paper wetted with deionized water and held into the gas phase of the open flask will indicate the presence of ammonia. After six hours, and cooling to room temperature, 25 mL of diethyl ether were added, and the mixture filtered to obtain **S1-K** with the proposed structure as above, as an orange/brown powder. The orange powder is transferred to a 250 mL round-bottom flask and briefly dried *in vacuo*, before it is used in the next reaction without further purification.

Note: If this intermediate needs to be stored for prolonged amounts of time, it is recommended to store it under protective atmosphere or in a desiccator.

Step 2

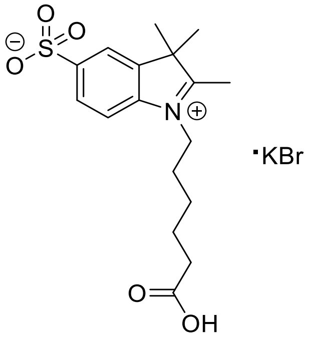

S2

To a 250 mL round-bottom flask equipped with a large magnetic stirring bar and charged with **S1-K** (ca. 8.11 g, 29.4 mmol), 15.2 g (77.9 mmol) of 6-bromohexanoic acid are added, followed by 488 mg (2.9 mmol) of potassium iodide and 80 mL of *ortho*-dichlorobenzene. The mixture is stirred vigorously and heated to 120 °C for 72 hours. After this time, the round-bottom flask is left to cool to room temperature, transferred to a fridge, and kept there overnight. The next day, the supernatant in removed by decantation, and the obtained tar is triturated with a 1:1 mixture of isopropanol and diethyl ether, repeatedly.[1] The obtained free flowing pink powder is isolated by filtration and subsequently dried in vacuo, to yield **S2** as a pink powder (7.71 g, 56 %).

^{1}H NMR (400.13 MHz, DMSO-d6, 298 K): δ = 8.02 (s, 1H), 7.92 (d, $^{3}J_{HH}$ = 8.4 Hz, 1H), 7.81 (d, $^{3}J_{HH}$ = 8.4 Hz, 1H), 4.44 (t, $^{3}J_{HH}$ = 7.7 Hz, 2H), 2.83 (s, 3H), 2.22 (t, $^{3}J_{HH}$ = 7.2, 2H), 1.89-1.79 (m, 2H), 1.59-1.48 (m, 8H), 1.46-1.36 (m, 2H).

^{13}C {^{1}H} NMR (100.16 MHz, DMSO-d6, 298 K): δ = 197.3, 174.3, 149.5, 141.5, 140.9, 126.4, 120.8, 115.0, 54.3, 47.6, 33.4, 26.9, 25.4, 24.0, 21.9, 14.0.

ESI-MS – positive mode (m/z) calculated for $C_{17}H_{24}NO_5S$ $[M]^+$ = 353.1297; found, 353.1302; deviation: 1.37 ppm.

[1] This step requires significant quantities of solvent.

Synthesis of **S3**

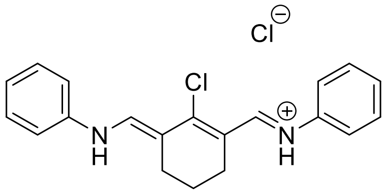

S3

Under an inert atmosphere, N,N-dimethylformamide (20 mL, 260 mmol) was added to a 250 mL three-neck flask equipped with a large magnetic stirrer. After cooling to 0 °C with an ice-bath, 16 mL of phosphoryl chloride (172 mmol) were added dropwise, and the resulting mixture was stirred and allowed to warm up to room temperature for ca. 30 min. After this time the mixture had turned dark yellow. To this, 5 mL of cyclohexanone (48 mmol) were added dropwise. After complete addition, the reaction mixture was heated to 130 °C for 2 h. During this time, it turns a dark-red color. The resulting solution was allowed to cool to room temperature, and subsequently cooled with an ice-bath. To this, 40 mL of a solution of 1:1 mixture of aniline and ethanol (221 mmol aniline) were added dropwise. Immediately, the mixture turned a dark purple, and a thick suspension formed. After complete addition of the aniline solution, the mixture is stirred for an hour and allowed to warm up to room temperature.[2] To the resulting dark mixture, 100 mL of 1 M hydrochloric acid were added, and stirring was continued for 30 minutes. The obtained suspension was filtered and the obtained solid washed with cold water, until the water runs pale yellow. Subsequently, it was washed with 100 mL of acetone and 150 mL of diethyl ether.

The product was suspended in 240 mL of a 1:1 mixture of MTBE and hexane, heated to reflux for ten minutes and allowed to cool to room temperature. Filtration afforded **S3** as an almost black, solid (14.88 g, 79 %).

^{1}H NMR (400.13 MHz, CD_3OD, 298 K): δ = 8.65 (s, 2H), 7.51-7.43 (m, 8H), 7.34-7.24 (m, 2H), 2.72 (t, $^3J_{HH}$ = 6.2 Hz, 4H), 2.00 (q, 6.2 Hz, 2H).

^{13}C {^{1}H} NMR (100.16 MHz, CD_3OD, 298 K): δ = 158.9, 150.4, 140.7, 131.1, 128.0, 119.8, 116.3, 25.6, 21.1.

ESI-MS – positive mode (m/z) calculated for $C_{20}H_{20}N_2Cl$ $[M+H]^+$ = 323.1309; found, 323.1317; deviation = 2.48 ppm

[2] Note: At this stage, the mixture may become so thick, that it will be impossible to stir with a magnetic stirring bar. In these cases, addition of some more ethanol/aniline mixture and stirring with a spatula (**careful** – residues of 'unquenched' reagents may still be present and cause rapid heating) may mobilize it. If possible, the use of a mechanical stirrer is advised for larger scale reactions.

Optimized Method for the Synthesis of **1**

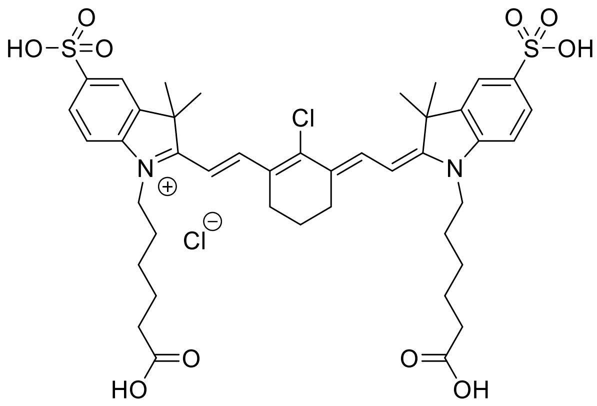

A 100 mL round-bottom flask was charged with **S2** (4.06 g, 8.58 mmol), **S3** (1.32 g, 3.69 mmol), sodium acetate (2.13 g, 21.3 mmol) and 40 mL of acetic anhydride. The mixture was heated to 80 °C for 3.5 hours. In this time, the color changed from brown to dark green. After cooling to room temperature, diethyl ether was added slowly to precipitate the product, which was collected by filtration and washed with a 1:1 mixture of THF and hexane (3x 40 mL). The obtained solid is taken up in 0.2 M KOH_{aq}, to give a brown suspension, which was stirred for 30 minutes. To this, 1 M HCl_{aq} was added dropwise, until a precipitate formed. Filtration afforded a dark green slurry, which was washed with more 1 M hydrochloric acid, before it was taken up again in acetone and precipitated by addition of diethyl ether. The dark-green precipitate was collected by filtration and dried in vacuo. Compound **1** was obtained as a dark-green solid (1.35 g, 18 %).

^{1}H NMR (400.13 MHz, DMSO-d_6, 298 K): δ = 8.25 (d, $^3J_{HH}$ = 14.0 Hz, 2H), 7.81 (d, $^4J_{HH}$ = 1.6 Hz, 2H), 7.67 (dd, J_{HH} = 8.2, 1.6 Hz, 2H), 7.39 (d, $^3J_{HH}$ = 8.2 Hz, 2H), 6.33 (d, $^3J_{HH}$ = 14.0 Hz, 2H), 4.21 (t, $^3J_{HH}$ = 7.5 Hz, 4H), 2.70 (d, $^3J_{HH}$ = 6.3 Hz, 4H), 2.20 (t, $^3J_{HH}$ = 7.2 Hz, 4H), 1.95 – 1.78 (m, 2H), 1.78 – 1.69 (m, 4H), 1.67 (s, 12H), 1.60 – 1.48 (m, 4H), 1.47 – 1.33 (m, 4H).

^{13}C {^{1}H} NMR (100.16 MHz, DMSO-d_6, 298 K): δ = 174.8, 172.9, 148.5, 146.0, 143.4, 142.6, 141.0, 127.1, 126.7, 120.4, 111.2, 102.5, 49.5, 44.3, 34.0, 27.9, 27.2, 26.3, 26.1, 24.7, 20.8.

ESI-MS – negative mode [m/z] calculated for $C_{42}H_{51}ClN_2O_{10}S_2$ $[M-H]^-$ = 841.2601; found, 841.2579; deviation = 2.62 ppm. Calculated for $C_{42}H_{50}ClN_2O_{10}S_2$ $[M-2H]^{2-}$ = 420.1264; found, 420.1250; deviation = 3.33 ppm

UV/Vis (PBS): $\lambda_{max(Abs)}$ [nm] (ε [$M^{-1}\cdot cm^{-1}$]) = 782 (195,000)

Fluorescence Spectroscopy (PBS): $\lambda_{(Ex)}$ [nm] = 770, $\lambda_{max(Em)}$ [nm] = 803, ϕ_F = 0.045 Ref: ICG)

Analytical HPLC: Hamilton C_{18}-silica, Method A, t_R = 7.49 min, Method B, t_R = 8.37 min (98 % at 780 nm)

Synthesis of **2**

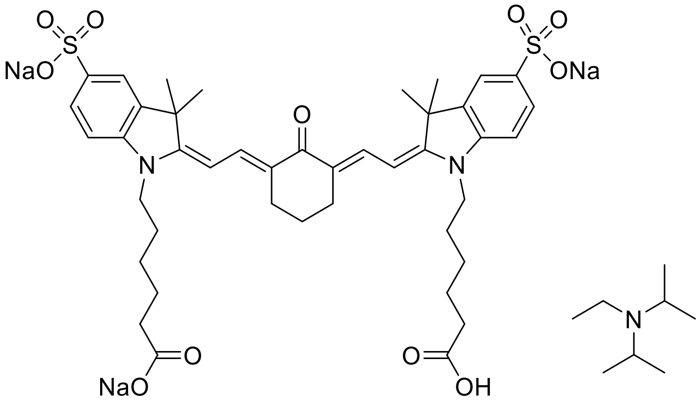

isolated as a mixed salt with 0.8 equiv. DIPEA

In a 25 mL round-bottom flask, dye **1** (87 mg, 99 µmol) was dissolved in 4 mL of peptide-grade DMF. To this, 83 µL (ca. 495 µmol) of DIPEA and 68 mg (594 µmol) of N-hydroxy succinimide were added. The resulting mixture was stirred for 1 h at room temperature, before it was poured into 25 mL of deionized water. The obtained aqueous solution was loaded onto a reverse-phase flash chromatography cartridge and washed with a 3 M solution of sodium chloride in water, before the product was eluted using a water/acetonitrile gradient. The obtained product was concentrated under reduced pressure and finally lyophilized, to yield **2** as a red powdery solid (56 mg, 65 %).

^{1}H NMR (400.13 MHz, CD_3OD, 298 K): δ = 8.14 (d, $^3J_{HH}$ = 12.8 Hz, 2H), 7.72 (dd, J_{HH} = 8.3, 1.7 Hz, 2H), 7.70 (d, $^4J_{HH}$ = 1.7 Hz, 2H), 6.89 (d, $^3J_{HH}$ = 8.3 Hz, 2H), 5.65 (d, $^3J_{HH}$ = 13.2 Hz, 2H), 3.81 (t, $^3J_{HH}$ = 7.6 Hz, 4H), 2.67-2.58 (m, 4H), 2.21 (t, $^3J_{HH}$ = 7.5 Hz, 4H), 1.91-1.84 (m, 2H), 1.78-1.65 (m, 20H), 1.51-1.44 (m, 4H). (DIPEA signals not listed here).

^{13}C {^{1}H} NMR (100.16 MHz, CD_3OD, 298 K, chemical shifts determined by HSQC and HMBC): δ = 164.6, 140.6, 139.9, 135.5, 127.5, 120.6, 107.7, 94.9, 47.9, 43.5, 38.2, 28.9, 27.9, 27.0, 26.6, 23.7. (DIPEA signals not listed here, some signals could not be assigned from the correlation spectra).

ESI-MS – positive mode (m/z) calculated for $C_{42}H_{50}N_2O_{11}S_2$ $[M - 2H]^{2-}$ = 411.1433; found, 411.1413; deviation = 4.86 ppm.

UV/Vis (PBS): λ_{maxAbs} [nm] (ε [$M^{-1}\cdot cm^{-1}$]) = 563 (approx. 58,000).

Fluorescence (PBS): λ_{Exc} [nm] = 540, λ_{maxEm} [nm] = 641, ϕ_F = 0.221 (reference: Rhodamine B).

Analytical HPLC: Hamilton C_{18}, Method A, t_R = 7.79 min (99 % at 560 nm).

Synthesis of **3**

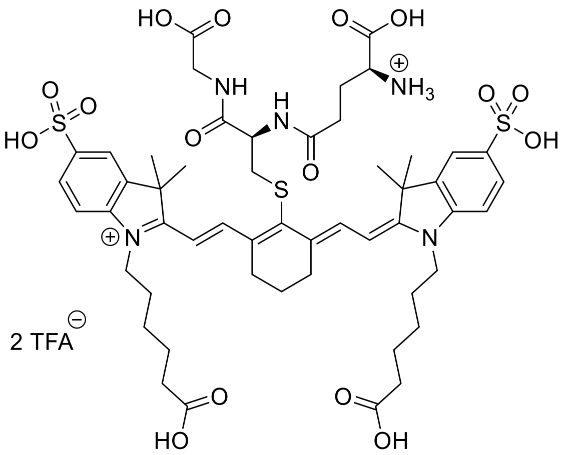

A 7 mL vial was charged with 25 mg (29.6 µmol) of **1**, 4 mL of PBS and 25 mg (81 µmol) of reduced glutathione. The resulting mixture was stirred for 4 h at room temperature. The dark green solution was subsequently loaded onto a reverse-phase chromatography column (C_{18}-silica). The product was eluted using a water/acetonitrile gradient (0 % ACN to 50 % ACN, 0.1 vol% TFA in water). After removing most of the organic solvent under reduced pressure, the remaining aqueous solution was dried by lyophilization to yield **3** as a dark-green solid (36 mg, 92 %).

^{1}H NMR (400.13 MHz, DMSO-d_6, 298 K): δ = 8.58 (d, J = 14.0 Hz, 2H), 8.50 (q, J = 7.9, 6.7 Hz, 1H), 8.33 (t, J = 6.0 Hz, 1H), 8.29-8.22 (m, 2H), 7.76 (s, 1H), 7.65 (dd, J = 8.2, 1.6 Hz, 2H), 7.35 (d, J = 8.4 Hz, 2H), 6.28 (d, J = 14.0 Hz, 2H), 4.56-4.45 (m, 1H), 4.24-4.09 (m, 4H), 3.95-3.86 (m, 1H), 3.75-3.69 (m, 2H), 3.15-3.01 (m, 4H), 2.68-2.57 (m, 4H),2.40-2.29 (m, 3H), 2.20 (t, $^3J_{HH}$ = 7.2 Hz, 4H), 2.03-1.92 (m, 2H), 1.90 – 1.78 (m, 2H), 1.77 – 1.49 (m, 20H).

ESI-MS – negative mode [m/z] calculated for $C_{52}H_{67}N_5O_{16}S_3$ $[M-H]^-$ = 1113.3745; found, 1113.3700; deviation = 4.04 ppm. Calculated for $C_{52}H_{66}N_5O_{16}S_3$ $[M-3 H]^{2-}$ = 555.6800; found, 555.6806; deviation = 1.08 ppm

UV/Vis (PBS): $\lambda_{max(Abs)}$ [nm] (ε [$M^{-1}\cdot cm^{-1}$]) = 793 (198,000)

Fluorescence Spectroscopy (PBS): λ_{Exc} [nm] = 740 , λ_{maxEm} [nm] = , ϕ_F = 0.041 (reference ICG)

Analytical HPLC: Hamilton C_{18}-silica, Method B, t_R = 7.62 min (96 % at 780 nm).

Synthesis of **4**

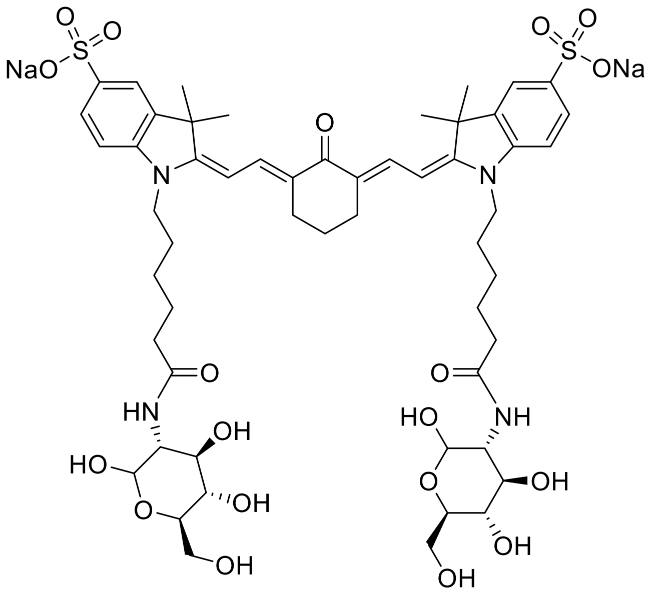

In a microwave vial, **2** (12 mg, 14 µmol) was dissolved in 2.5 mL of DMF. To this, HBTU (30 mg, 79 µmol) was added, followed by DIPEA (23 µL, ca. 140 µmoL) and glucosamine hydrochloride (20 mg, 92 µmol). The mixture was heated to 75 °C under microwave irradiation for 90 minutes. After cooling to room temperature, the mixture was poured into 20 mL of deionized water. The solution was loaded on a reverse-phase flash chromatography column and eluted with a water/MeOH gradient (10 mM ammonium formate buffer in mobile phases). The obtained product fraction was concentrated under reduced pressure, to remove excess methanol. The obtained aqueous solution was loaded onto a reverse-phase flash chromatography cartridge and washed with a 3 M solution of sodium chloride in water, before the product was eluted with unbuffered methanol. The obtained product was concentrated under reduced pressure and finally lyophilized, to yield **4** as a red solid (11 mg, 66 %).

^{1}H NMR (400.13 MHz, CD_3OD, 298 K): δ = 8.14 (d, $^3J_{HH}$ = 13.2 Hz, 2H), 7.74-7.70 (m, 4H), 6.91-6.86 (m, 2H), 5.65 (d, $^3J_{HH}$ = 13.3 Hz, 2H), 3.87-3.76 (m, 8H), 3.72-3.67 (m, 2H) 2.64-2.59 (m, 4H), 2.31-2.18 (m, 8H), 1.90-1.84 (m, 2H), 1.76-1.65 (m, 24H), 1.48-1.42 (m, 4H).

ESI-MS – negative mode (m/z) calculated for $C_{54}H_{72}N_4O_{19}S_2$ $[M - 2H]^{2-}$ = 572.2122; found, 572.2095; deviation = 4.72 ppm.

UV/Vis (PBS): $\lambda_{max(Abs)}$ [nm] = 563 (approx.. 58,000).

Fluorescence (PBS): $\lambda_{(Ex)}$ [nm] = 540, $\lambda_{max(Em)}$ [nm] = 641.

Analytical HPLC: Hamilton C_{18}, Method A, t_R = 6.83 min (98 % at 560 nm)

Synthesis of **5**

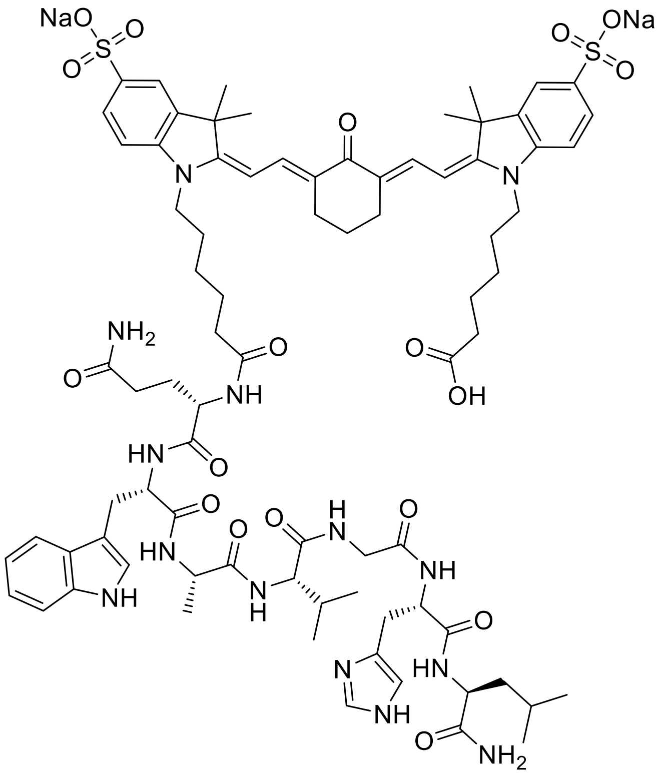

In a microwave vial, **2** (6 mg, 6.8 µmol) was dissolved in 1.5 mL of DMF. To this, HBTU (3 mg, 7.9 µmol) was added, followed by DIPEA (3 µL, 17 µmol) and bombesin [7-13] (5 mg, 5.4 µmol). The mixture was heated to 50 °C under microwave irradiation for 90 minutes. After cooling to room temperature, the mixture was poured into 20 mL of deionized water and filtered over a pad of C_{18}-silica, which was washed with a 1:1 mixture of acetonitrile and water to elute trapped product. The resulting solution was loaded on a reverse-phase flash chromatography column and eluted with a water/MeOH gradient (10 mM ammonium formate buffer in mobile phases). The obtained product fraction was concentrated under reduced pressure, to remove excess methanol. The obtained aqueous solution was loaded onto a reverse-phase flash chromatography cartridge and washed with a 1 M solution of sodium chloride in water, before the product was eluted with a gradient of deionized water and methanol (0 % MeOH to 75 % MeOH). The obtained product was concentrated under reduced pressure and finally lyophilized, to yield **5** as a red solid (3.7 mg). Due to the limited quantity of product, only limited analytical work was possible.

ESI-MS – negative mode (m/z) calculated for $C_{80}H_{104}N_{14}O_{18}S_2$ $[M - 2H]^{2-}$ = 806.3553; found, 806.3515; deviation = 4.71 ppm.

Analytical HPLC: Hamilton C_{18}, Method A, t_R = 8.29 min (purity 91-95 % at 560 nm).

Synthetic protocol for L-Bombesin [7-13], QWAVGHL

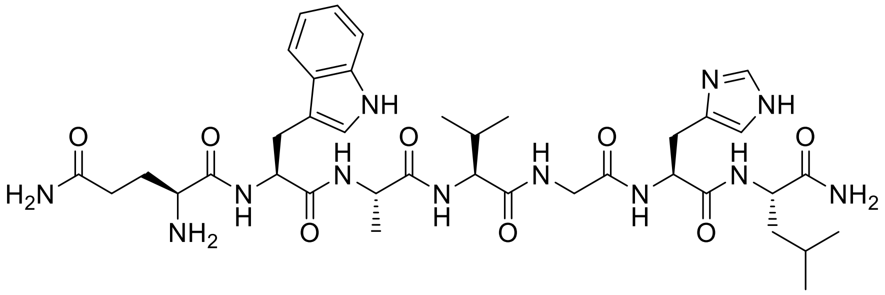

The bombesin [7-13] fragment used in this study was synthesized by solid phase peptide synthesis on a Rink amide resin using an adaptation from our previously established method [21]. To this end, stock solutions with a concentration of 0.5 M of the respective protected amino acid were prepared. Throughout the synthesis, three equivalents of amino acids were used.

Initially, the resin was swelled in peptide grade DMF, before the first amino acid derivative, Fmoc-leucine, was coupled using a mixture of HBTU in DMF, HOBt in DMF and DIPEA in NMP. Couplings were generally conducted at 75 °C for 10 minutes, unless otherwise mentioned. Subsequently, a washing step with DMF was performed, before the Fmoc protecting group was removed using a solution of 20 % piperidine in DMF (twice). Following this, the resin was washed two more times with DMF. Subsequent couplings of Fmoc-His(Trt), Fmoc-Gly, Fmoc-Val, Fmoc-Ala, Fmoc-Trp(Boc) and Fmoc-Gln(Trt) followed the same general procedure, except for valine, which was allowed to react for 20 minutes, and tryptophane, which was allowed to react for 90 minutes at room temperature. After the last coupling step was completed, and the glutamine residue deprotected, the resin was washed twice with DMF and twice with dichloromethane, before the peptide was cleaved and remaining protecting groups removed by addition of an 88:5:5:2 mixture of TFA, phenol, triisopropylsilane and water for 3 hours. After this time, the solution was added drop-wise to cold diethyl-ether, resulting in a voluminous colorless precipitate which was centrifuged to a pellet. The supernatant was removed and the resulting solid resuspended and centrifuged in cold diethyl ether. This procedure was repeated twice, before the crude product was purified by reverse-phase flash chromatography over C18-silica (H2O/ACN 95/5 to 50/50, with 0.1 % TFA in both solvents). Combined product fractions were frozen and lyophilized to yield [7-13] bombesin as a colorless solid in 63 % yield.

ESI-MS – positive mode (m/z) calculated for $C_{38}H_{58}N_{12}O_8$ $[M+H]^+$ = 405.2245; found, 405.2251; deviation = 1.48 ppm.

Analytical HPLC: Hamilton C_{18}, Method B, t_R = 7.24 min (83 % at 254 nm).

MHI-148 and its Derived Keto-Polymethine

MHI-148 was synthesized according to a literature procedure and reacted with N-hydroxy succinimide analogous to the synthesis of **1** (**Scheme S5**).[1, 2]

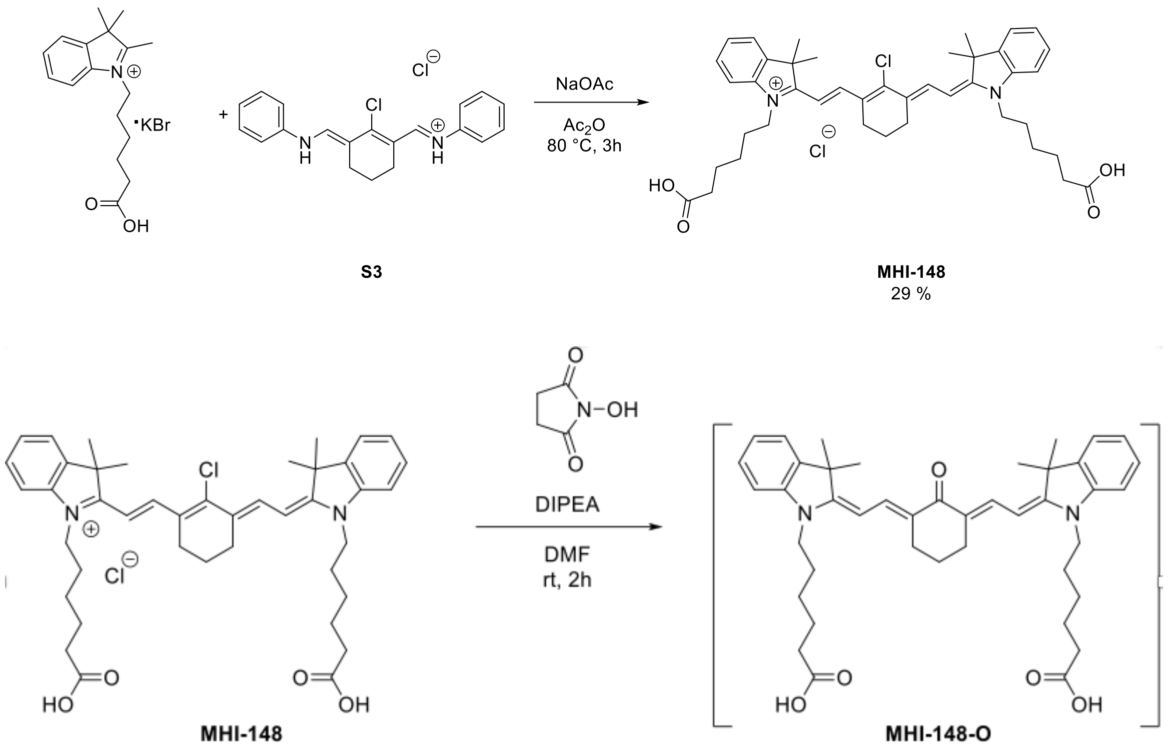

Scheme S5. Synthesis of known **MHI-148** and of the corresponding keto-polymethine denoted **MHI-148-O**.

Stepwise synthesis of MHI-148

Step 1: Compound S-I

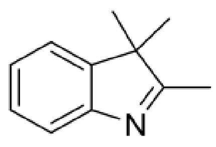

A 500 mL round bottom flask was charged with 14.92 g (103 mmol) phenylhydrazine hydrochloride, 13.7 mL (11.1 g, 128 mmol) 3-methyl-2-butanone and 150 mL of acetic acid. The mixture was heated to 100 °C under vigorous stirring for three hours. During this time, the

solids dissolve, and the initially beige suspension becomes a dark brown solution. The mixture was poured into 300 mL of water. To this, 40 grams of sodium hydroxide are added portion-wise. Upon neutralization, a dark-red oil starts to form on top of the aqueous mixture. The mixture was transferred to an extraction funnel and extracted with dichloromethane (4 x 75 mL). Collected organic phases were washed with a saturated solution of sodium hydrogen carbonate (2 x 50 mL) and brine (1 x 75 mL). Subsequently, the organic phase was dried over magnesium sulfate and concentrated under reduced pressure. The resulting oil was further purified by column chromatography (n-hexane/EtOAc 19:1), and the combined product fractions were concentrated under reduced pressure to yield compound S-I as an orange/red oil (14.48 g, 82 mmol, 80 %).

^{1}H NMR (400.13 MHz, $CDCl_3$, 298 K): δ = 7.49 (d, $^{3}J_{HH}$ = 7.6 Hz, 1H), 7.29-7.21 (m, 2H), 7.16 (dt, J_{HH} = 7.4 Hz, 1.1 Hz), 2.24 (s, 3H), 1.26 (s, 6H).

^{13}C {^{1}H} NMR (100.16 MHz, $CDCl_3$, 298 K): δ = 188.0, 153.8, 145.7, 127.6, 125.1, 121.4, 120.0, 53.7, 23.2, 15.5.

ESI-MS – positive mode (m/z) calculated for $C_{11}H_{13}N$ $[M]^+$ = 159.1048; found, 159.1052; deviation = 2.51 ppm.

Step 2. Compound S-II

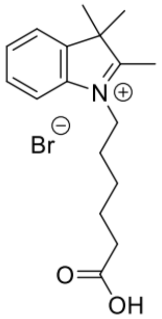

A 500 mL round-bottom flask equipped with a large magnetic stirring bar was charged with 6.32 g of **S-I** (35.7 mmol), 120 mL of acetonitrile and 12.55 g (64 mmol) of 6-bromohexanoic acid. The mixture was stirred vigorously and heated to reflux overnight. After cooling to room temperature, the solvent was removed under reduced pressure. The flask was placed in an ice bath and the residue dissolved in the smallest possible amount of dichloromethane (ca. 100 mL). To this, 350 mL of diethyl ether were added dropwise to precipitate the desired product. The obtained solid was isolated filtration and washed with a 2:1 mixture of diethyl ether and hexane to yield **S-II** as a beige-coloured solid (4.27 g, 30 %).

^{1}H NMR (400.13 MHz, DMSO-d6, 298 K): δ = 8.03-7.94 (m, 1H), 7.88-7.81 (m, 1H), 7.66-7.57 (m, 2H), 4.46 (t, $^{3}J_{HH}$ = 7.8 Hz, 2H), 2.85 (s, 3H), 2.23 (t, $^{3}J_{HH}$ = 7.2, 2H), 1.84 (p, $^{3}J_{HH}$ = 8.2, 7.8 Hz, 2H), 1.62-1.48 (m, 8H), 1.47-1.38 (m, 2H).

^{13}C {^{1}H} NMR (100.16 MHz, DMSO-d6, 298 K): δ = 196.5, 174.3, 141.9, 141.1, 129.4, 128.9, 123.5, 115.5, 54.2, 47.4, 33.4, 26.9, 25.4, 24.0, 22.0, 14.0.

ESI-MS – positive mode (m/z) calculated for $C_{17}H_{24}NO_2$ $[M]^+$ = 274.1808; found, 274.1807; deviation = 0.36 ppm.

Step 3. MHI-148

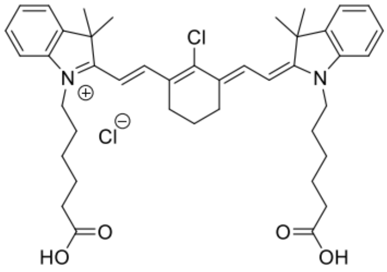

A 250 mL round-bottom flask was charged with **S-I** (3.01 g, 10.98 mmol), **S-II** (1.94 g, 5.49 mmol), sodium acetate (0.55 g, 5.49 mmol) and 100 mL of ethanol. The mixture was heated to 80 °C for 6 hours. In this time, the color changed from brown to dark blue. After cooling to room temperature, the solvent was removed under reduced pressure, to give an almost black viscous mass. The mixture is taken up in dichloromethane, loaded onto a silica column and slowly eluted using dichloromethane/methanol (19/1 to 9/1 DCM/MeOH). The dark green product fractions are combined and concentrated under reduced pressure to give **MHI-148** as a dark green, almost black solid (1.14 g, 29 %).

^{1}H NMR (400.13 MHz, DMSO-d_6, 298 K): δ = 8.25 (d, $^3J_{HH}$ = 14.1 Hz, 2H), 7.63 (d, $^3J_{HH}$ = 7.4 Hz, 2H), 7.48-7.38 (m, 4H), 7.28 (t, $^3J_{HH}$ = 7.2 Hz, 2H), 6.33 (d, $^3J_{HH}$ = 14.1 Hz, 2H), 4.27-4.15 (m, 4H), 2.77-2.64 (m, 4H), 2.21 (t, $^3J_{HH}$ = 7.3 Hz, 4H), 1.91 – 1.82 (m, 2H), 1.79 – 1.60 (m, 16H), 1.67 (s, 12H), 1.59 – 1.52 (m, 4H), 1.46 – 1.35 (m, 4H).

^{13}C {^{1}H} NMR (100.16 MHz, DMSO-d_6, 298 K): δ = 174.3, 172.2, 148.0, 143.0, 142.1, 141.1, 128.6, 126.2, 125.2, 122.5, 111.5, 101.6, 49.0, 48.6, 33.5, 27.5, 26.7, 25.8, 25.7, 24.2, 20.4.

ESI-MS – negative mode [m/z] calculated for $C_{42}H_{52}ClN_2O_4$ $[M]^+$ = 683.3615; found, 683.3621; deviation = 0.87 ppm.

UV/Vis (PBS): λ_{maxAbs} [nm] (ε [M^{-1}•cm^{-1}]) = 775 (185,000)

Fluorescence Spectroscopy (PBS): λ_{Exc} [nm] = 770, λ_{maxEm} [nm] = 803, ϕ_F = 0.016 (reference: ICG)

Treatment of MHI-148 with standard peptide coupling agents

Briefly, in a 25 mL round-bottom flask, MHI-148 (34 mg) was dissolved in 4 mL of peptide-grade DMF. To this, 5 equivalents of DIPEA and 6 equivalents of N-hydroxy succinimide were added. The resulting mixture was stirred for 1 h at room temperature, before it was diluted with ethyl acetate (100 mL). This as-obtained solution was filtered over silica, washed with dichloromethane and subsequently eluted with a 1 to 3 mixture of methanol and dichloromethane. Fractions were monitored by mass spectrometry. The as-obtained product fraction (on several mgs scale) was concentrated under reduced pressure, to yield the oxo-derivatized MHI-148 (denoted MHI-148-O in this work) as an oily product, which solidified after several days. To purify the desired merocyanine derivative, silica column chromatography with a dichloromethane/methanol gradient (0-10 % MeOH) was performed several times. While methanol is somewhat undesirable, due to its tendency to form methyl esters under these conditions, no alternative could be found. Additional work-up steps had to be included to obtain this oxo-form of the **MHI-148** dye denoted **MHI-148-O** in by ESI HR MS only, as the low water-solubility of this product hampered the semiPrep HPLC separation.

The change in the UV/Vis spectrum of MHI-148 upon treatment with HOBt in the presence of DIPEA and the ESI-MS of the resulting keto-polymethine is depicted below (**Figure S1**).

ESI-MS – negative mode (m/z) calculated for $C_{42}H_{51}N_2O_5$ $[M - H]^{2-}$ = 663.3803; found, 663.3776; deviation = 4.07 ppm.

UV/Vis (MeOH): λ_{maxAbs} [nm] = 533. Fluorescence (MeOH): λ_{Exc} [nm] = 500, λmaxEm [nm] = 624.

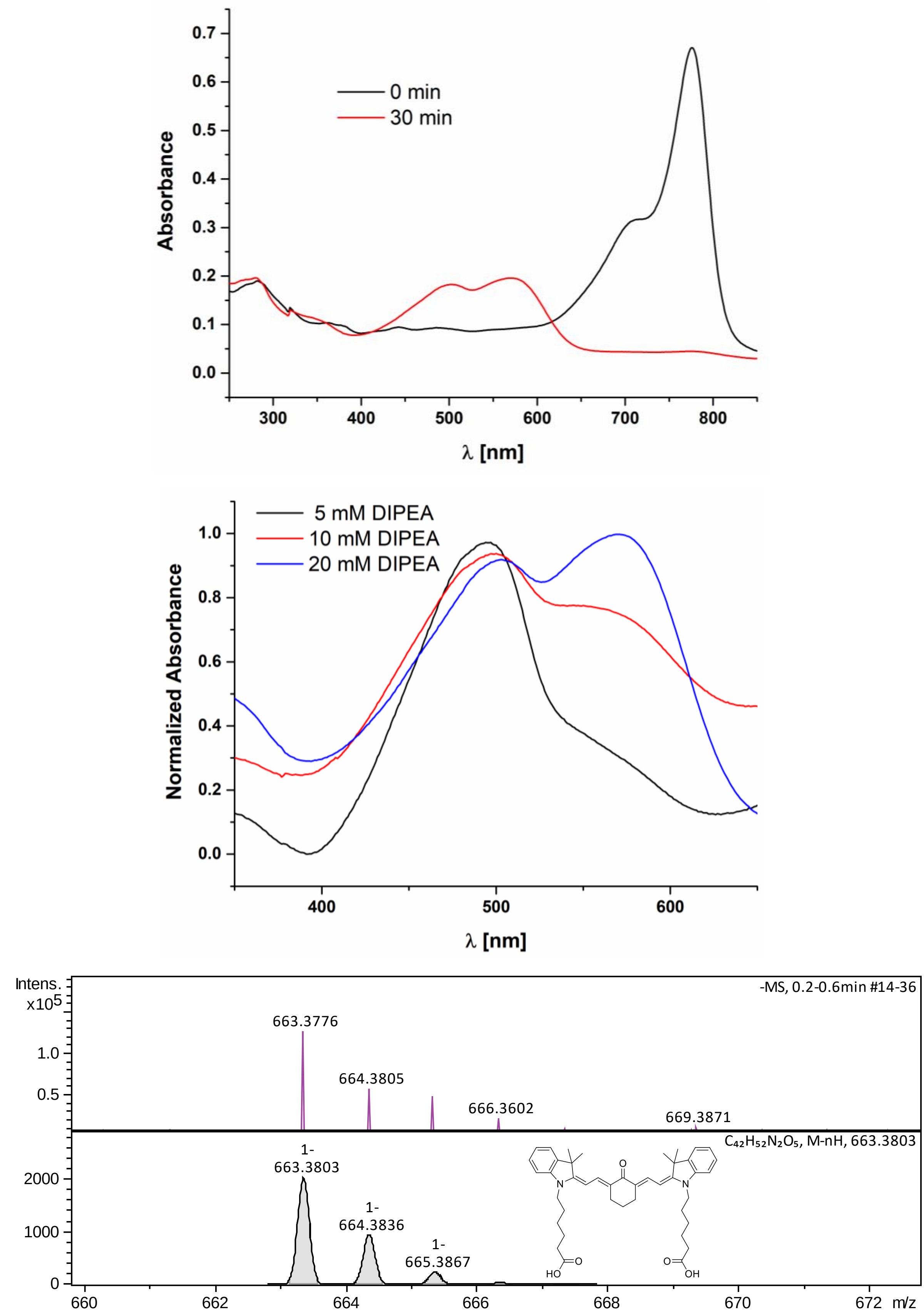

Figure S1. Top: Change of electronic spectra of **MHI-148** in the presence of HOBt (3.6 mM **MHI-148**, 7.2 mM HOBt, 20 mM DIPEA in DMF). Middle: Change of electronic spectra of **MHI-148** in the presence of HOBt at various concentrations of DIPEA (3.6 mM MHI-148, 7.2 mM HOBt, concentration of DIPEA indicated). Below: Detail of the ESI-MS (neg. mode) of the product obtained from the reaction between **MHI-148** and HOBt and its assignment to **MHI-148-O**.

(a) Pos. ESI-MS showing isotope pattern corresponding to an HOBt – MHI-148 adduct

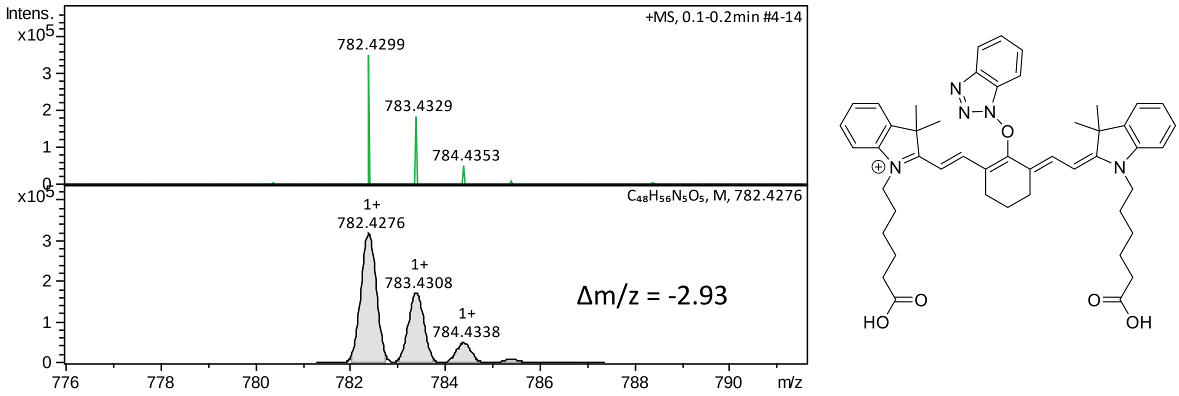

(b) Neg. ESI-MS showing isotope pattern corresponding to the formula of a postulated decomposition product (structure postulated)

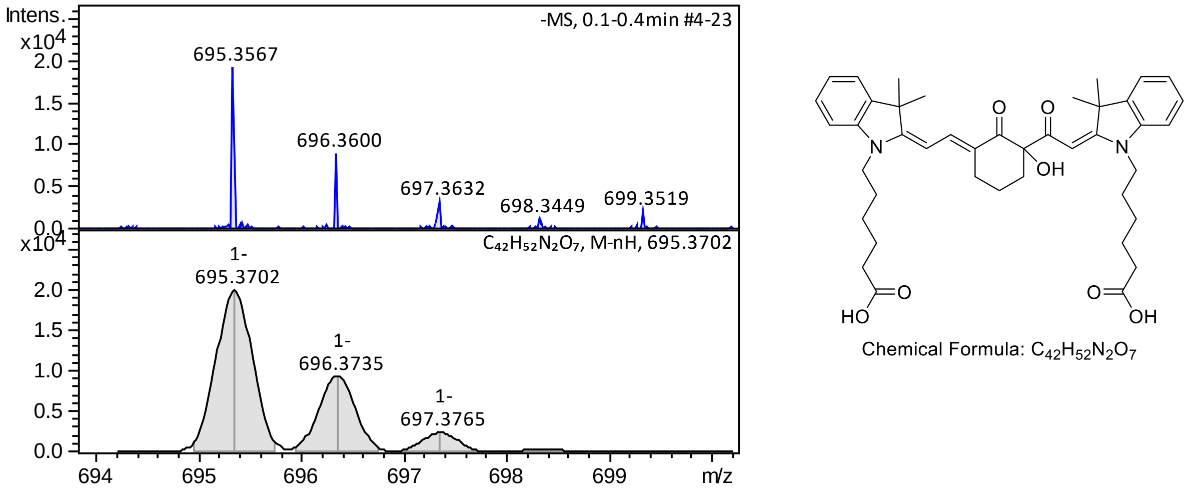

(c) Pos. ESI-MS showing isotope pattern corresponding to the formula of a postulated decomposition product (structure postulated):

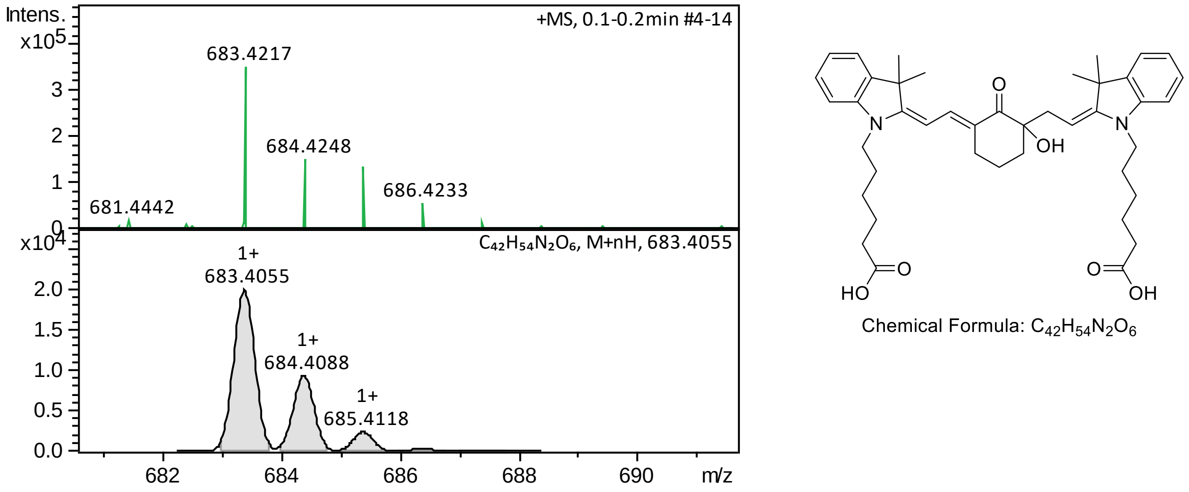

Figure S2. Mass Spectrometry of **MHI-148-O** was performed under a variety of conditions.

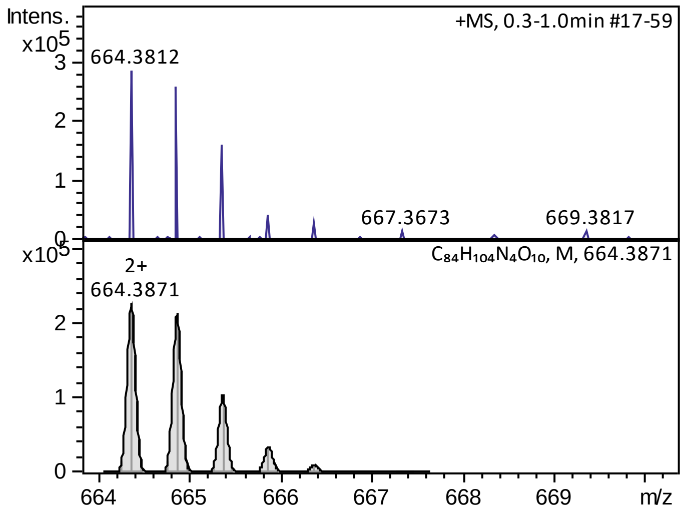

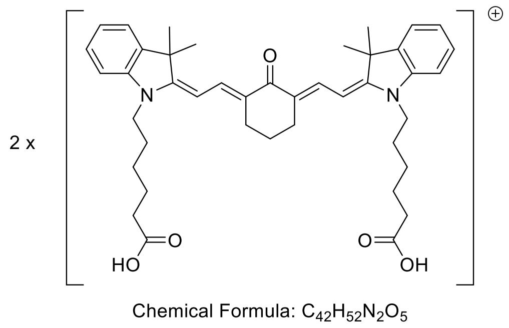

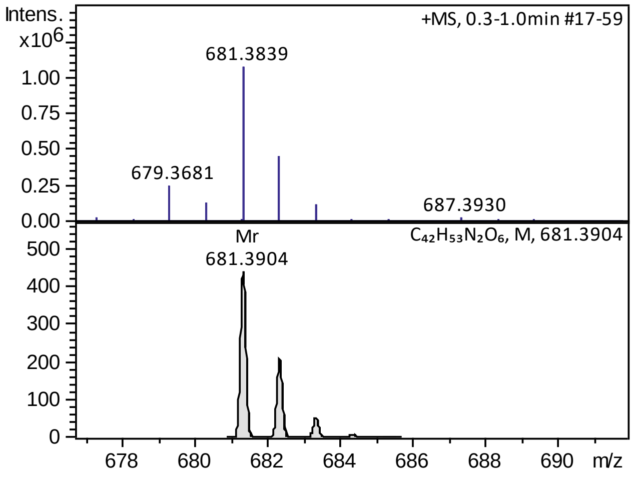

Figure S3: **P**os. ESI-MS of MHI-148-O acidified with 0.1 vol% formic acid

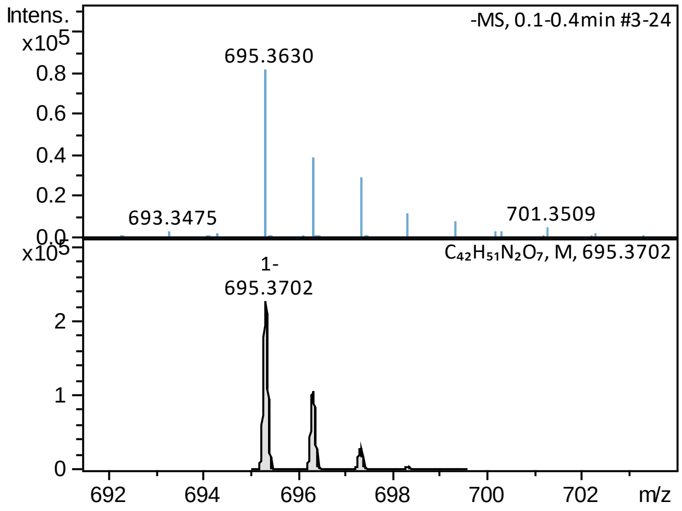

Figure S4. Corresponding Neg. ESI-MS for the **MHI-148-O** sample acidified with 0.1 vol% formic acid

Neg. ESI-MS, crude:

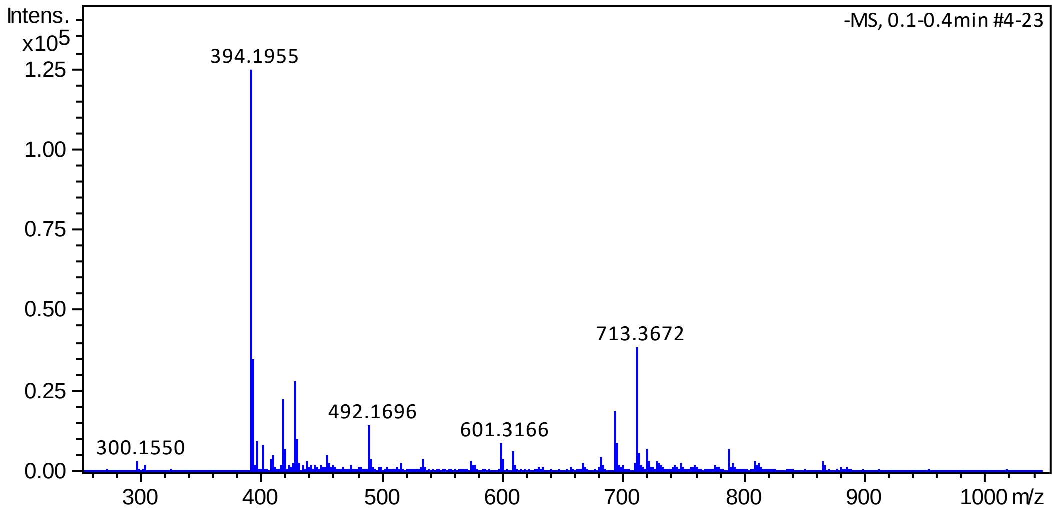

Pos. ESI-MS (sample dissolved in water/DMF 95/5), directly from reaction mixture:

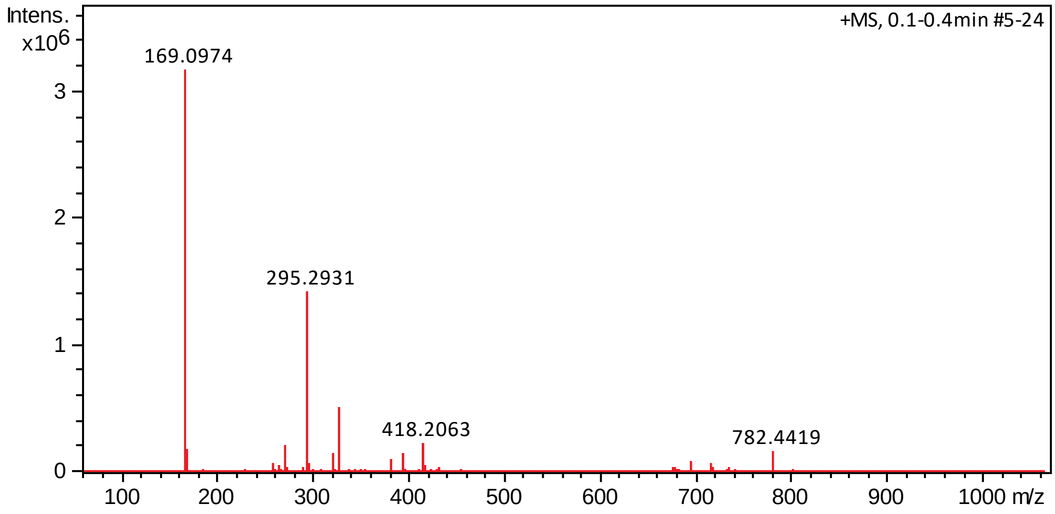

Pos. ESI-MS (sample dissolved in water, acidified with 0.1 vol% formic acid)

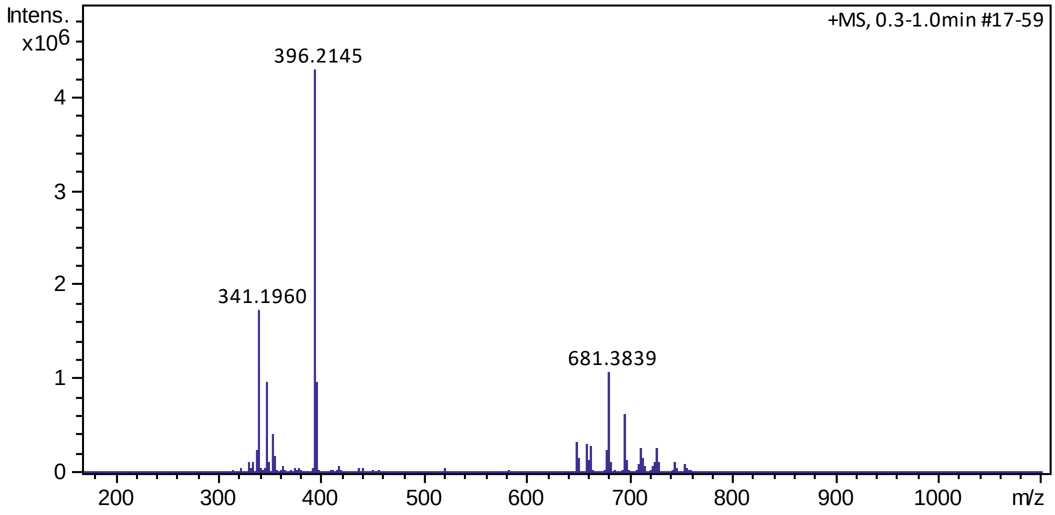

Figure S5. Typical full mass spectra for MHI-148-O

Attempted Peptide Bond Formations Reactions of 1

To test whether the formation of conjugates would give the desired compounds in higher yields without using N-hydroxy based additives, a test reaction was conducted, according to **Scheme S6**. The chromatogram obtained by flash chromatography (**Figure S2**) of the crude product suggested the presence of more than the three expected products. Samples of each fraction were taken and characterized by HRMS. Although matrix effects made assigning difficult, some signals corresponded to molecular formulae which agreed with the proposed N-acylureas (**Figure S3**). It is well-known, that the formation of N-acylureas from carbodiimide-based coupling reagents is a common side-reaction.[3]

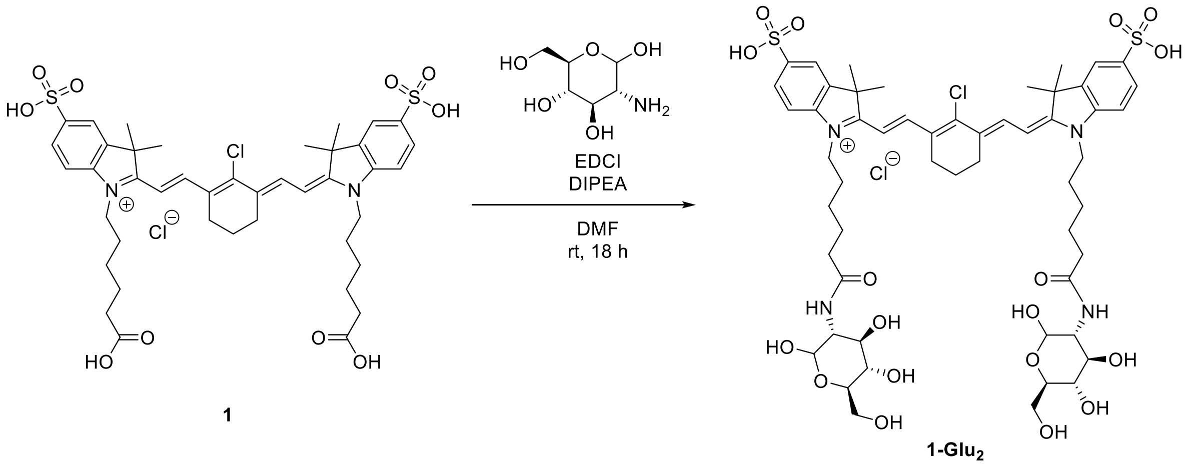

Scheme S6. Attempted synthesis of glucosamine-conjugate **1-Glu₂** (Proposed compound **6)** using only EDCI for coupling.

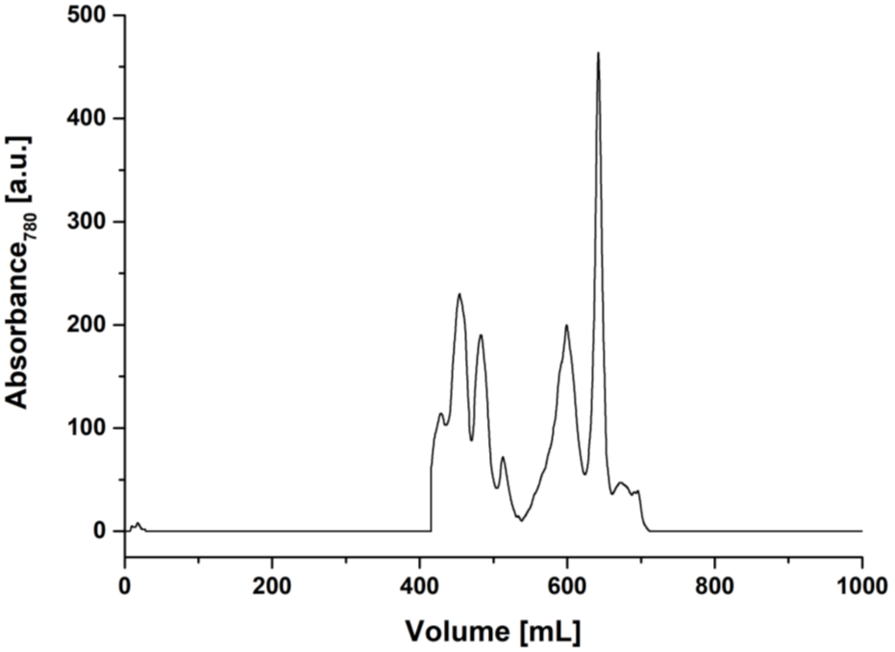

Figure S6. Chromatogram of reverse-phase flash chromatography profile (780 nm) of the attempted synthesis of **1-Glu2 (6)** conjugate, prior to separation and fractions characterization by ESI MS.

.

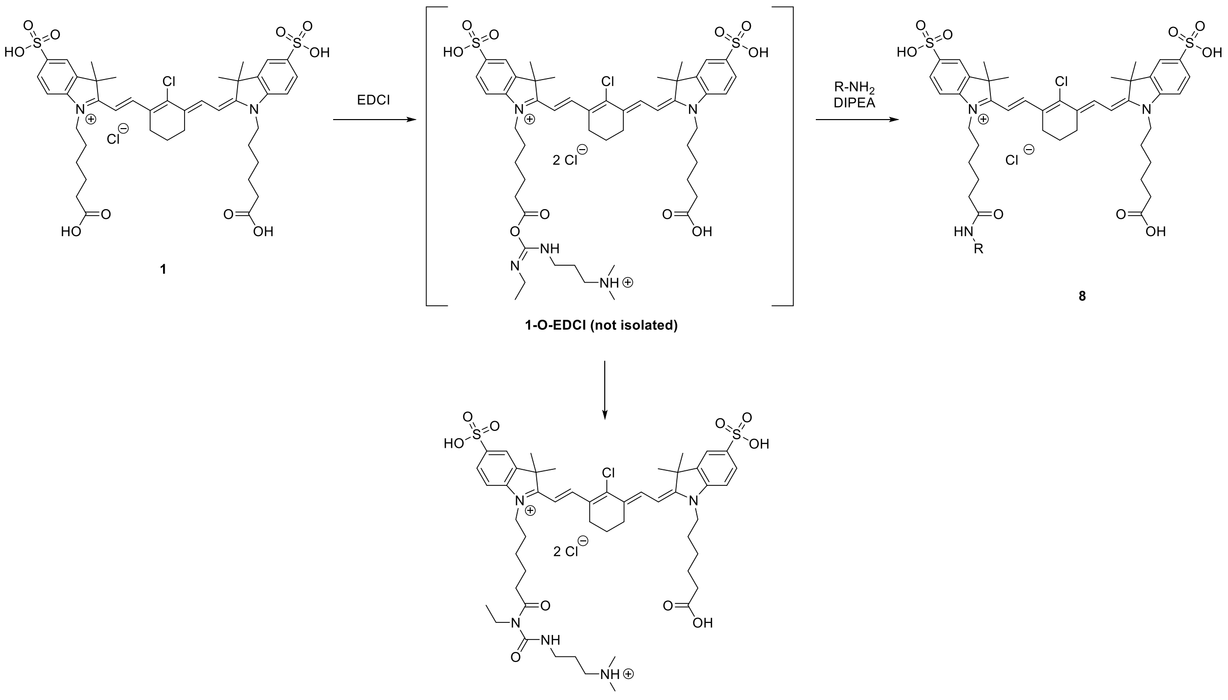

1-N-EDCI

(Proposed Compound 7)

Scheme S7. Generalized schematic synthesis of conjugates of Compound **1** with the structure of a proposed side-products (e.g. **1-N-EDCI**, Compound **7,** and for RNH_2 = D-glucosamine, proposed Compound **8**) assigned by HR MS as indicated below.

.

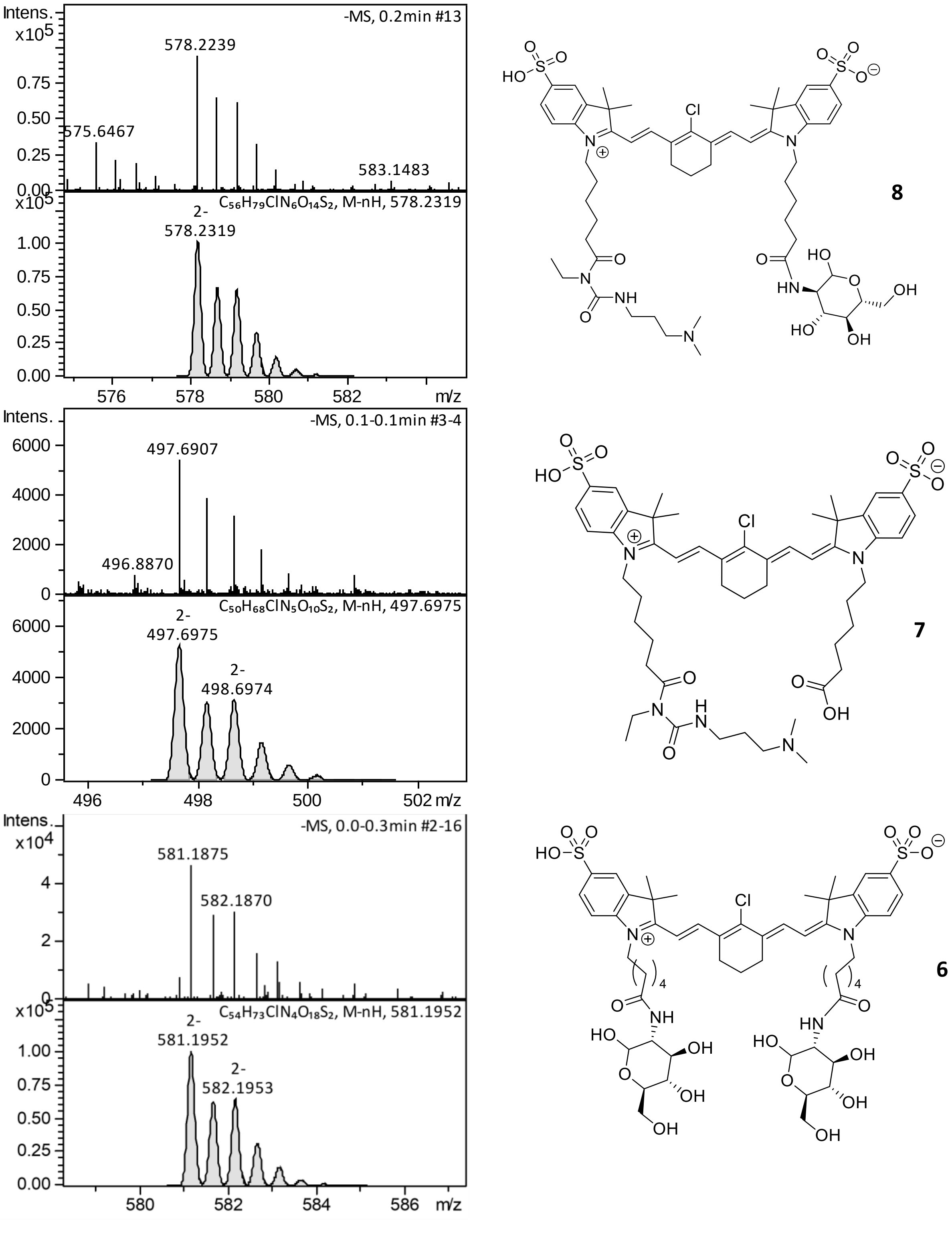

Figure S7. Selected details of the ESI-MS (neg. mode) spectra, showing signals corresponding to molecular formulae of glucosamine/N-acylurea functionalized species. Whilst the meso-Cl derivative **1-Glu2** (compound **6**) clearly forms according to HRMS, its isolation in pure form from the complex mixtures was not possible, and it occurred in the presence of **7** and **8** only.

Treatment of Compound 1 with EDCI and NHS in the absence of base

Compound **1** (78.9 mg, 93.6 μmol) was mixed with EDCI (44.8 mg, 234 μmol)) in DMF. The mixture was stirred for thirty minutes, before N-hydroxysuccinimide (22.6 mg, 196 μmol) was added. The resulting mixture was stirred overnight at room temperature. Following, this, the product was precipitated using a mixture of acetonitrile and diethyl ether and dried *in vacuo*. A dark green product was isolated (124.3 mg). **Figure S8** shows the mass spectrum of the isolated product.

Following the treatment of dye **1** with EDCI and NHS in the absence of base, mass spectrometry revealed signals corresponding to the NHS-functionalized dye **1-OSu**. While this mass spectrum contains numerous signals which were difficult to assign, the predominant species appear to be the unfunctionalized cyanine dye **1** and its succinimide-activated analogue **1-OSu**. The keto-polymethine derivative could only be detected in traces in this mixture (Figure S8).

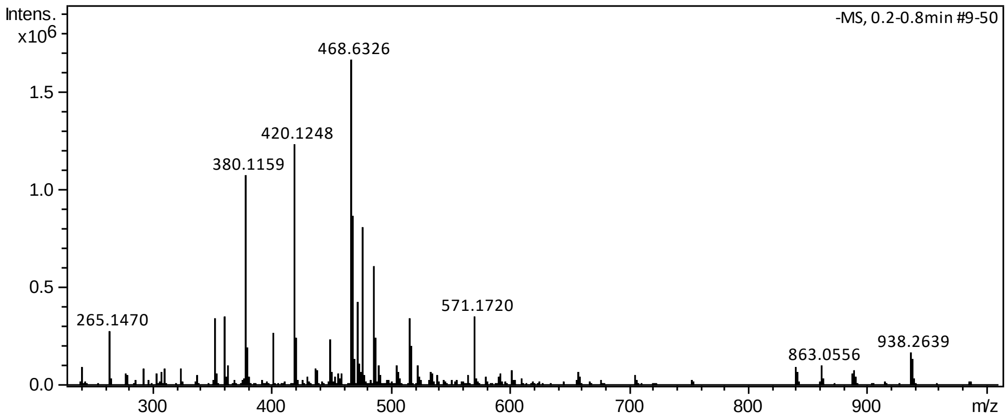

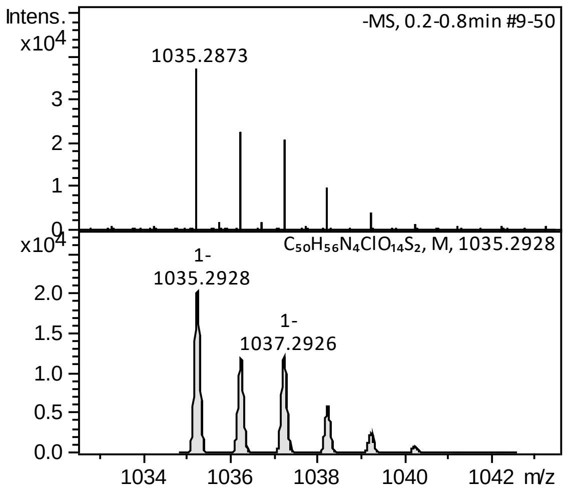

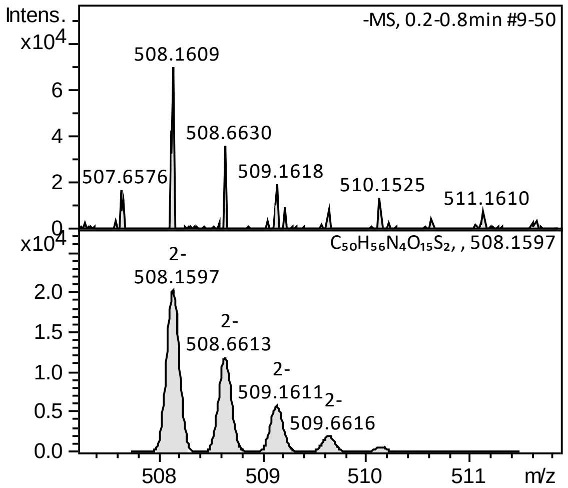

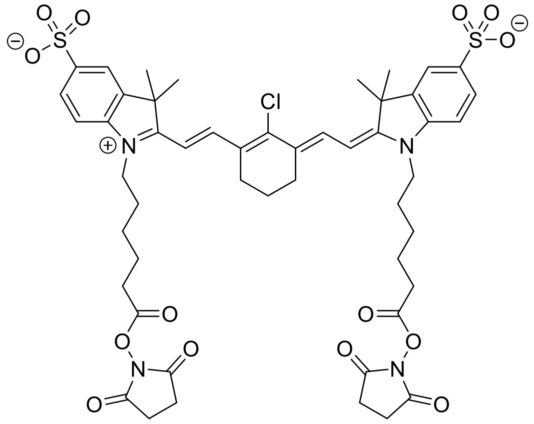

9-Cl

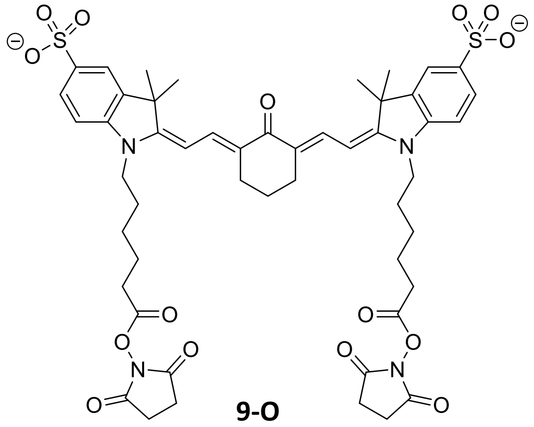

9-O

Figure S8. Selected details of the ESI-MS (neg. mode) spectra, showing signals corresponding to molecular formulae of succinimide esters intermediates **9-Cl** and **9-O** with proposed structures as indicated above.

EuK (LysCoGlu) and its coupling reactions of 1

EuK is a popular high-affinity ligand of PSMA, a membrane-bound protein which is upregulated in the majority of PCa cases. This can be synthesized in a simple three/four-step procedure, from readily available starting materials. Here, carbonyldiimidazole was chosen as a safer alternative to phosgene or triphosgenefor the synthesis of EuK. It is noteworthy here that, due to the lower reactivity of CDI, activating agents are necessary in the synthesis of EuK, e.g., 4-dimethylaminopyridine (DMAP) as a ‘nucleophilic catalyst’ in the synthesis of the intermediate denoted **10** and the route adopted for the synthetic steps and conditions involved to obtain the protected compound **11**. Overall, this reaction was performed using adapted literature methods.[17-20]

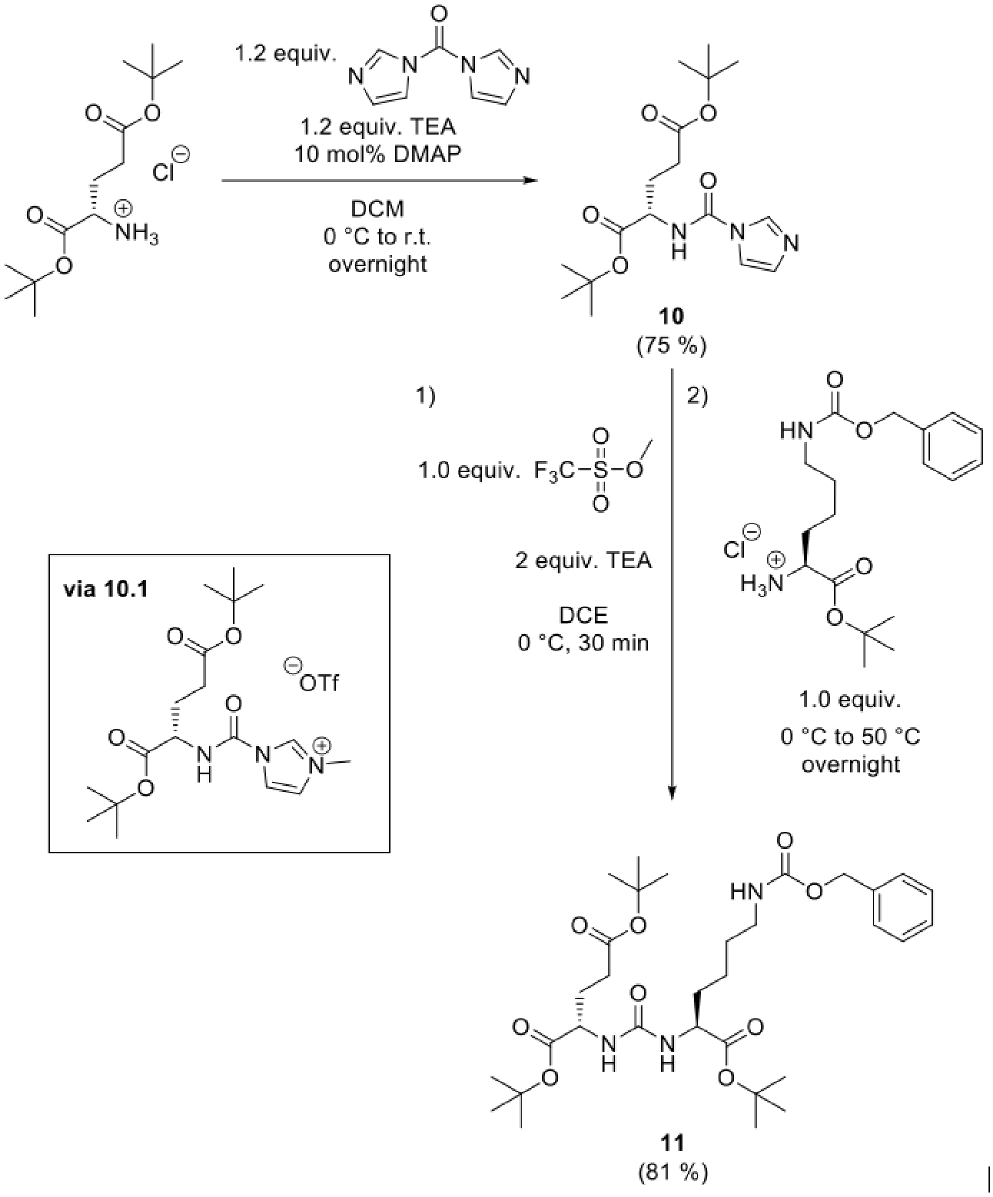

Scheme S8. Synthesis of **10** and **11**. Insert shows the proposed reactive intermediate **10.1** formed in the reaction of **10** and trifluoromethanesulfonate.

Here, the synthesis of **11** was performed by just heating the mixture to reflux, although in the course of this work, reactions employing trifluoromethanesulfonate gave slightly higher yields and proceeded quicker. The orthogonal protecting groups (Cbz/Z) at the ε-amine group, and Boc at the carboxylic acids can be removed selectively, such that these do not interfere in subsequent coupling reactions. For the case of derivative **11** the deprotection of the Cbz group was performed using ammonium formate in the presence of palladium on carbon to give product **12**.

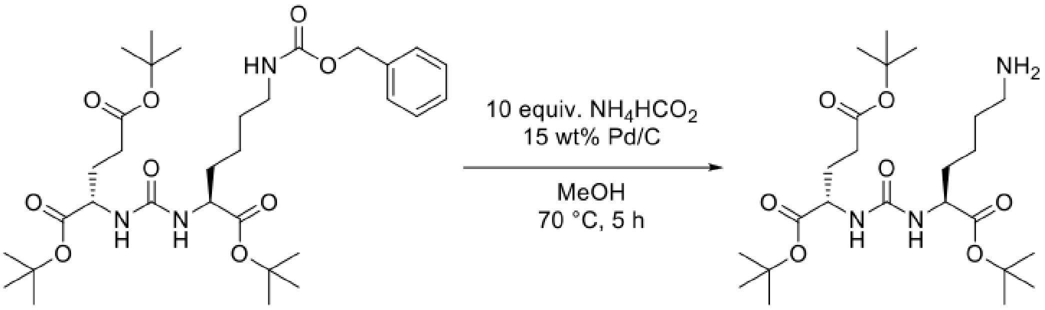

11 **12** (97 %)

Scheme S9 Reductive deprotection of 11 employing ammonium formate and palladium/C.

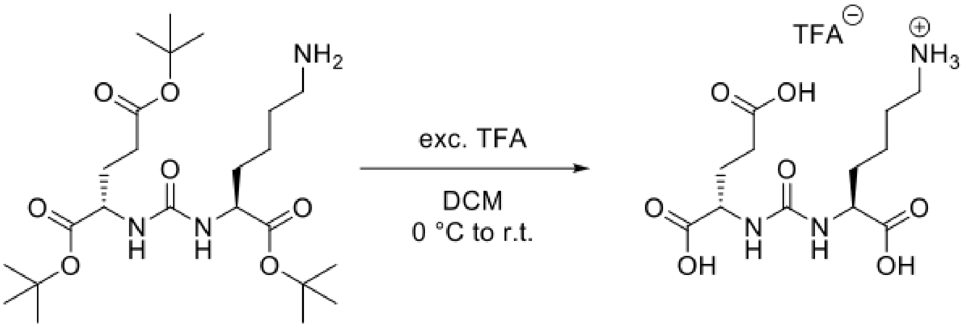

12 **13** (71 %)

Scheme S10 Deprotection of 12 by standard acidic cleavage of tert-butyl esters to give the resulting EuK (**13**) which was then used for further conjugation reactions.

Stepwise synthesis for EuK (LysCoGlu)

Step 1

In a 100 mL round-bottom flask, *L*-glutamic acid di-*tert*-butyl ester hydrochloride (2.53 g, 8.6 mmol), was suspended in 25 mL of dichloromethane. To this, triethylamine (1.42 mL, 10.3 mmol) and 4-dimethyl aminopyridine (DMAP, 107 mg, 0.83 mmol) were added. The resulting solution was cooled to 0 °C with an ice-bath. Subsequently, carbonyldiimidazole (1.69 g, 10.4 mmol) was added quickly. The mixture was allowed to warm to room temperature overnight. It was diluted with ca. 25 mL of dichloromethane, washed with a saturated solution of sodium carbonate (2 x 30 mL) and deionized water (1 x 40 mL). The organic layer was dried over magnesium sulfate, filtered and concentrated under reduced pressure. The obtained oily residue was filtered over a silica plug, and the product eluted with ethyl acetate. Concentration of the obtained solution gave **10** as a colorless solid (2.28 g, 75 %).3

^{1}H NMR (400.13 MHz, $CDCl_3$, 298 K): δ = δ 8.65 (d, 1H), 8.27 (s, 1H), 7.71-7.72 (m, 1H), 7.05-7.04 (m, 1H, $^{3}J_{HH}$ = 3.0 Hz), 4.25 (m, 1H), 2.39-2.36 (t, $^{3}J_{HH}$ = 4.5 Hz, 2H), 2.09-1.89 (m, 2H), 1.42 (m, 9H), 1.38 (m, 9H).

^{13}C {^{1}H} NMR (100.16 MHz, DMSO-d_6, 298 K): δ = 171.6, 170.8. 149.0, 136.1, 129.6, 116.7, 81.3, 79.9, 53.7, 31.2, 27.7, 27.6, 25.7

ESI-MS – negative mode [m/z] calculated for $C_{17}H_{27}N_3O_5Na$ $[M+Na]^+$ = 376.1849, found, 376.1847, deviation = 0.53 ppm

Step 2

In a 100 mL round-bottom flask, **10** (2.21 g, 6.27 mmol), was suspended in 25 mL of dichloroethane (DCE). To this, triethylamine (1.73 mL, 12.5 mmol) was added. The resulting solution was cooled to 0 °C with an ice-bath. Subsequently, methyl trifluoromethanesulfonate (0.68 mL, 6.27 mmol) was added dropwise. The mixture was allowed to stir for thirty minutes, before *L*-lysine *tert*-butyl ester-e-benzyl carbamate (2.33 g, 6.27 mmol) was added. The ice-bath was removed and the mixture heated to 50 °C for six hours or until TLC indicated complete conversion. Subsequently, the mixture was diluted with ca. 25 mL of dichloromethane and washed with a saturated solution of sodium carbonate (2 x 30 mL) and deionized water (1 x 40 mL). The organic layer was dried over magnesium sulfate, filtered and concentrated under reduced pressure. The obtained oily residue was loaded onto a silica column, and the product eluted with ethyl acetate. Concentration of the obtained fractions gave **11** as a colorless oil (3.38 g, 81 %).

^{1}H NMR (400.13 MHz, $CDCl_3$, 298 K): δ = 7.34 (m, 5H), 5.33-5.28 (m, 3H), 5.08 (d, $^{3}J_{HH}$ = 7.4 Hz, 2H), 4.38-4.29 (m, 2H), 3.15 (m, 2H), 2.32-2.01 (m, 2H), 1.90-1.50 (m, 8H), 1.43-1.40 (m, 27H)

ESI-MS – negative mode [m/z] calculated for $C_{33}H_{52}N_3O_{11}$ $[M+FA]^-$ = 666.3602; found, 666.3609;

3 In some instances, an oily residue remains, which is difficult to dry using a rotary evaporator. In these cases, blowing a stream of nitrogen over the oily/waxy substance for several minutes usually succeeds in causing solidification and removal of remaining solvent traces.

Step 3

Activated palladium on carbon (10 % Pd, 0.25 g) was added portion wise to a solution of **11** (1.66 g, 3.4 mmol) in ethanol (50mL), followed by 2.14 g (34 mmol) ammonium formate. The reaction mixture was stirred at room temperature overnight, before it was filtered through celite to remove the Pd/C. The filtrate was concentrated under vacuum to give **12** as a viscous oil (1.257 g, 97 %).

^{1}H NMR (400.13 MHz, DMSO-*d*$_6$, 298 K): δ = 7.15-6.0 (b, 4H), 4.29 (m, 2H), 3.02 (m, 2H), 2.33 (m, 2H), 2.06-1.47 (m, 8H), 1.45-1.40 (m, 27H)

Step 4

In a 25 mL round-bottom flask, TFA (1.6 mL) in DCM (6.3 mL) was added to **12** (1.173 g) and left to stir over night in order to deprotect the t-Bu protecting groups. After concentration under a stream of nitrogen, the product was purified by flash column chromatography over reverse-phase silica using a water/EtOH gradient (0 – 50 % MeOH, 0.1 % TFA in mobile phases). Lyophilization afforded compound **13** as a hygroscopic solid (427 mg, 71 %).

^{1}H NMR (400.13 MHz, DMSO-*d*$_6$, 298 K): δ = 8.67-8.65 (d, J_{HH} = 7.9 Hz, 2H), 7.75 (s, 2H), 4.60-4.58 (m, 1H), 5.26-3.28 (m, 1H), 2.78-2.76 (t, $^{3}J_{HH}$ = 4.0 Hz, 2H), 2.63-2.58 (m, 2H), 1.57-1.53 (m, 2H)

ESI-MS – positive mode [m/z] calculated for $C_{12}H_{22}N_3O_7$ $[M+H]^+$ = 320.1458; found = 320.1451; deviation = 2.19 ppm.

General protocol for the coupling reactions of 1 with EuK (Compound 13)

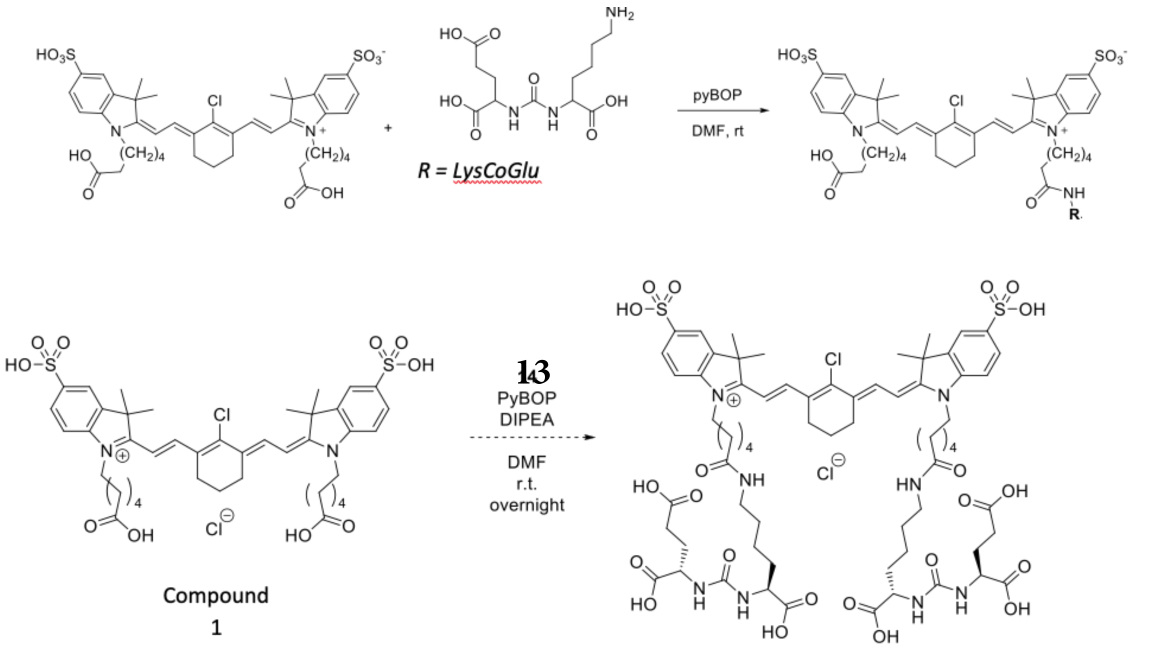

Scheme S11 Coupling reactions aimed at obtaining a bi-substituted EuK-functionalized derivative. Attempts at performing conjugation reactions with meso-Cl cyanine dyes were initially performed using the peptide-like molecule EuK (or LysCoGlu, **13**) using the coupling agent PyBOP. Reactions were performed in the approximative ratio 1:1:1:1.5 or 1:1:1:2 for Compound **1**:DIPEA:pyBOP:LysCoGlu).

Compound **1** (4 mg, 0.842 mg, 4.75 μmol, considered in its zwitterionic form shown above) was suspended in 1.5 mL DMF. To this, pyBOP (2.5 mg, 4.75 μmol) and DIPEA (0.122 mL, 0.61 mg, 4.75 μmol) were added as 0.5 mL aliquots of each of the coupling reagents from their corresponding stock solutions in DMF. Then the reaction mixture was stirred at the room temperature for 1.5 h. The reaction was followed by TLC with DCM:MeOH. Then the compound EuK (LysCOGlu, **13**) (3.2 mg, 10 μmol, for the 1:1:1:1 reaction, or in excess, aiming for the complete conversion) was added to the reaction mixture from a stock solution in DMF (1 mL). Subsequently the reaction mixture was heated at 50°C for 48 h. Then the solvent was removed under reduced pressure and purification was carried out by semiPrepHPLC on a C18 column, using H_2O:CH_3CN, 0.1% TFA. The isolated fractions, after solvent removal under educed pressure were dark-green solids. These were analyzed by ESI+ mass spectrometry. Alternative protocols involved reaction being performed under stirring at the room temperature for 48 h, and several reactants' ratios as well as reaction scales (from microgram to mg scale) were explored. Several repeats of this reaction protocol including optimizations of reaction conditions indicated that the *meso*-Cl dye, under the influence of HOBt or NHS based coupling reagents (e.g. HBTU, PyBOP, HSPyU), gives a mixture of products, dominated by the formation of the corresponding keto-polymethine derivatives. Coupling reaction attempts using just carbodiimides (DCC, EDCI) and no other additives seem to lead to some conversion but proceeded in a rather slow way, even if heated. In terms of its generality, the avoidance of active ester strategies does not readily lead to the desired EuK conjugates and standard deprotection methods led to dye decomposition, (e.g. hydrogenation of Cbz/Boc groups, TFA deprotection). This might explain why the isolation on a laboratory scale of the EuK derivative of compound **1** using EDC alone and without using protecting groups was not successful: we assigned this to the combination of poor kinetics and side reactions. UV-vis investigations of the crude mixture revealed a drastic change in the absorption spectrum. Purification by semi-preparative HPLC of the reaction mixtures consistently lead to isolation of the desired dye in yields of less than ca. 10%. Traces of the desired product (the meso-Cl substituted and simultaneously EuK conjugated derivative) have been isolated albeit the corresponding ketopolymethine derivative was also present as an impurity on the analytical scale HPLC. Presence of TFA from semipreparative separation eventually led to protonation and decomposition of this keto-polymethine derivative. By semiPrepHPLC, the difference between the retention times of the *meso*-Cl derivative and corresponding keto-polymethine conjugate of EuK was found to be minimal. The UV-vis spectroscopy of isolated fractions seems to suggest that the absorption maximum of keto-polymethine-EuK conjugate is broad, and a characteristic band occurs at ca. 560 nm in water, compared to the original starting material compound 1. A small, as-isolated sample of the desired *meso*-Cl-EuK conjugate showed a similar behavior to that of the starting material compound 1, however significant broadening was observed in its UV-vis spectrum. Mass spectrometry of the reaction mixture contained evidence of oxo species formation as well as a tiny peak assignable to the expected *meso*-Cl-EuK conjugate, denoted compound **14**. Therefore, we concluded that attempts at isolating the desired compound **14** by semipreparative HPLC led to only a very low quantity of isolated product and the formation of the corresponding oxo-derivative **15** as an impurity was proposed.

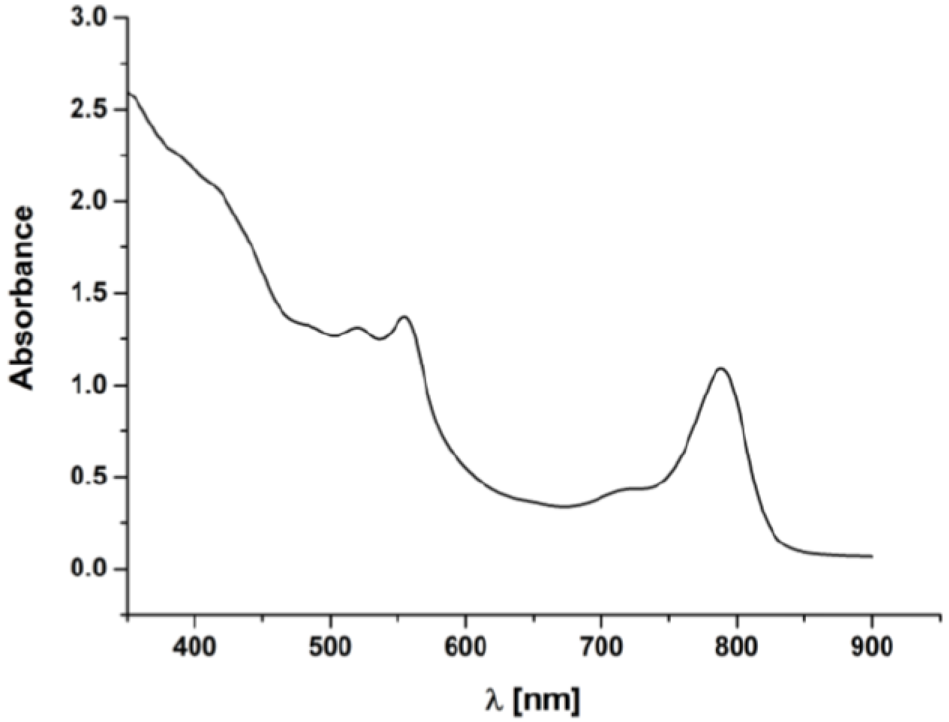

Figure S9. The UV/Vis spectrum of a sample corresponding the reaction mixture of Compound **1** and LysCoGlu (EuK, **13**) in the presence of PyBOP in DMF.

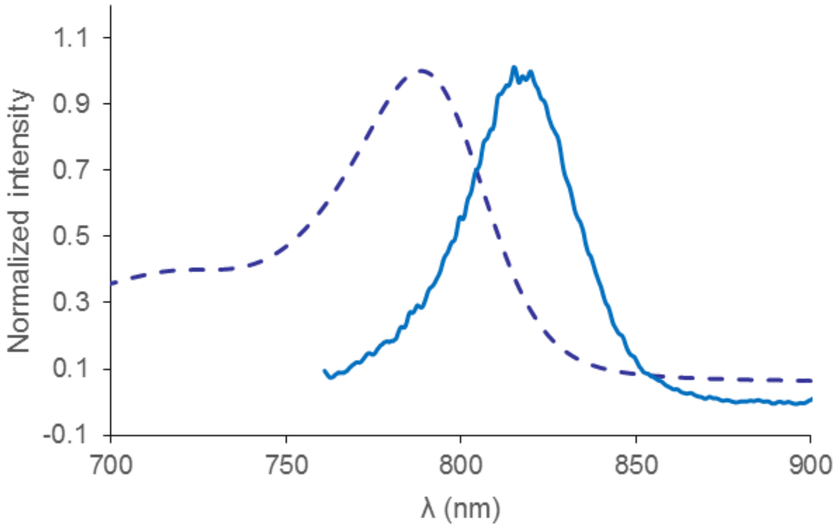

Figure S10. Normalized absorption and emission spectra assigned to the meso-Cl-EuK conjugate dye afetr extensive separation and purification from the mixture described above (Figure S9). This species is postulated as **14** in (10 μM conc.) in water ($\lambda_{max\text{-}abs}$: 791 nm; $\lambda_{max\text{-}em}$: 820 nm) and spectrscopy was carried out after isolation from semiPrep HPLC.

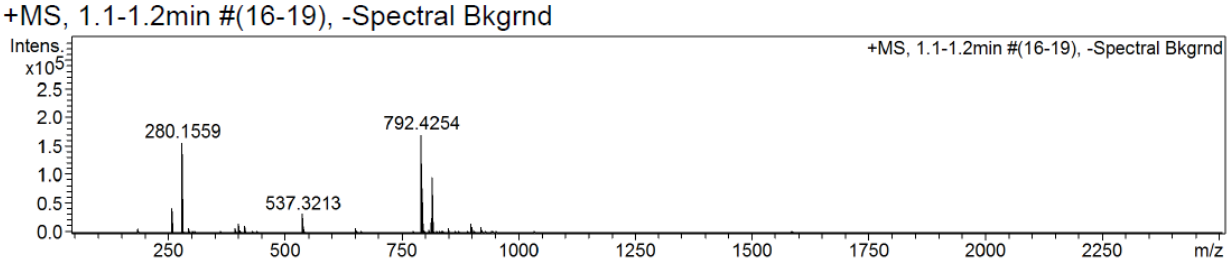

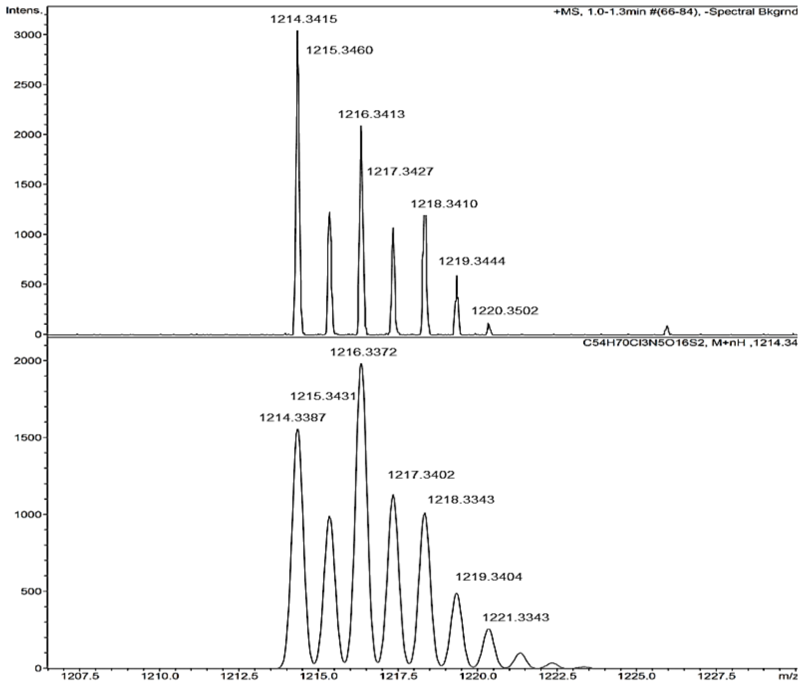

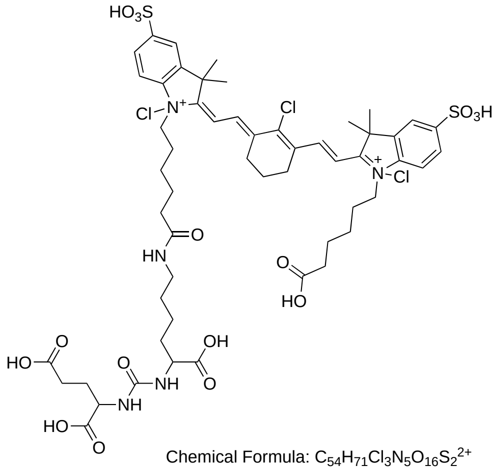

Chemical Formula: $C_{54}H_{71}Cl_3N_5O_{16}S_2^{2+}$
Exact Mass: 1214.3392
Molecular Weight: 1216.6499
m/z: 607.1696 (100.0%), 608.1681 (95.9%), 607.6713 (58.4%), 608.6698 (56.0%), 609.1667 (30.6%), 609.6683 (17.9%), 608.1730 (16.7%), 609.1715 (16.1%), 608.1675 (9.0%), 609.1660 (8.7%), 608.6692 (5.3%), 610.1700 (5.1%), 609.6677 (5.1%), 608.1717 (3.3%), 610.1652 (3.3%), 609.1703 (3.2%), 608.6746 (3.1%), 609.6732 (3.0%), 610.1646 (2.8%), 608.6734 (1.9%), 610.6669 (1.9%), 607.6681 (1.8%), 609.6719 (1.8%), 608.6667 (1.8%), 610.6662 (1.6%), 607.6693 (1.6%), 608.6678 (1.5%), 609.1709 (1.5%), 610.1694 (1.5%), 608.1698 (1.1%), 610.1688 (1.0%), 609.1683 (1.0%), 610.6717 (1.0%)

Figure S11. Mass spectrometry of the isolated product mixture from the reaction in Scheme S11 (reactants ratio 1:1:1:1.5 for Compound **1**:DIPEA:pyBOP:LysCoGlu), with the postulated structure above (**14**), possibly indicated by HRMS as a 2HCl adduct.

Kinetic Stability of Keto-Polymethine 2

Over the course of these investigations, the stability of keto-polymethines under the influence of light and various (endogenous) chemicals was assessed. Initially, a sample of dye **2** (10 μMolar concentration) was exposed to a light source, and the mass spectrum was measured, revealing decomposition products and intermediates, characteristic for the photobleaching of cyanine-based compounds.[4]

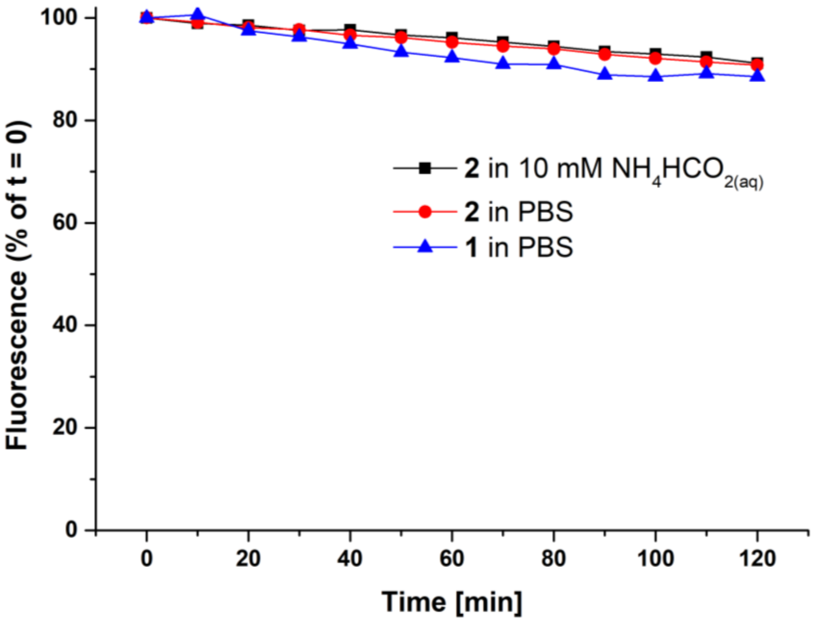

Figure S12. Photostability investigations for **1** in PBS, and **2** in PBS or 10 mM $NH_4HCO_{2(aq)}$

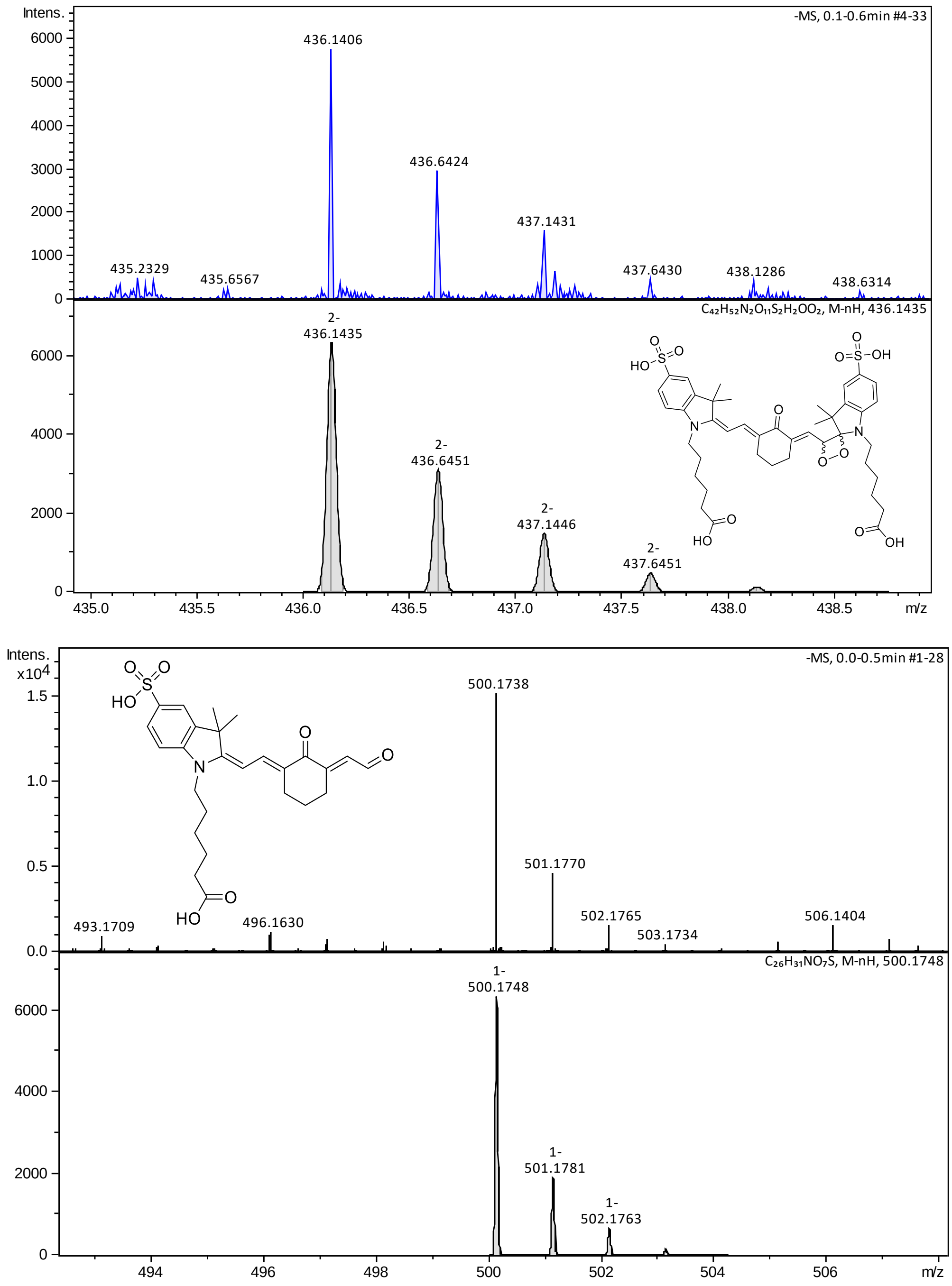

Figure S13. Top: Detail of the HRMS of a solution of **2** which was exposed to light, potentially corresponding to a singlet-oxygen adduct. Proposed structure of postulated reaction product with singlet oxygen. Bottom: Detail of the HRMS of a solution of **2** which was exposed to light, indicating the presence photobleaching product. Inlay: Proposed corresponding structure (cleavage product of the structure depicted in Top diagram).

Next, the influence of an acidic pH was investigated. A sample of dye **2** was dissolved in water or methanol. Two samples of each solution were used for the experiments. All solutions were prepared in the dark. One of the respective samples was kept in the dark, while the other one was exposed to light by repeatedly measuring UV/VIS spectra. Both samples were acidified by addition of 0.1 vol% of TFA. As expected, an immediate shift to ca. 720 nm can be observed by UV/VIS spectroscopy, followed by a steady decline of absorption at this wavelength, and emergence of a new signal at ca. 480 nm. After 20 minutes, the sample which was kept in the dark was investigated, indicating a similar loss in absorption at 720 nm. This result shows that the decomposition of protonated merocyanine may not primarily be due to photobleaching, but rather an inherent property. Solutions in organic solvents, such as methanol, show little to no loss of absorbance at 720 nm, suggesting an involvement of water in the decomposition pathway. Attempts to analyze such solutions by mass spectrometry failed to provide further insight.

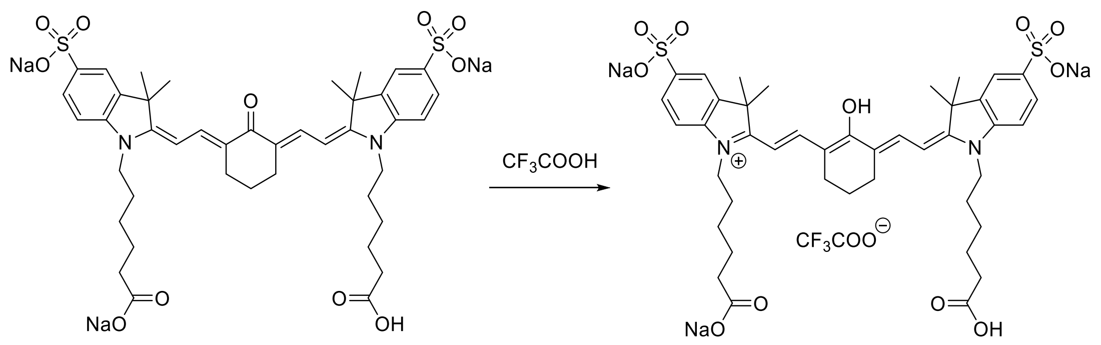

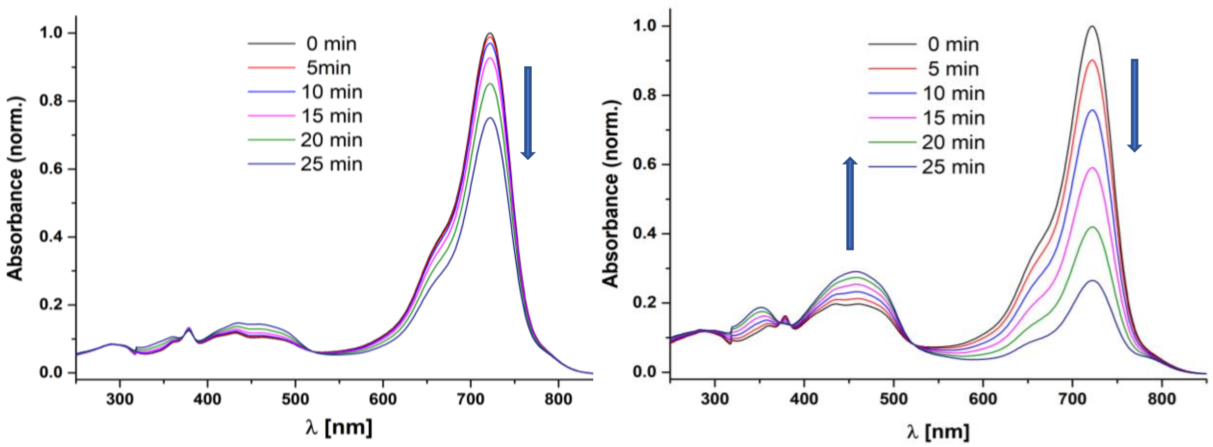

Figure S14. Normalized absorption spectra of acidified solutions of **2** in water (left, acidified with 0.1 vol% TFA; right, acidified with 0.2 vol% TFA) measured in 5-minute intervals. Despite almost complete protonation at 0.1 vol% TFA, further TFA clearly accelerates decomposition, indicating that photobleaching is not the primary decomposition pathway. In methanol, only a slow decline in

absorption is observed, indicating that the presence of water mediates the decomposition, indicated by an irreversible color change (increase at maximum absorption between 400 and 500 nm).

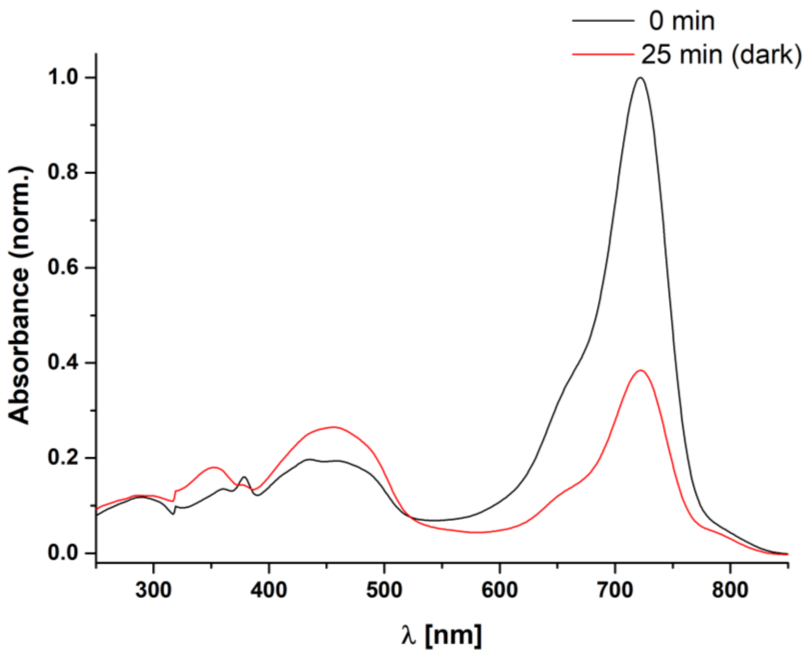

Figure S15. Normalized absorption spectra of a solution of **2** in water acidified 0.2 vol% TFA and kept in the dark in between measurements, further confirming that the decomposition is not primarily related to photobleaching.

To further assess the kinetic stability under milder acidic conditions a 0.1 M acetic acid/sodium acetate buffer with an approximate pH of 5 was prepared. Solutions of **2** in this mixture show slower decomposition, however, without formation of considerable quantities of the enol-form of **2**, **2-OH**. Noticeably, this is in the range of pH-values found inside of lysosomes,[5, 6] such that lysosomal uptake of **2**, and derivatives thereof may cause decomposition.

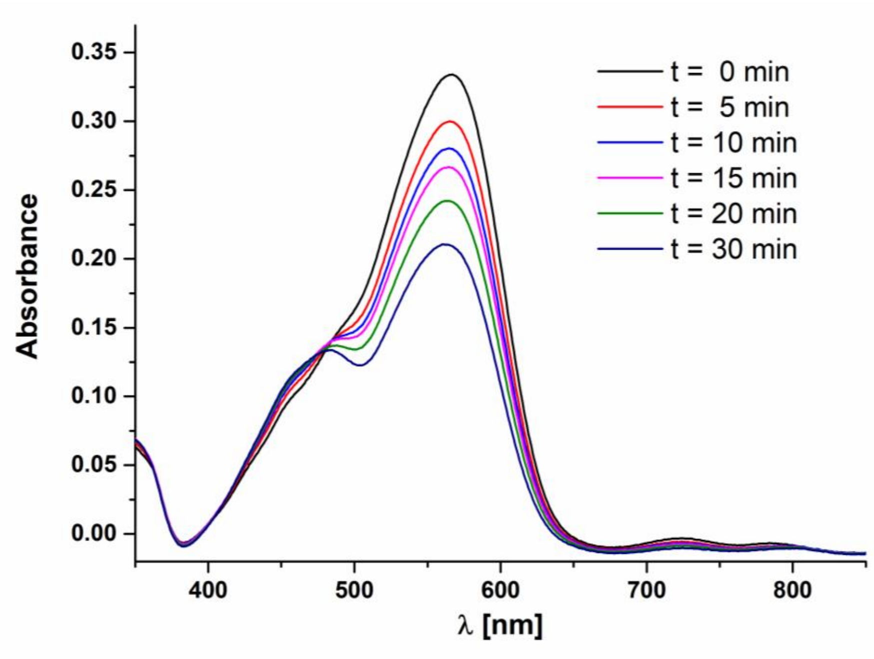

Figure S16. Absorption spectra of a solution of **2** in a 0.1 M aqueous acetic acid/sodium acetate buffer (pH ~ 5) measured in five-minute intervals.

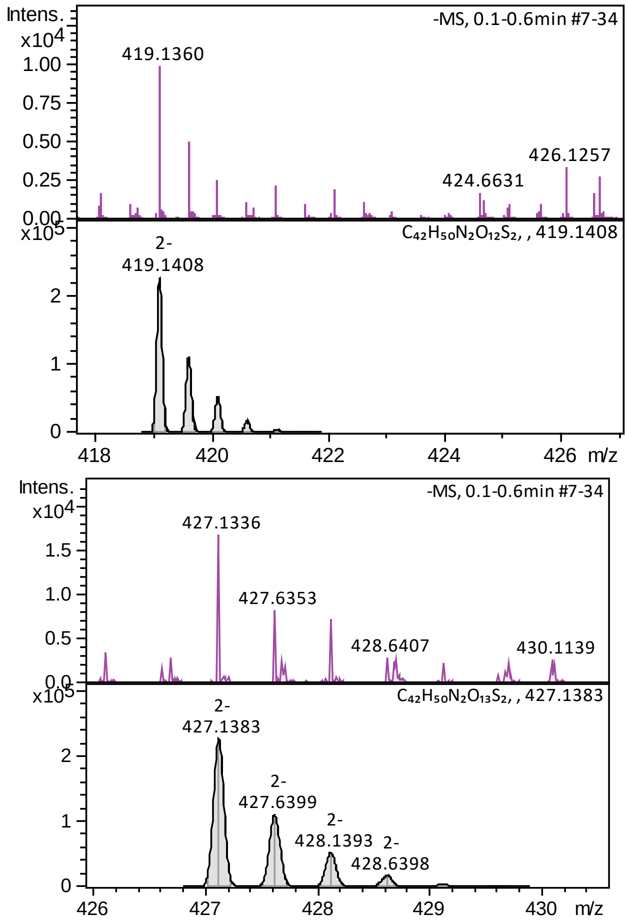

Figure S17. Mass spectrometry of Compound **2** acidified with 0.1 vol% FA (Neg. ESI-MS).

To assess and compare the kinetic stability in the presence of nucleophiles for dyes **1** and **2** was assessed in aqueous solutions containing 10 mM glutathione. Also, for assessing the influence of reducing agents the effects of 1 mM ascorbic acid, or of a 1:1 mixture of FBS and PBS, were monitored over defined time intervals (t = 0 to 4 hours in 15 min intervals, 24 and 48 hours). Dye **2** has high stability under these conditions, whereas dye **1** reacts with free thiols, causing a bathochromic shift, as has been observed for related *meso*-Cl dyes.[7] In contrast, keto-polymethine **2** appears not to react with reduced glutathione.

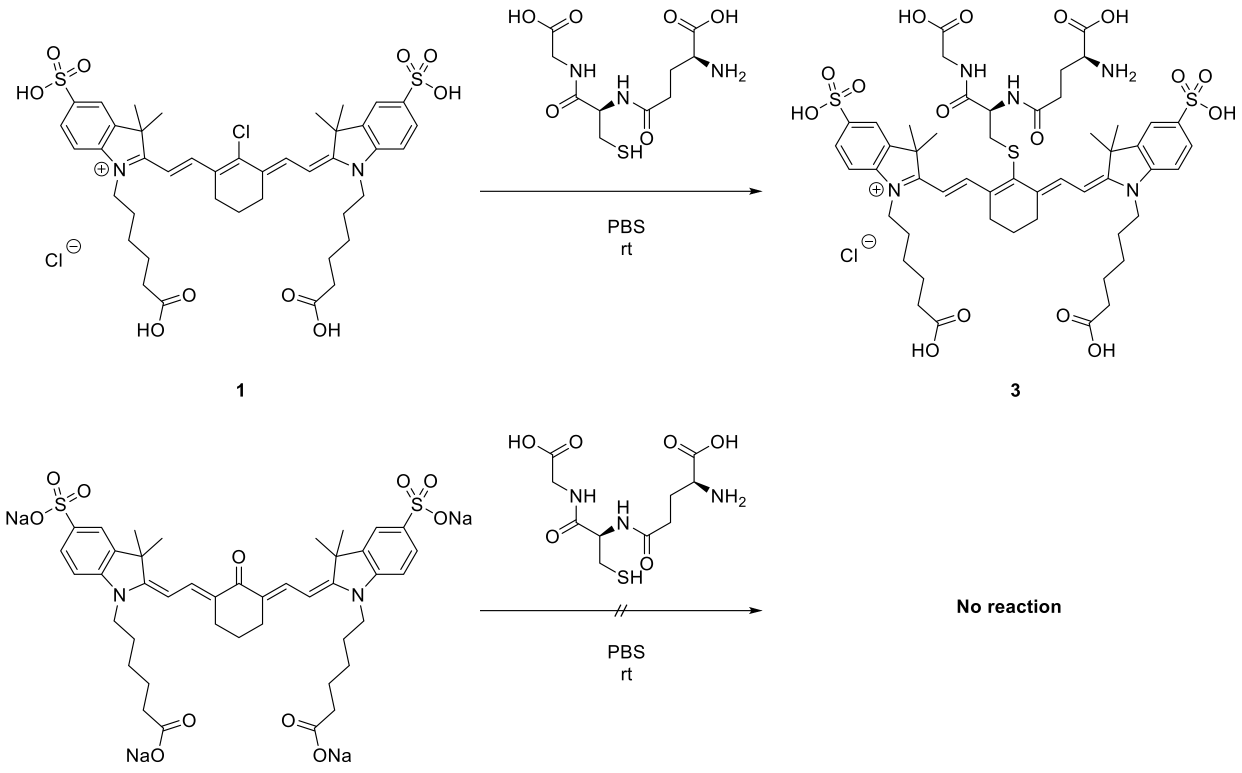

Scheme S12. Reaction between compound **1** and reduced glutathione in PBS yields the depicted meso-glutathione derivative **3**, while no reaction appears to occur between compound **2** and glutathione.

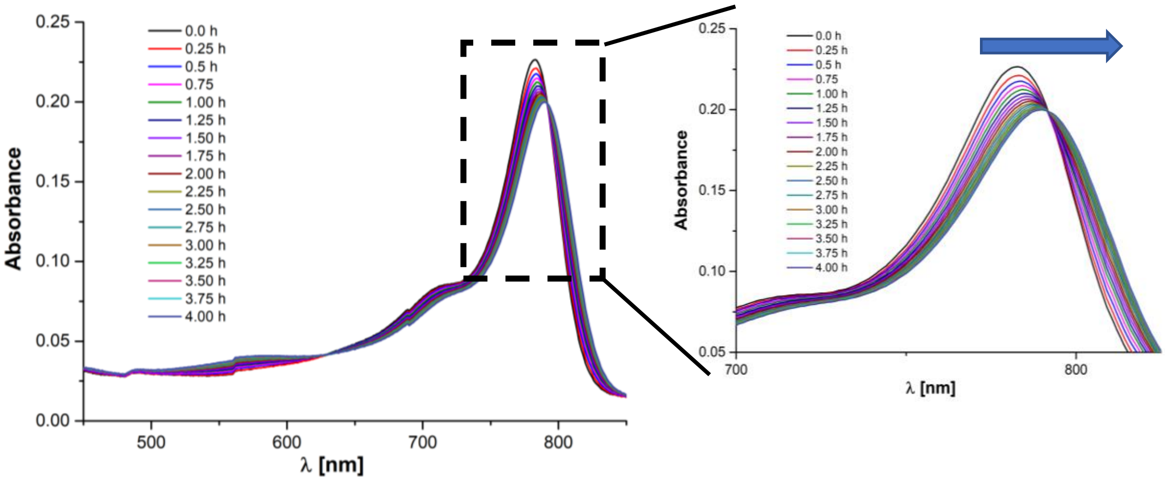

Figure S18. Change in the absorbance spectra of a solution of **1** and glutathione showing a bathochromic shift.

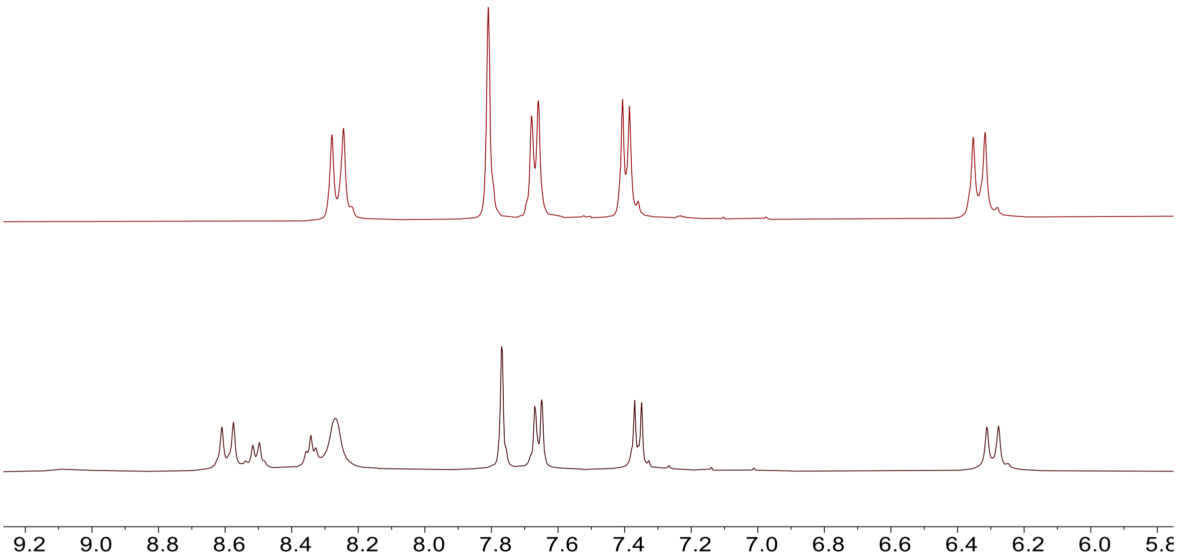

Figure S19. A comparison of the aromatic region of the ^{1}H-NMR spectra (400.13 MHz, DMSO-d$_6$, 298 K) of **1** (top) and **3** (bottom).

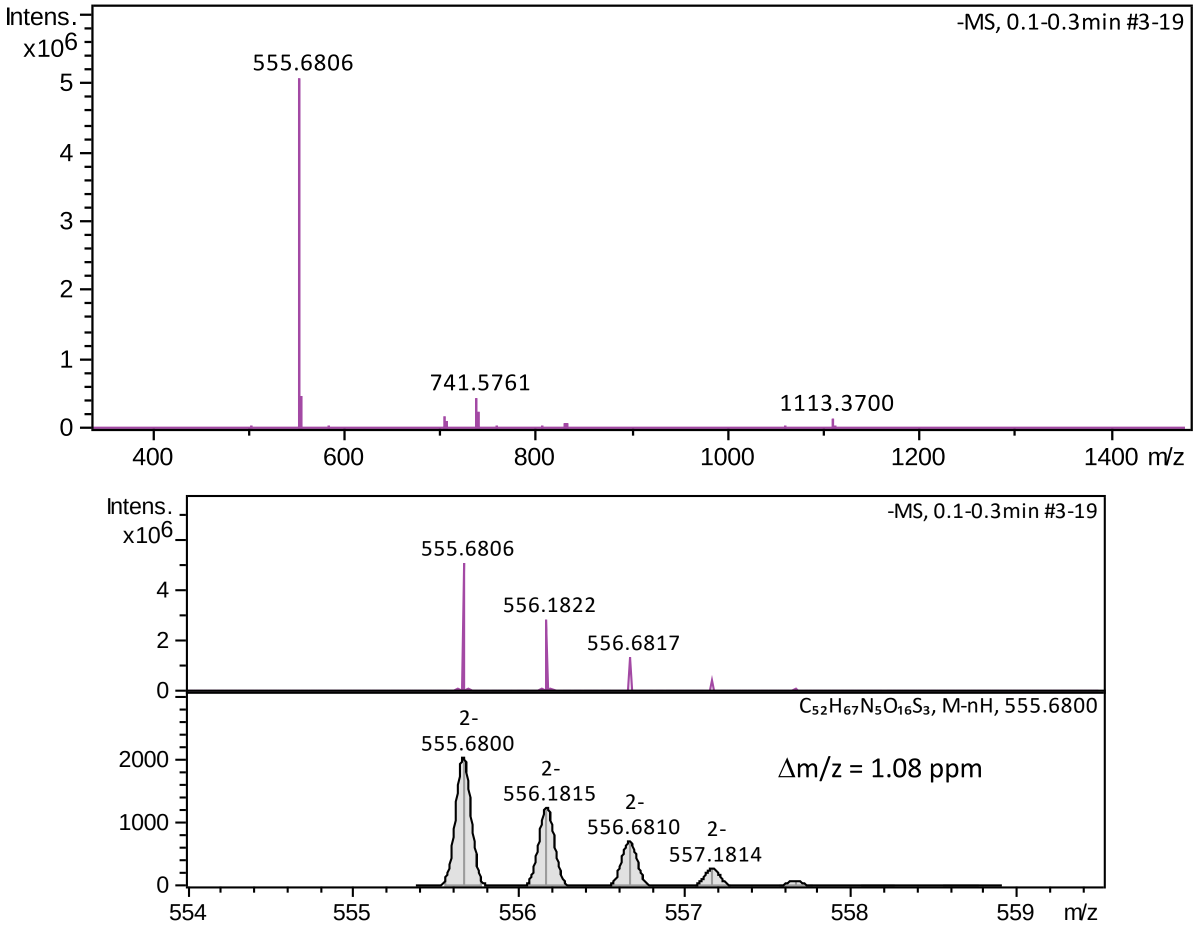

Figure S20. ESI-MS (neg. mode) of isolated and purified compound **3** (top), detail showing the experimental and predicted isotope pattern for $[M\text{-}2H]^{2-}$ (bottom).

Spectroscopic characterization Data for Compounds 1-5

Compound **1**

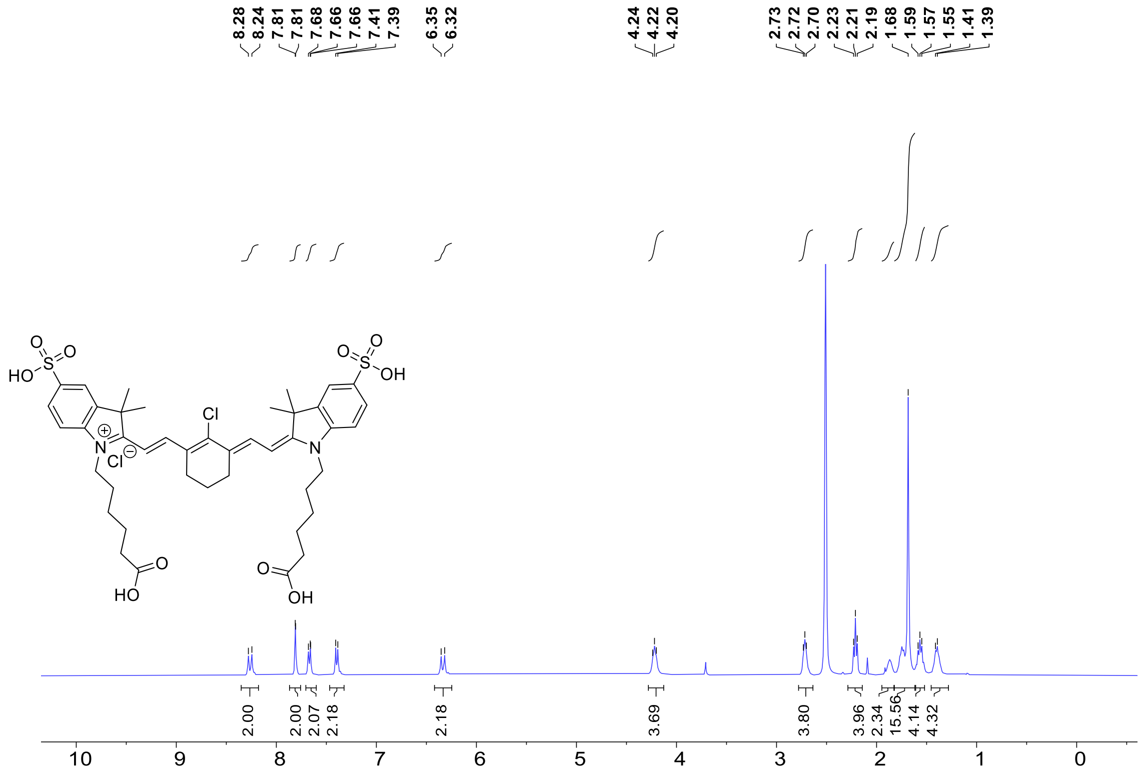

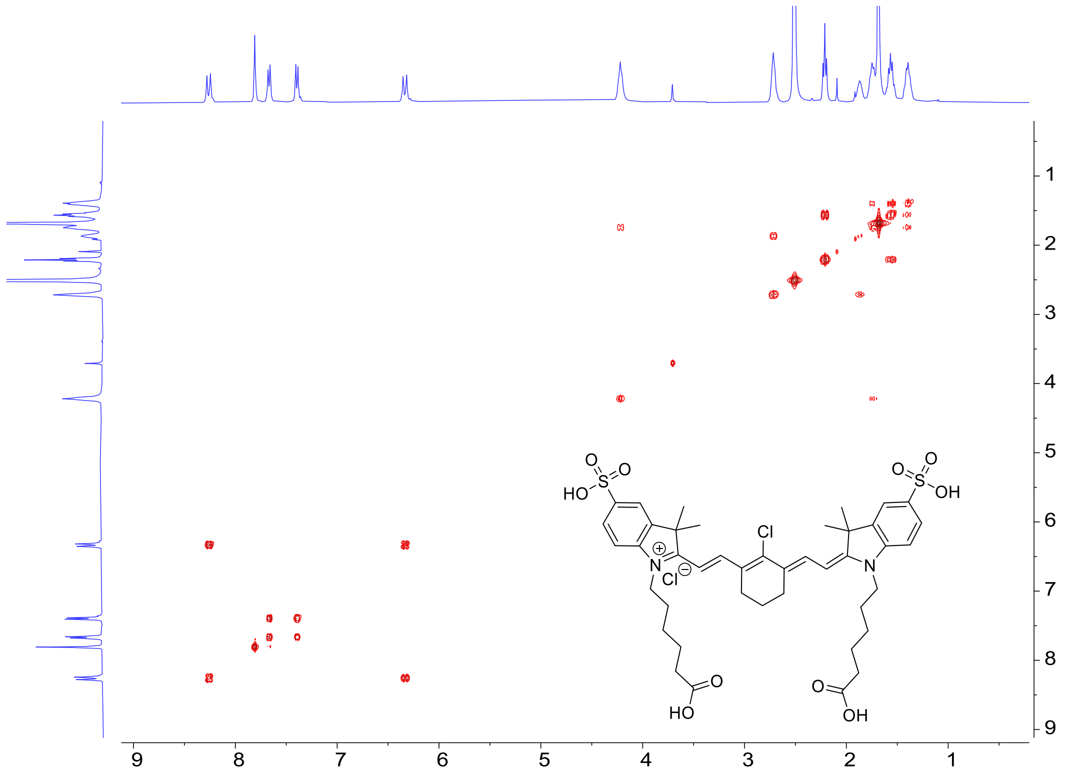

Figure S21. TOP: ^{1}H NMR spectrum (400.13 MHz, DMSO-d_6, 298 K) of compound **1;** Bottom: ^{1}H-^{1}H COSY NMR spectrum (400.13 MHz, DMSO-d_6, 298 K) of compound **1**

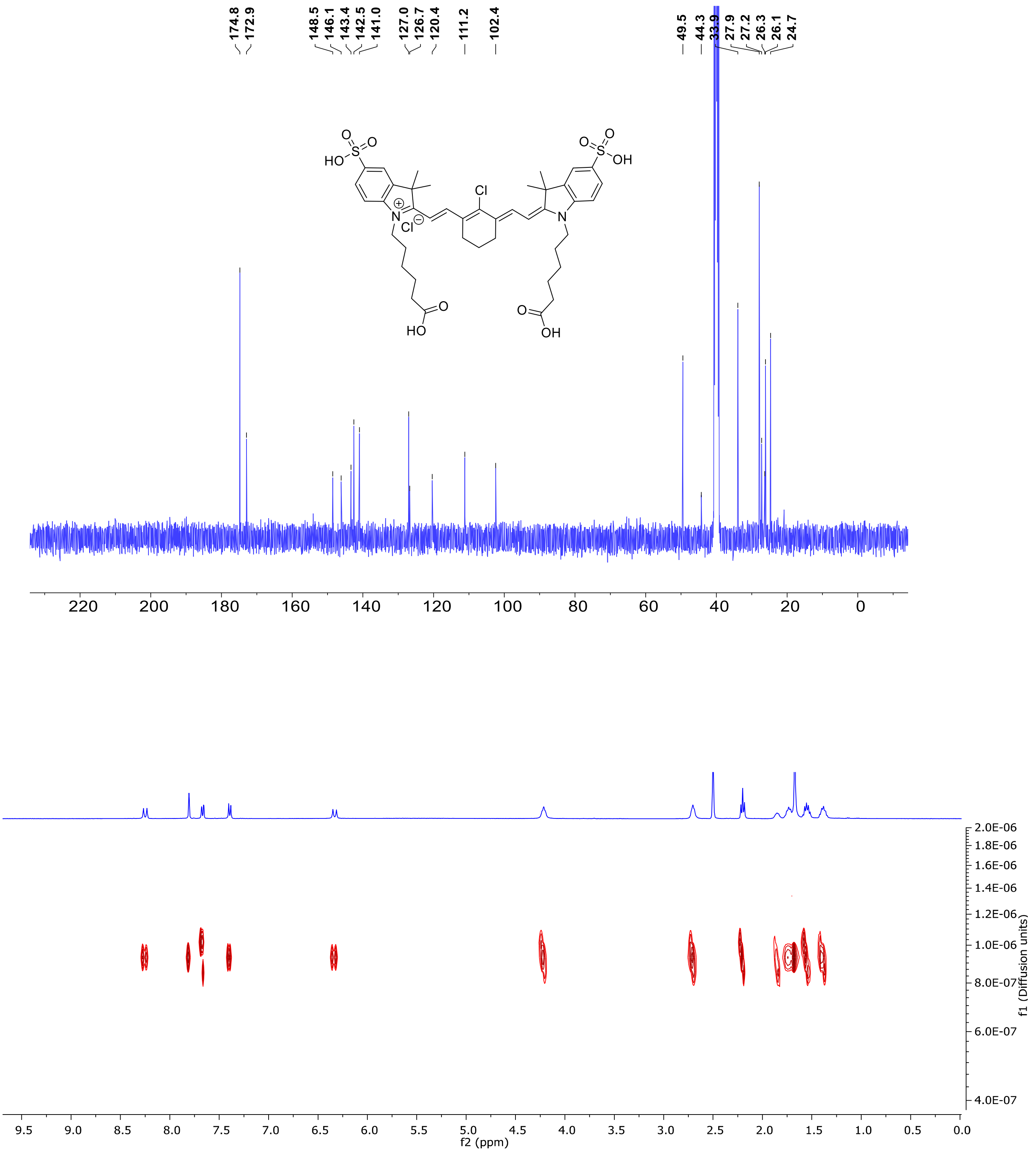

Figure S22. Top: $^{13}C\{^{1}H\}$ NMR spectrum (100.16 MHz, CD_3OD, 298 K) of compound **1;** Bottom: DOSY-NMR spectrum (400.13 MHz, DMSO-d_6, 298 K) of compound **1**.

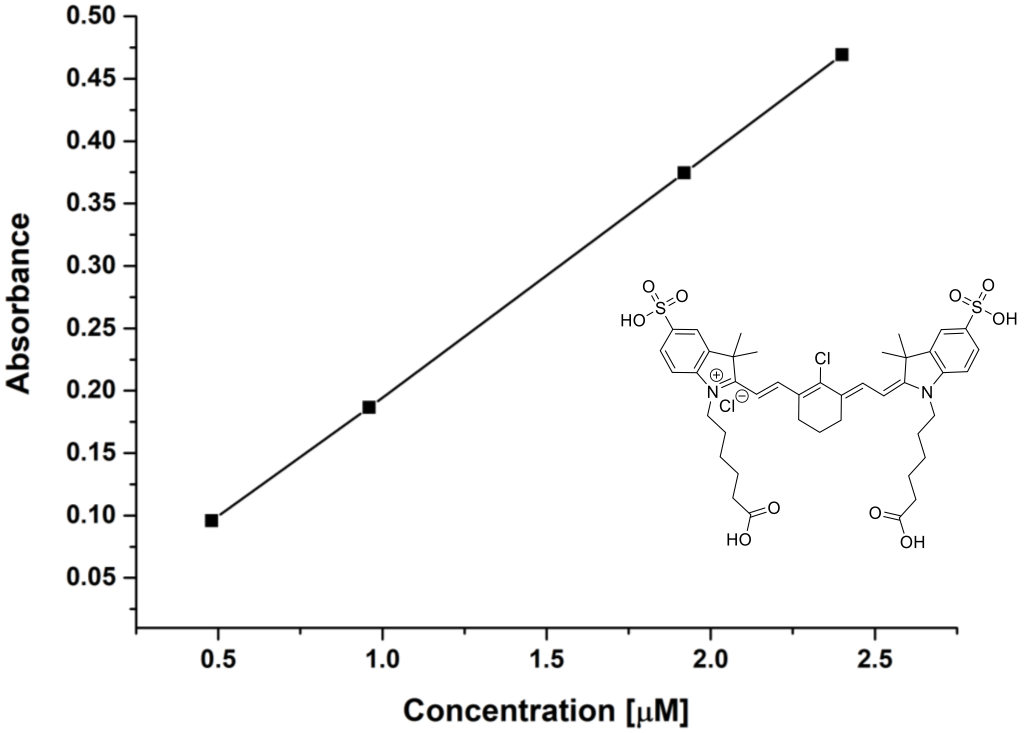

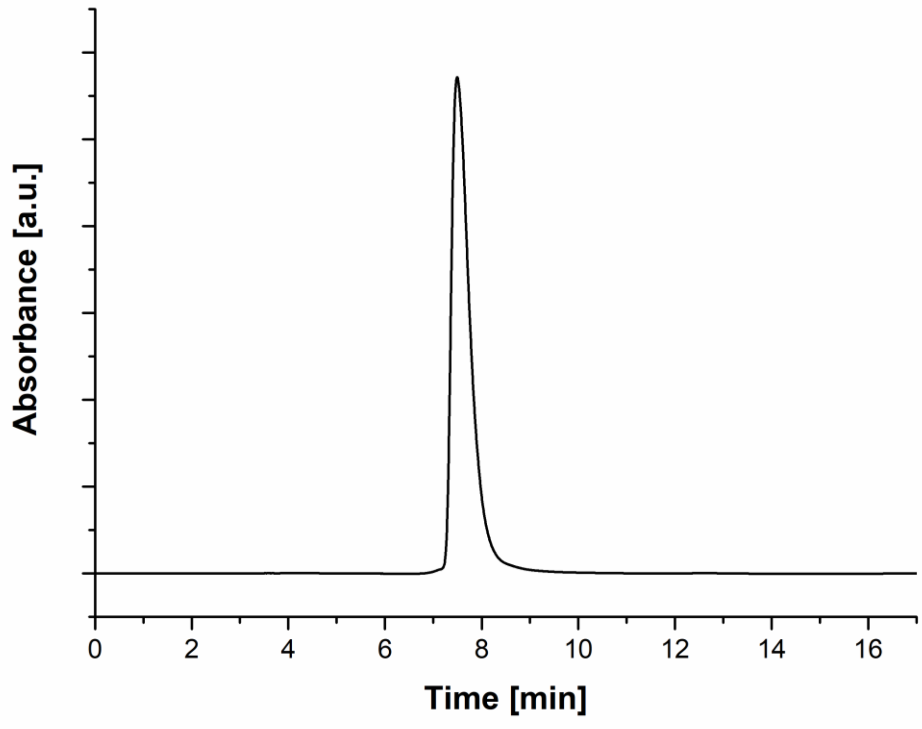

Figure S23. Top: Concentration-dependent absorption of compound **1** following the *Beer-Lambert* law; Bottom: Analytical HPLC trace (780 nm) of compound **1**; Method A.

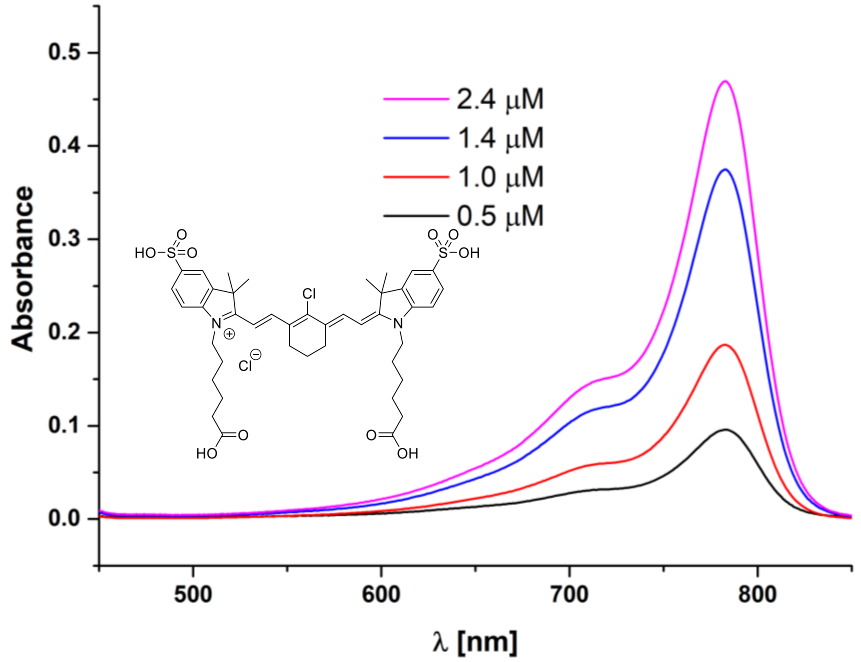

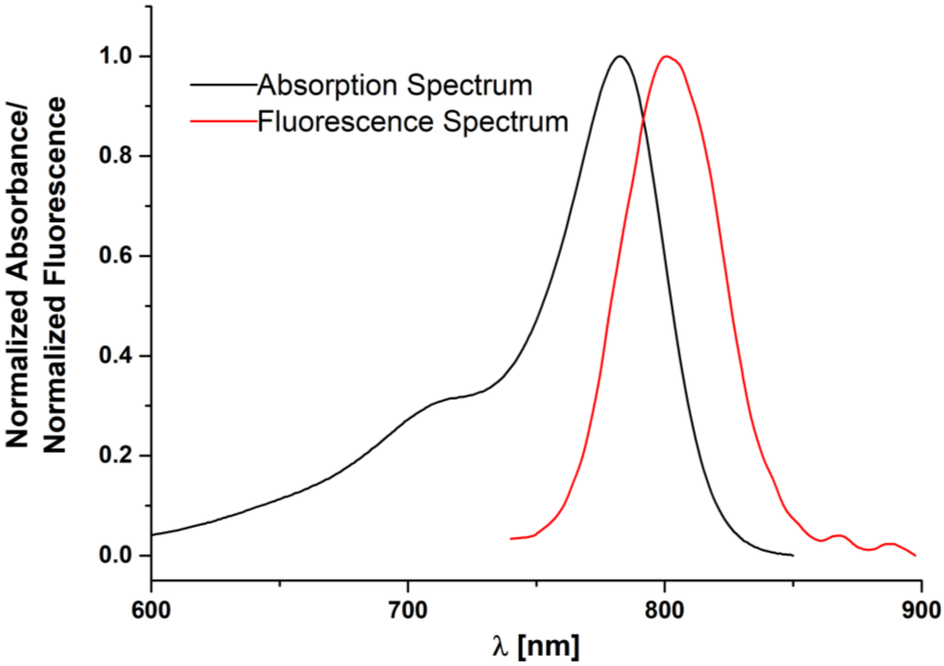

Figure S24. Top: Absorption spectra of compound **1** in PBS at various concentrations; Bottom: plot of the normalized Absorption/Emission Spectra of **1** in PBS (bottom) at 2 μMolar conc.

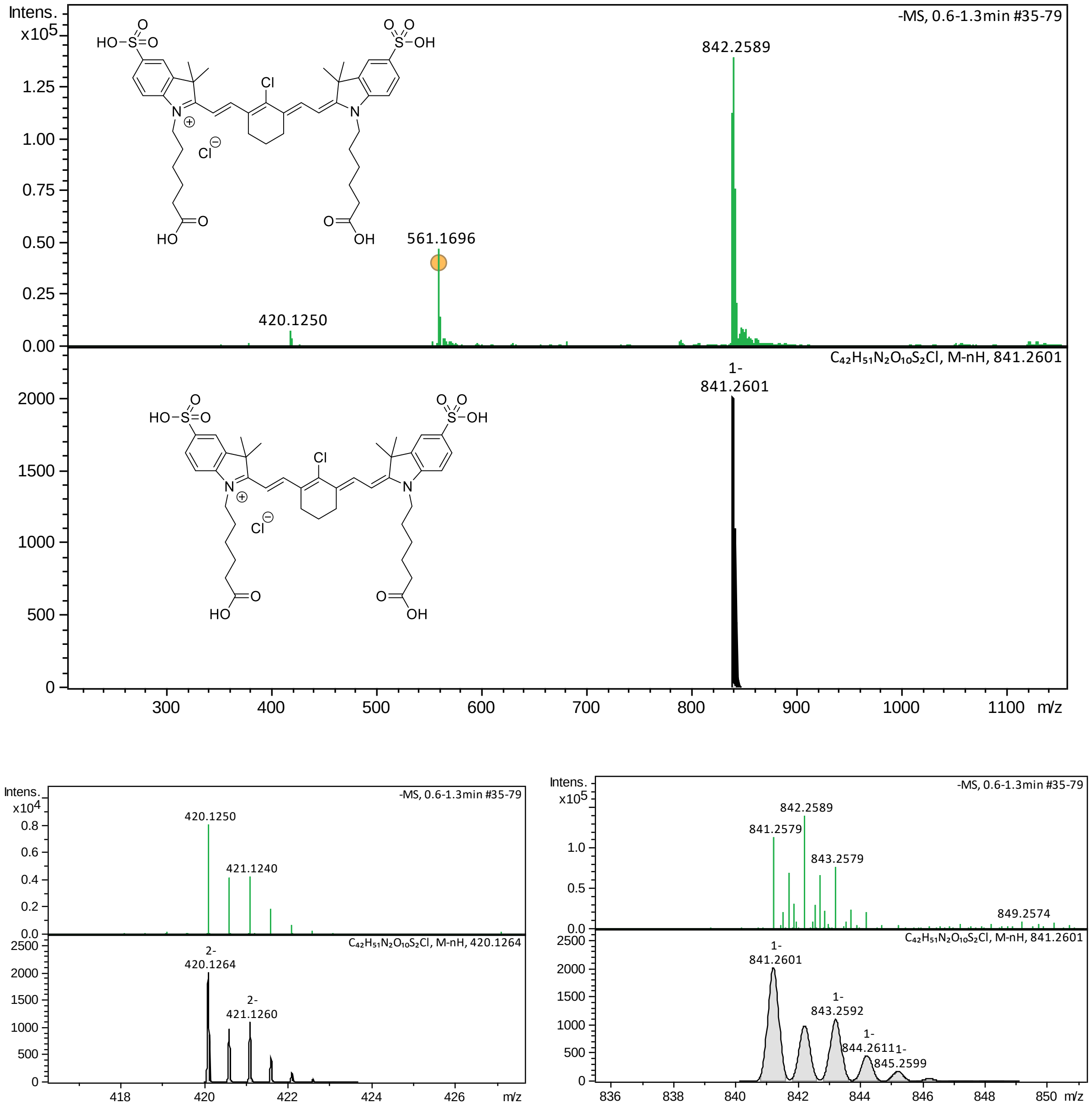

Figure S25. HRMS (ESI, neg. mode, loop injection) of compound **1**.

Compound **2**

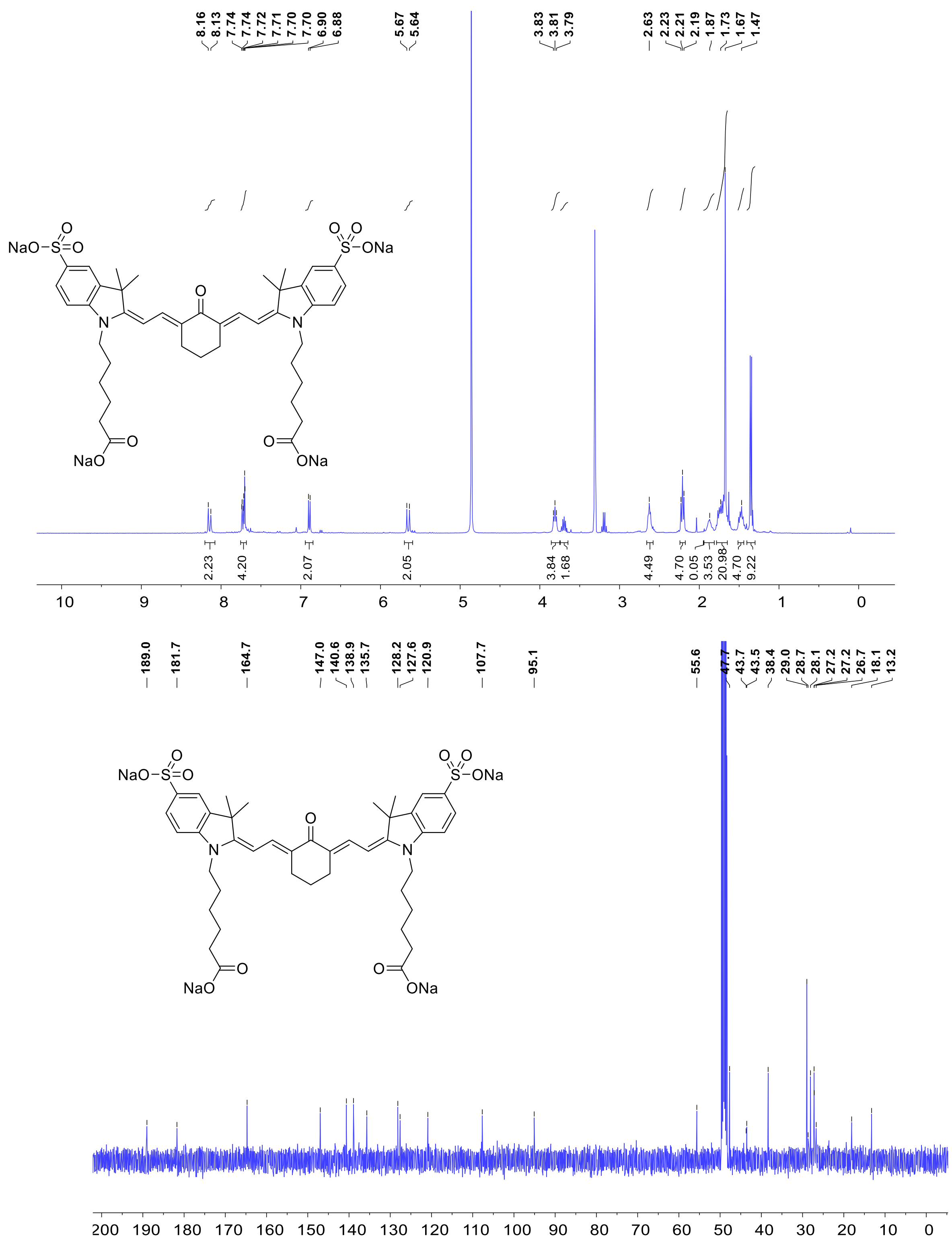

Figure S26. Top: ^{1}H NMR spectrum (400.13 MHz, CD_3OD, 298 K) of compound **2**; Bottom: $^{13}C\{^{1}H\}$ NMR spectrum (100.16 MHz, CD_3OD, 298 K) of compound **2.**

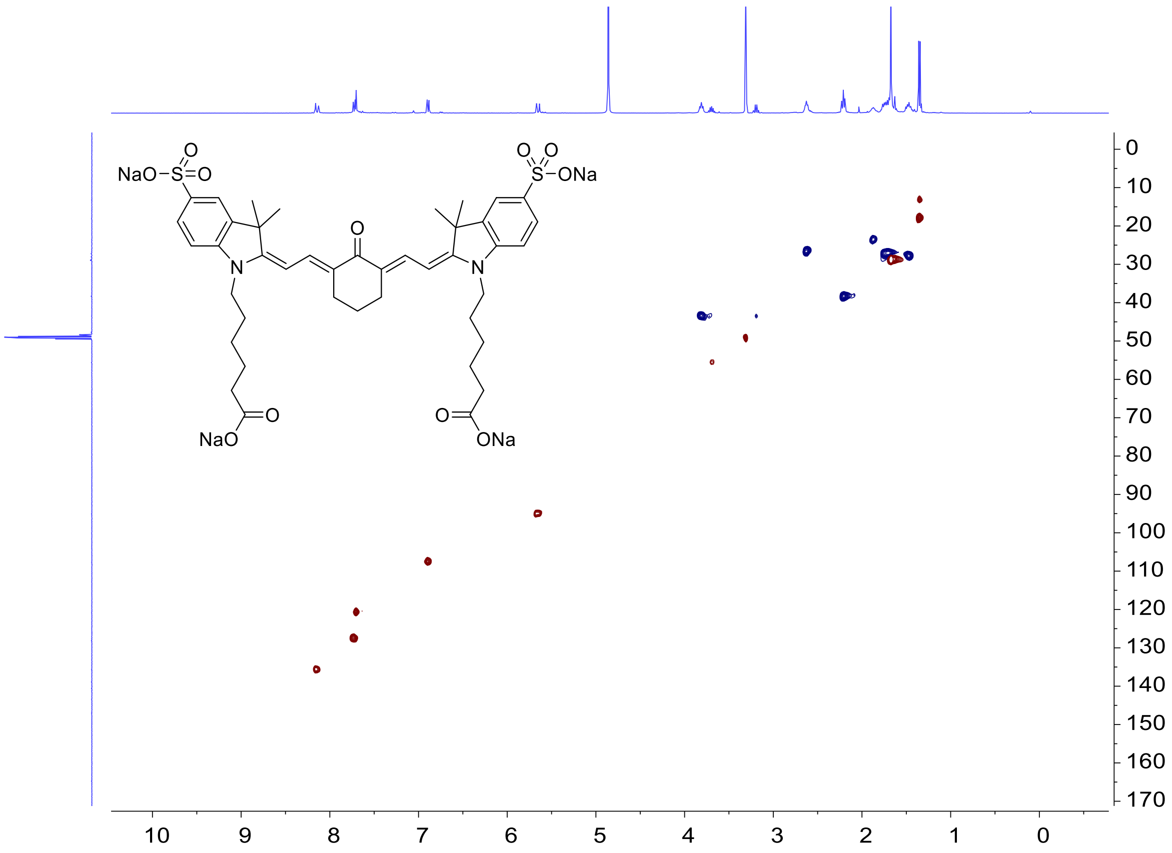

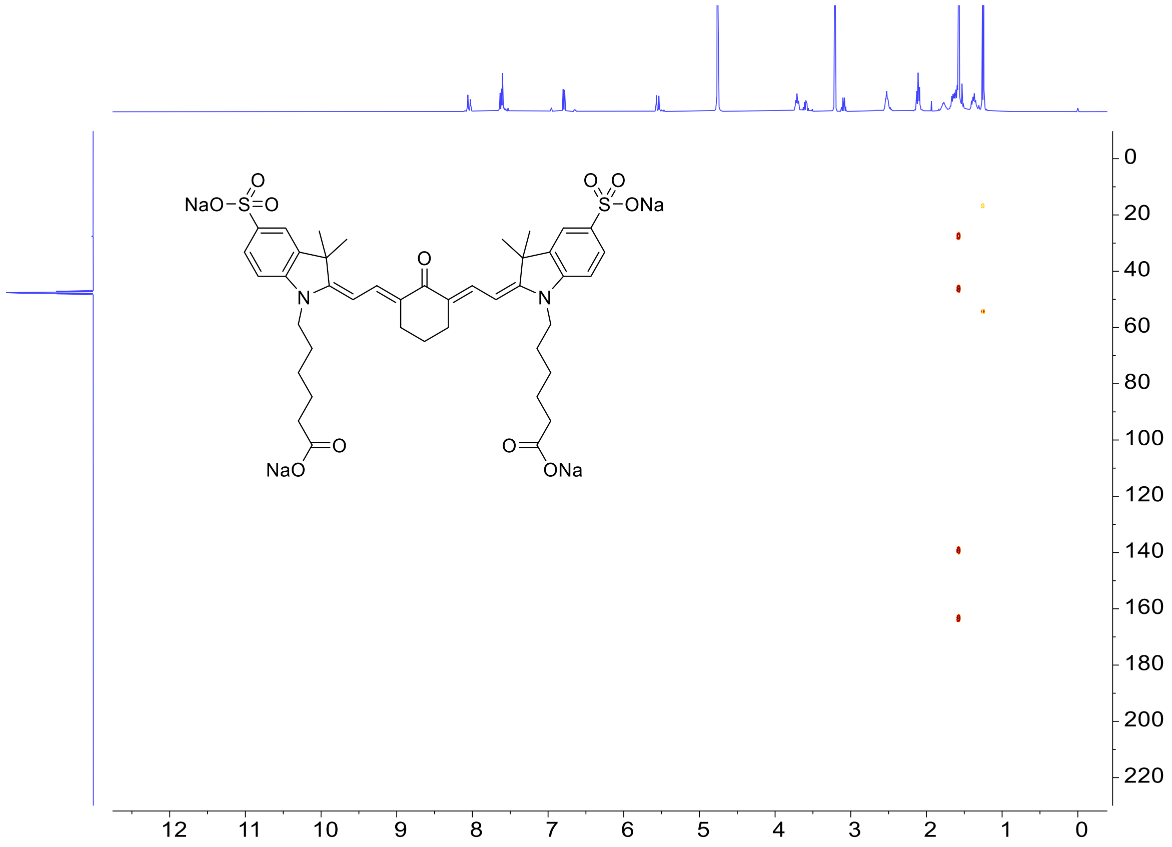

Figure S27. Top: HSQC spectrum (100.16 MHz, CD_3OD, 298 K) of compound **2**;

Bottom: HMBC spectrum (CD_3OD, 298 K) of compound **2**.

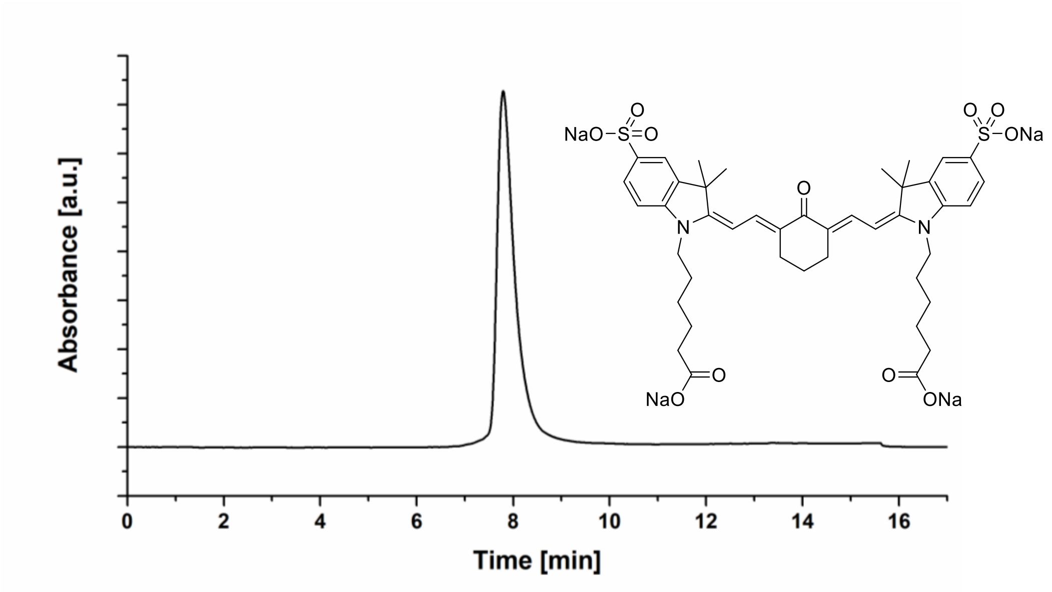

Figure S28. Analytical HPLC trace (560 nm) of compound **2**; Method A.

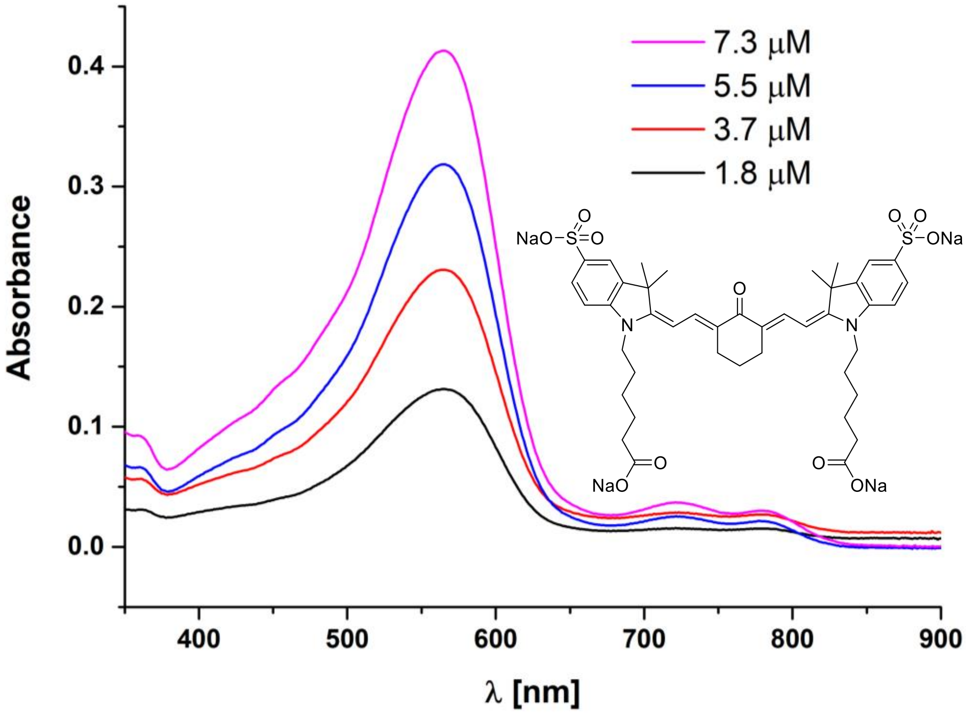

Figure S29. Absorption spectra of compound **2** in PBS at various concentrations.

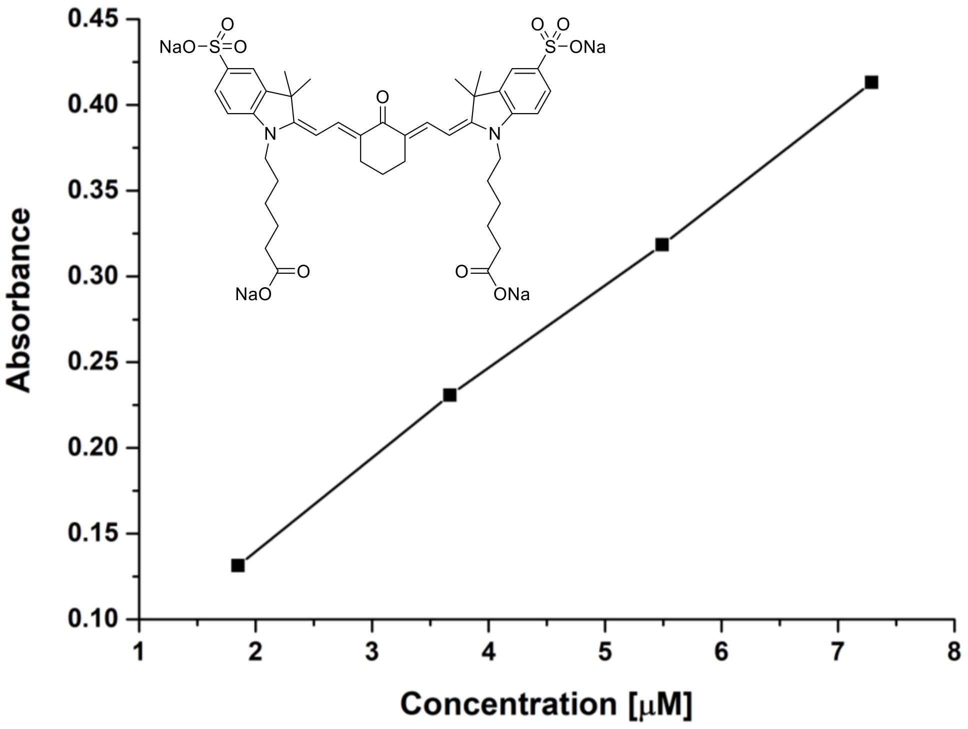

Figure S30. Concentration-dependent absorption of compound **2**. No linear regression was performed because compound **2** appears to not follow the Beer-Lambert law over the concentration range tested in PBS. The molar absorbance of this compound is estimated to be in the range of 58,000 M^{-1}•cm. A reason for the uncertainty in this measurement could be more pronounced aggregation behavior, or protonation equilibria in PBS.

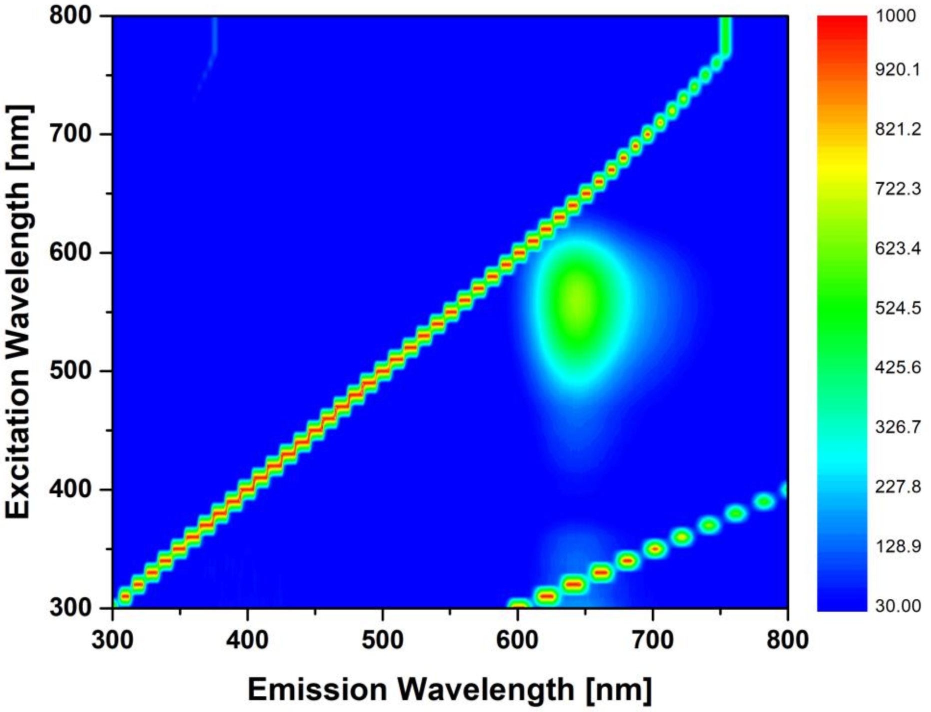

Figure S31. Excitation-Emission map of a solution of compound **2** in PBS (2 µM).

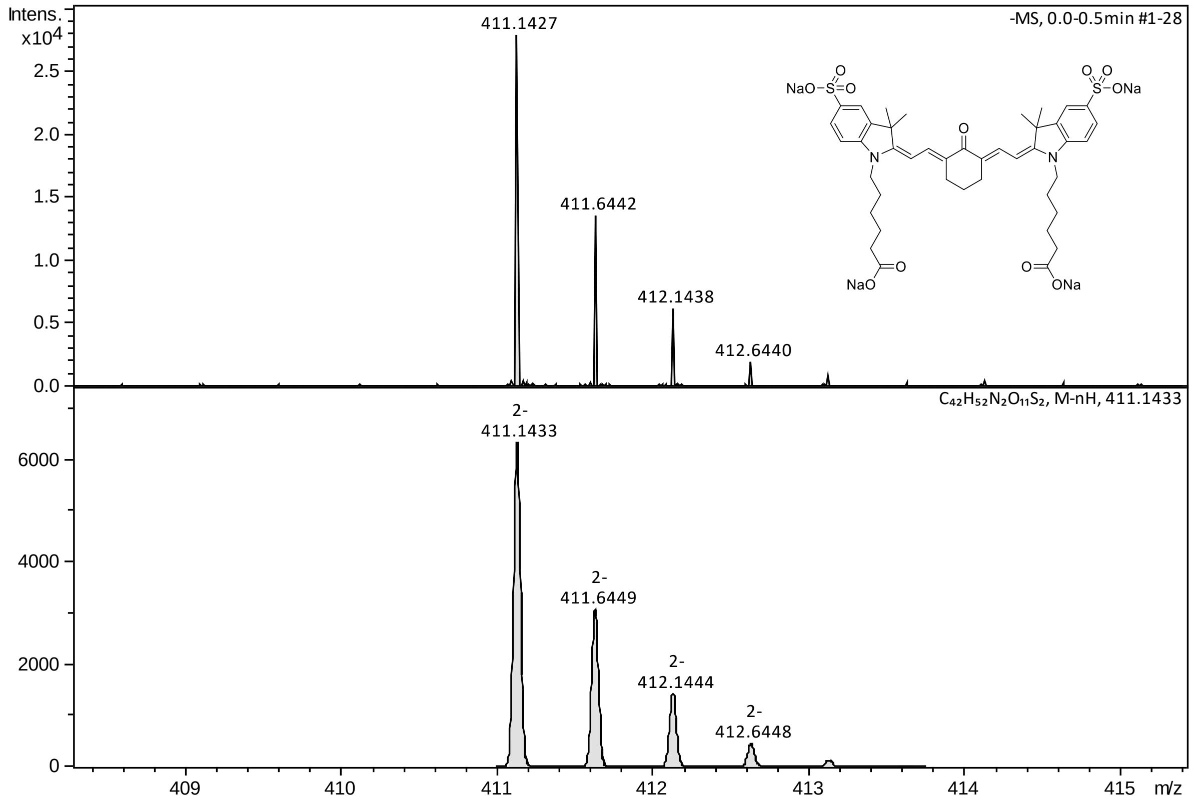

Figure S32. HRMS (neg. mode) of compound **2**.

Compound **4**

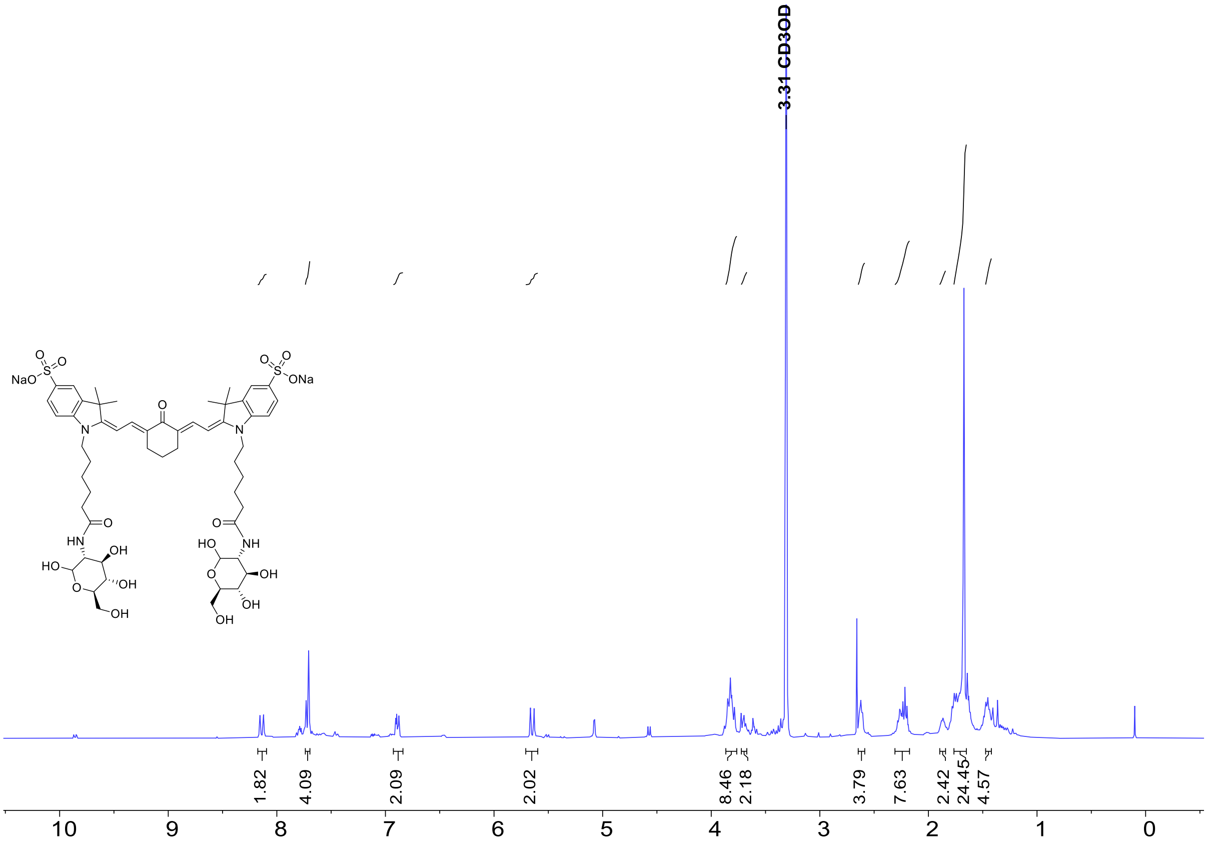

Figure S33. ^{1}H NMR spectrum (400.13 MHz, CD_3OD, 298 K) of compound **4**.

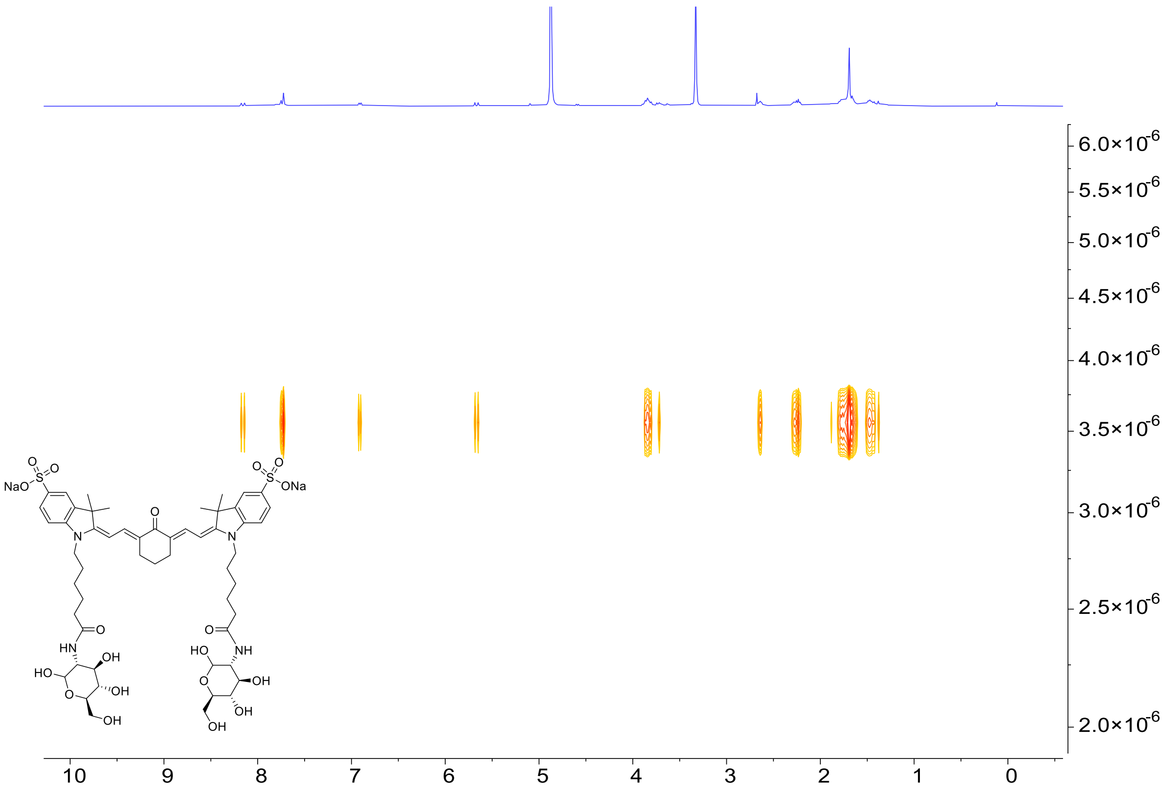

Figure S34. DOSY-NMR spectrum (400.13 MHz, CD_3OD, 298 K) of compound **4**.

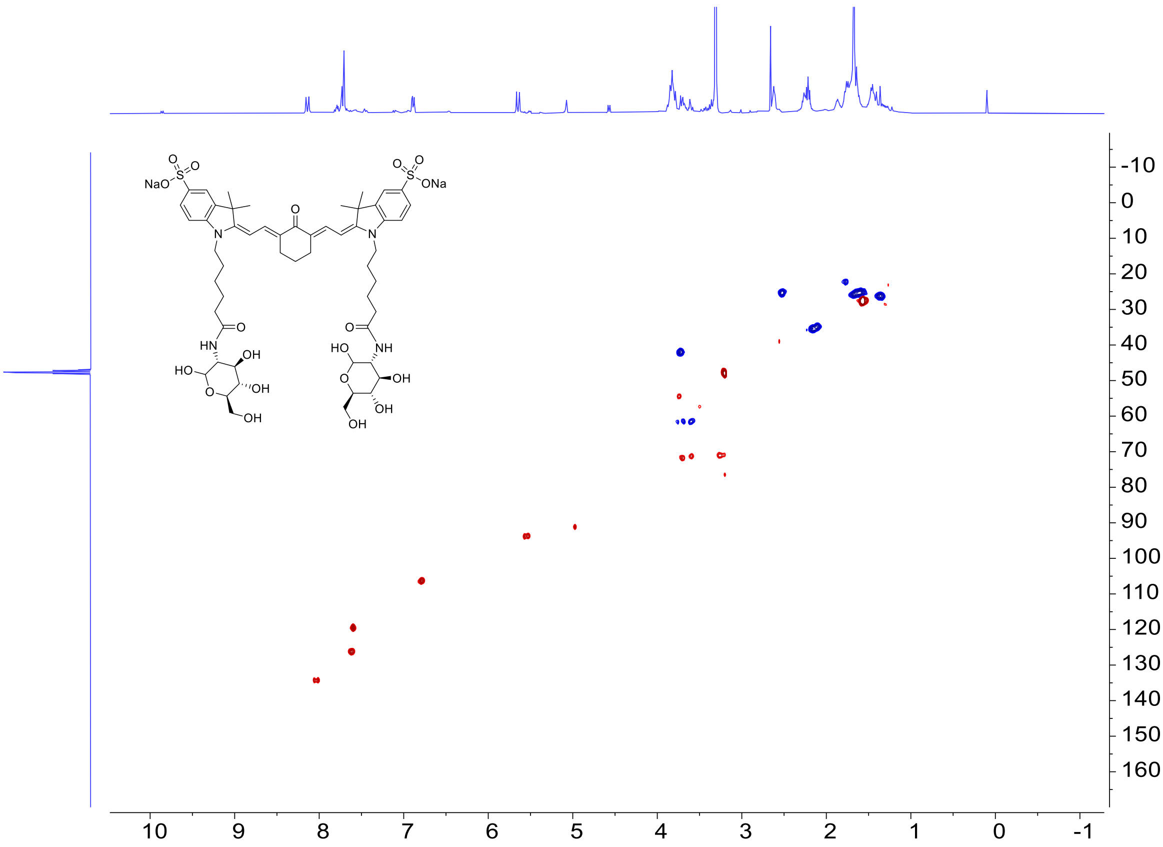

Figure S35. HSQC-NMR spectrum (CD_3OD, 298 K) of compound **4**.

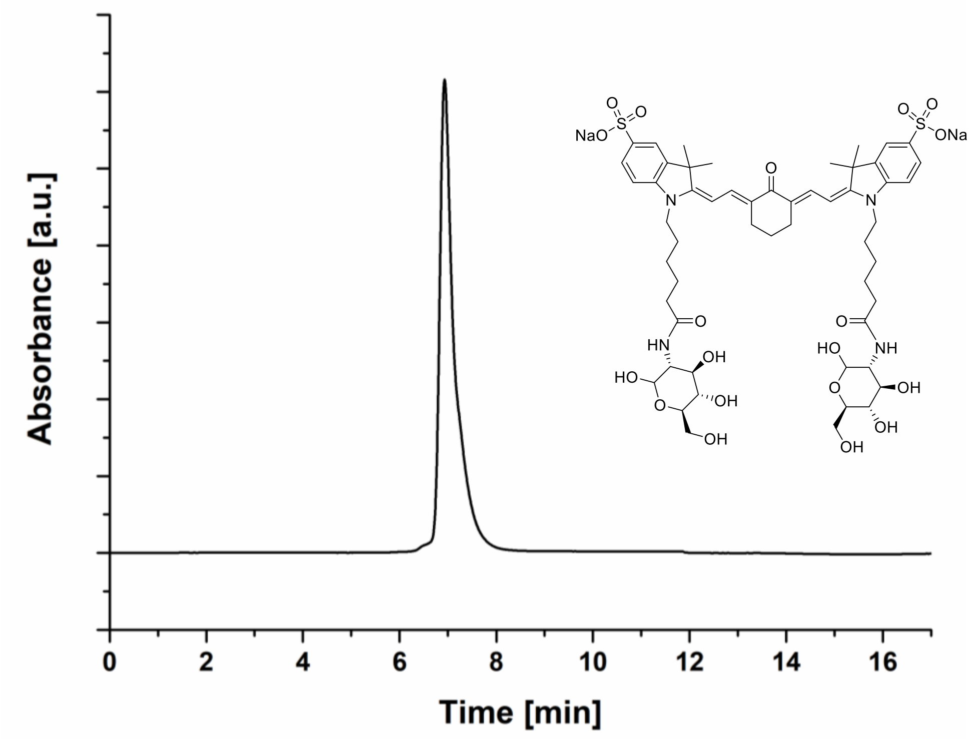

Figure S36. Analytical HPLC trace (560 nm) of compound **4**; Method A

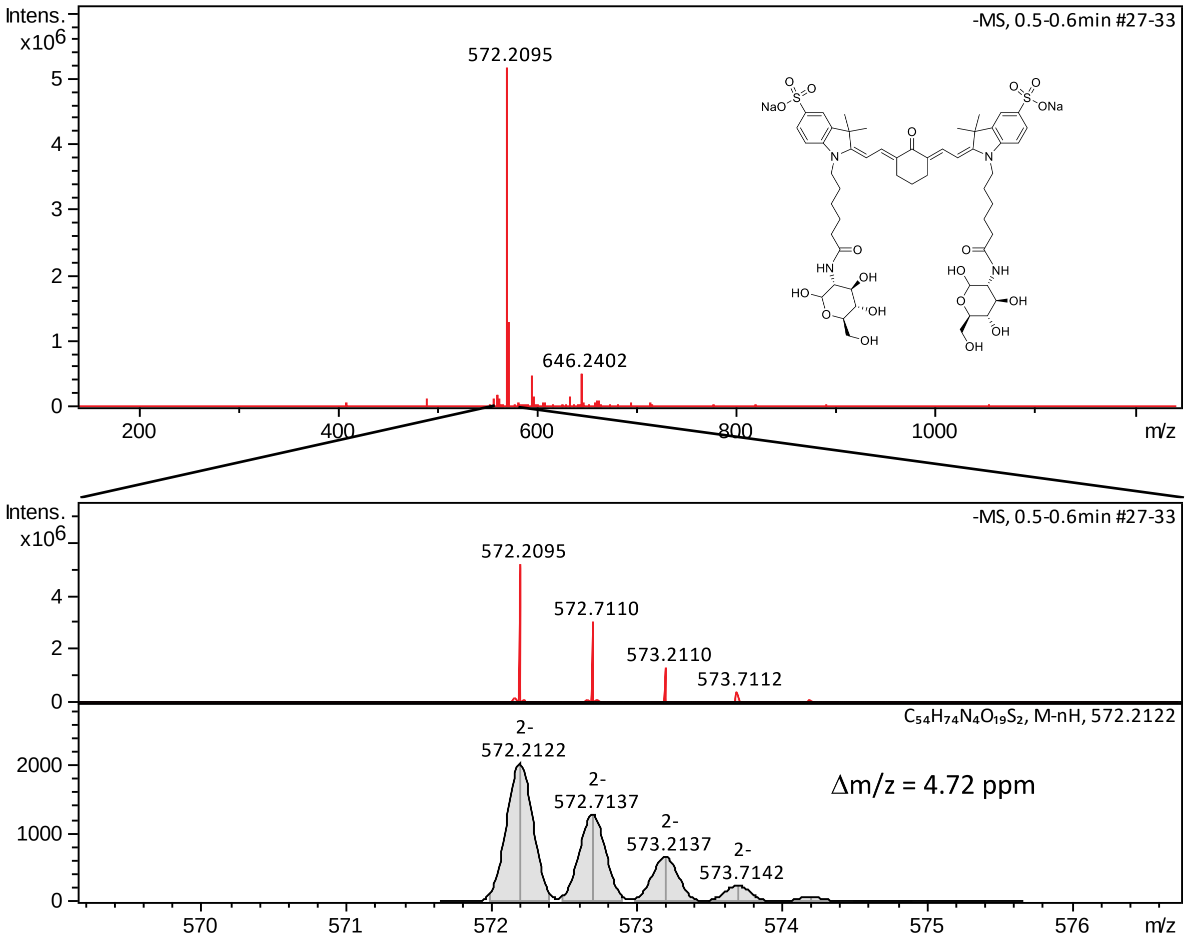

Figure S37. HRMS (ESI, neg. mode, loop injection) of compound **4**.

Characterization data for **5**

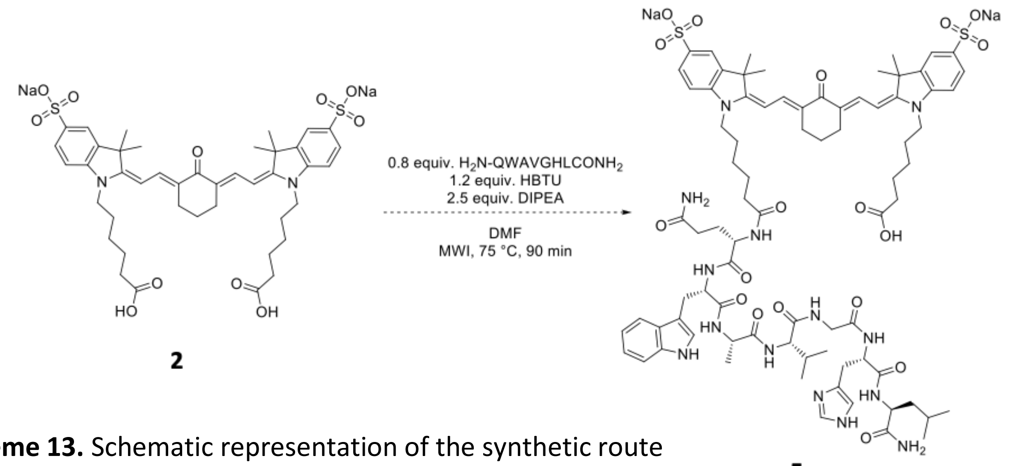

Scheme 13. Schematic representation of the synthetic route to Compound **5.**

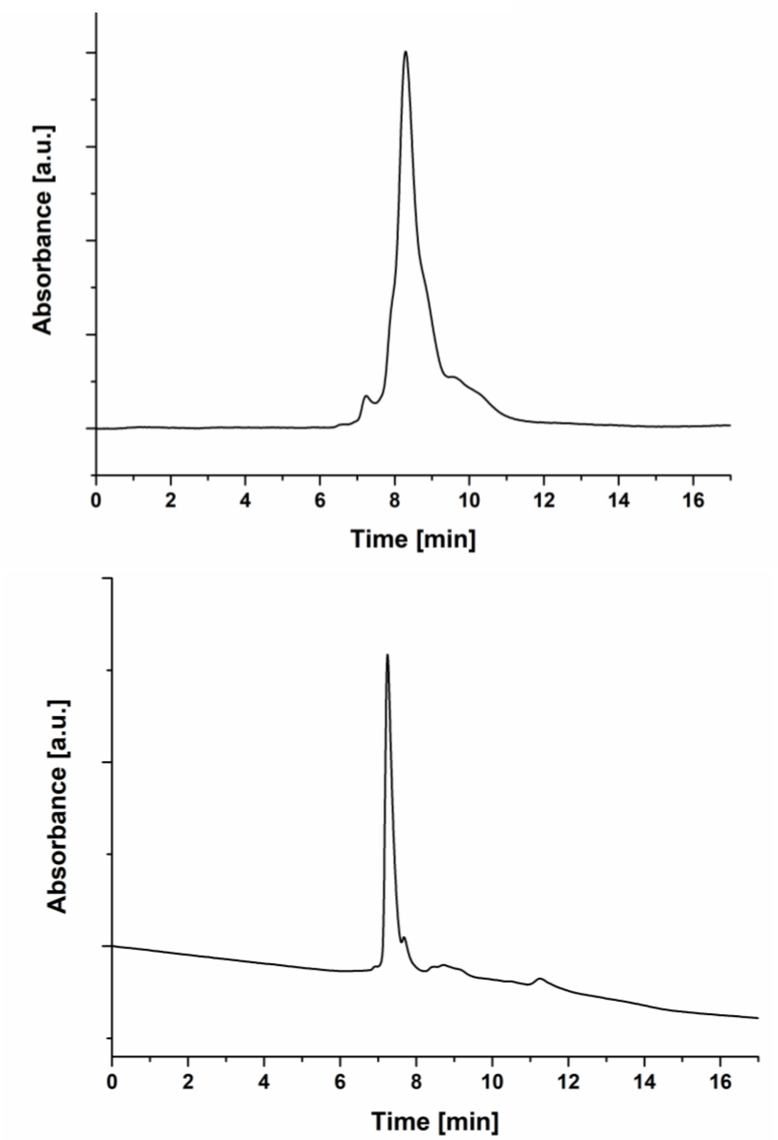

Figure S38. Analytical HPLC trace (560 nm, Top) of compound **5** and a comparison with the corresponding free [7, 13]Bombesin (280 nm, Bottom); Method A.

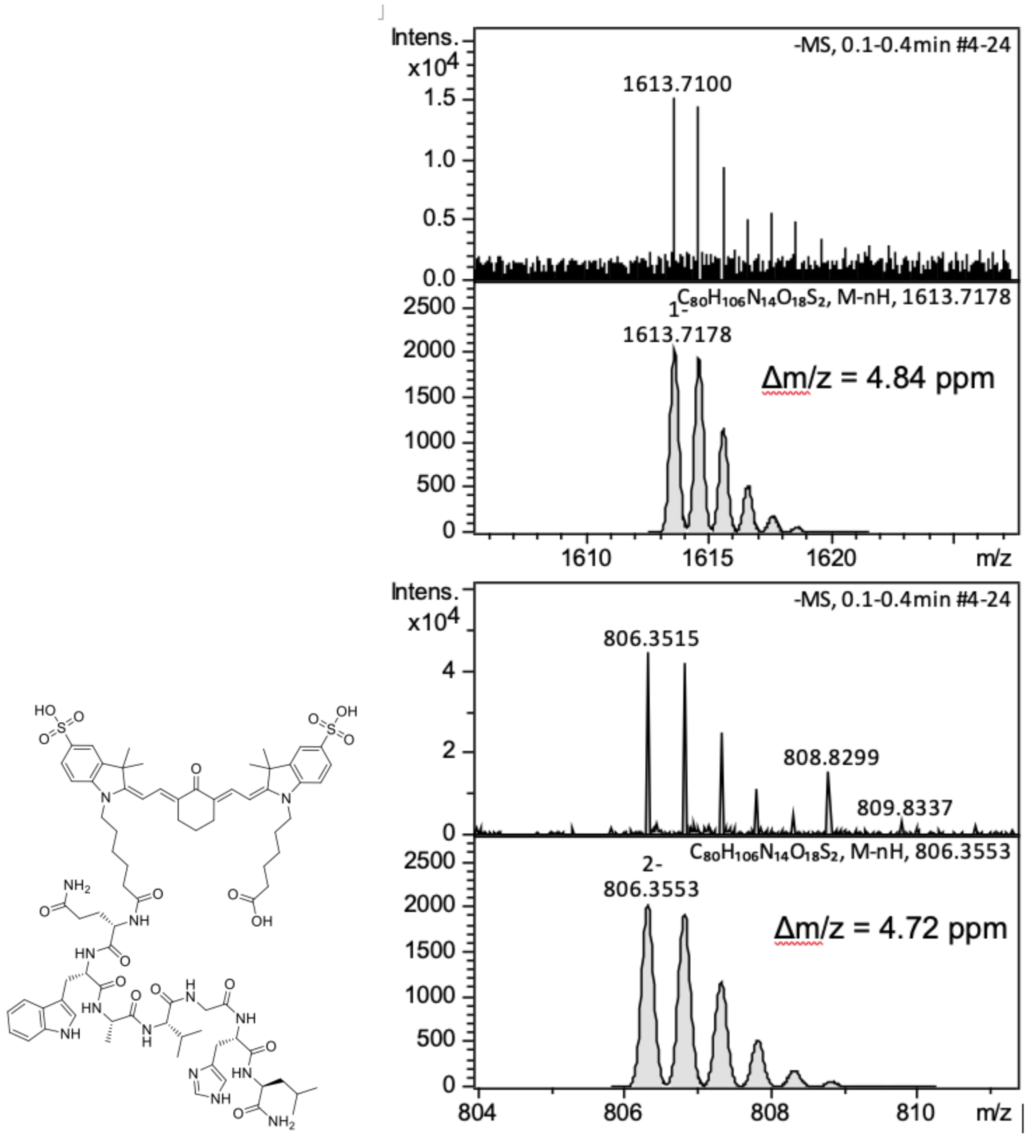

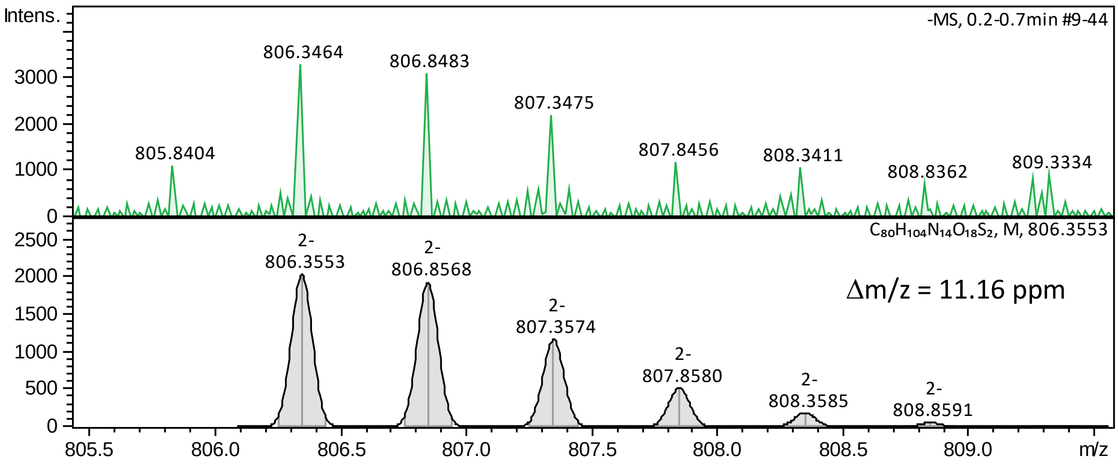

Figure S39. The ESI-MS of the purified compound **5**, along with the simulated isotope pattern, showing signals corresponding to the mono-anionic $[M\text{-}H]^-$ and di-anionic $[M\text{-}2H]^{2-}$ species. Further detail of the HRMS (ESI, neg. mode, loop injection) of compound **5**.

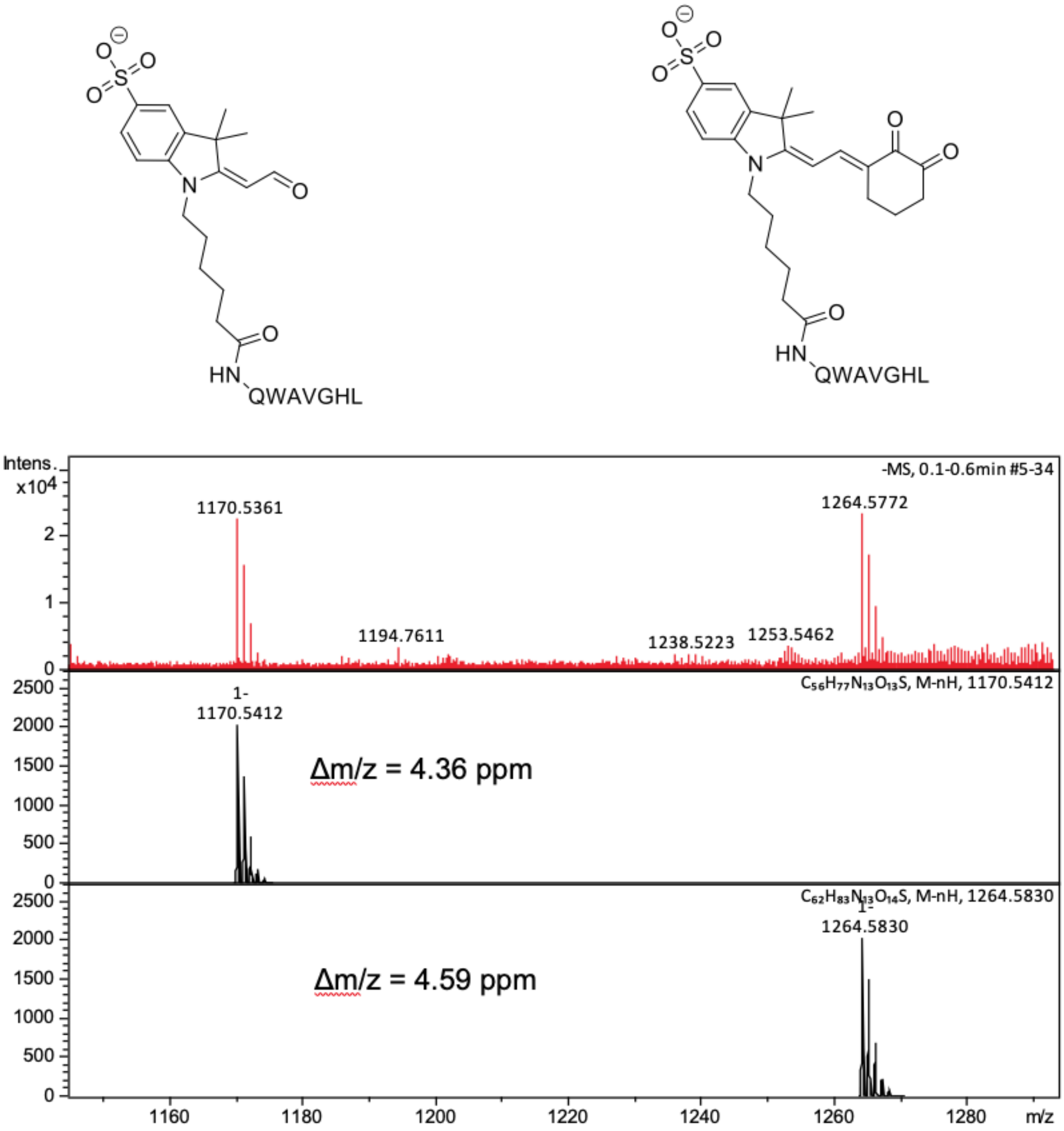

Figure 40. Proposed structures of fragments observed in the ESI-MS of compound **5**, along with the detail of the spectrum showing signals corresponding to these.

In Vitro Assays

Biological activity of investigated dyes using standard metabolic inhibition assays

Generally, cyanine dyes are thought to be biocompatible and do not induce considerable side-effects beyond local reactions at the injection site in clinical trials. Here, the inhibition of cell metabolism was investigated in healthy skin fibroblast (FEK4) and cancerous cell lines (PC3, a bone metastasis of a stage IV prostate cancer, and DU145, isolated from a brain metastasis of a patient with prostate cancer) using standard MTT assays with the agent 3-(4,5-dimethylthiazol-2-yl)-2,5-diphenyltetrazolium bromide. The unfunctionalized, sulfonated dye **1** did not appear to affect cellular metabolism or growth in healthy skin-fibroblast and in prostate cancer cell lines up to a concentration of 250 μM for 24 h, approximately 25-50 times more than would be used in typical microscopy experiments. The glutathione conjugate **3**, likewise, did not show any statistically significant inhibition of cell metabolism over time-periods of 30 minutes or 24 hours. The non-sulfonated compound **MIH-148** showed inhibition of cell metabolism at higher concentration after 24 hours of incubation in PC3.

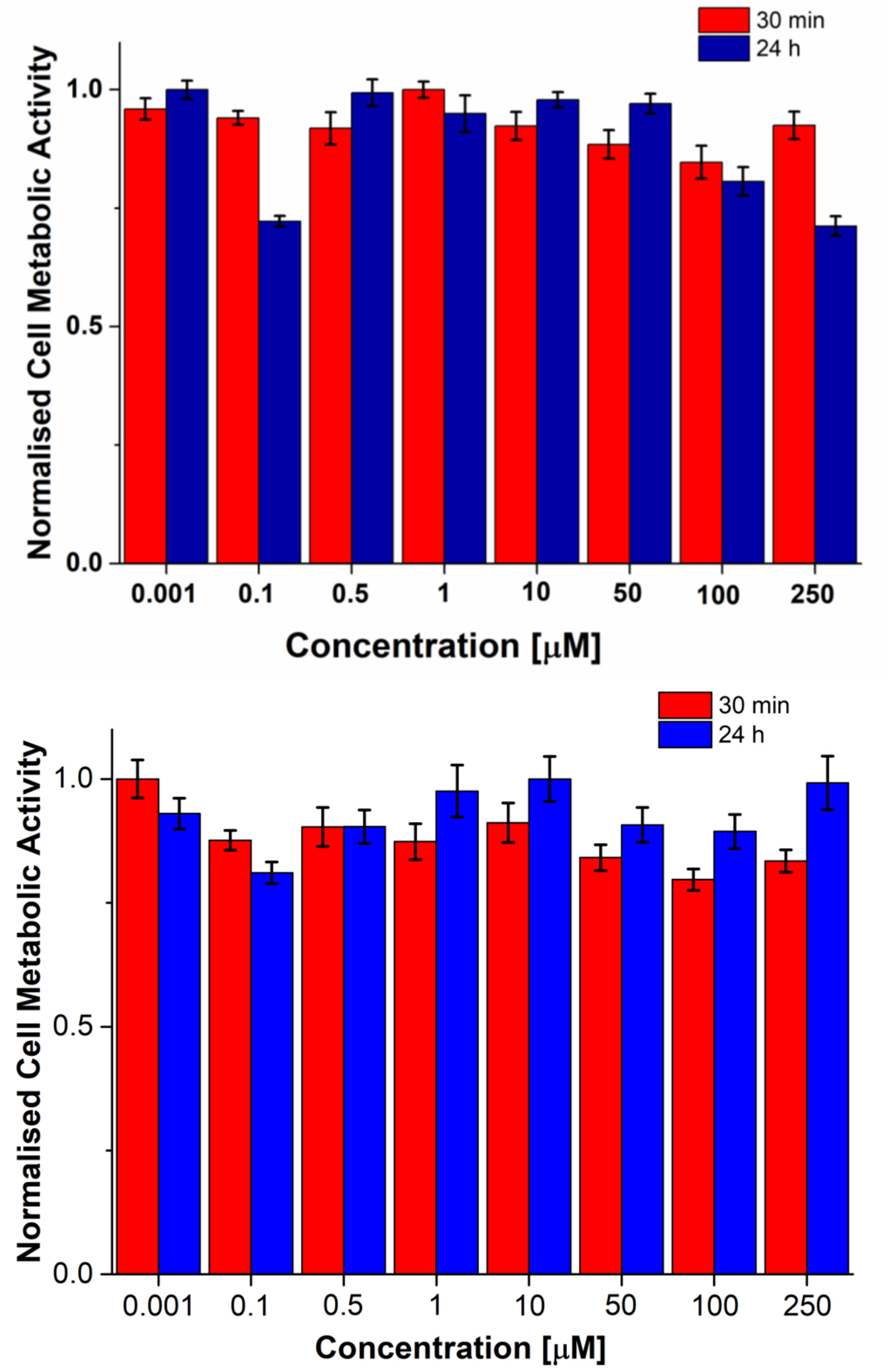

Figure 41. MTT-Assays (30 min, 24 hours) of compound **1** in PC3 (top) or FEK4 (bottom) cells, showing no statistically significant inhibition of cellular metabolism in the concentration range tested

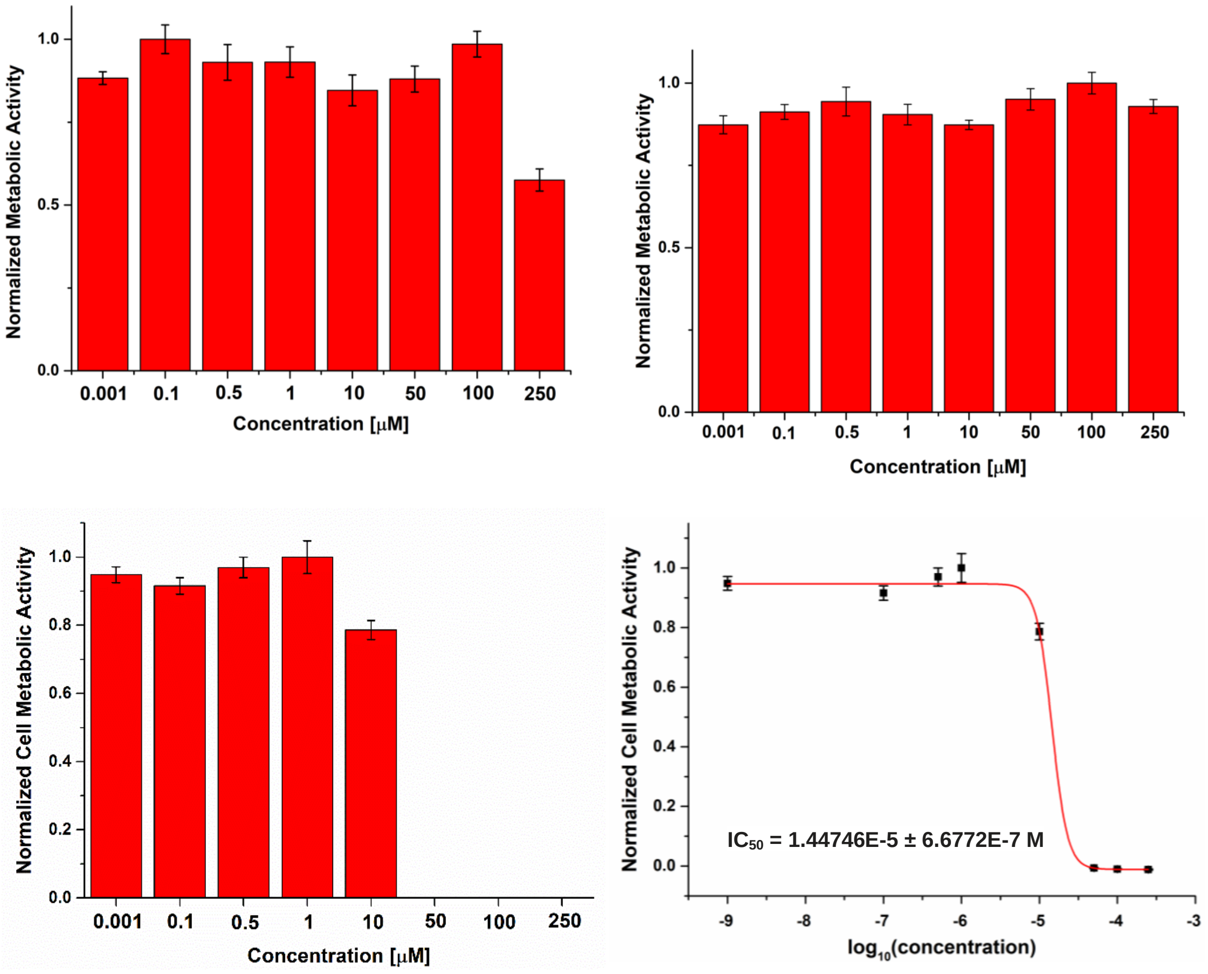

Figure S42. Top Row: Results of MTT assays in PC3 cells after 24 hours of incubation with various concentrations of **1** (left) and **2** (right); Bottom Row: Results of MTT assays in PC3 cells after 24 hours of incubation with various concentrations of **MHI-148**.

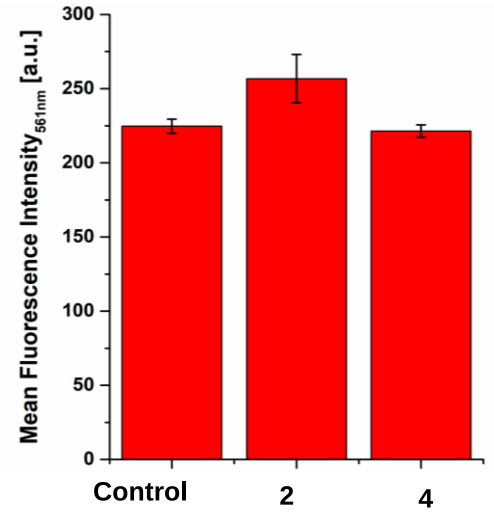

Figure S43. Statistical analysis of uptake of **2** and **4** (10 μM, 1 h) in PC3 cells, measured across two fields of view (FOV) in six different cells per FOV from images recorded and shown in Figure S44, below

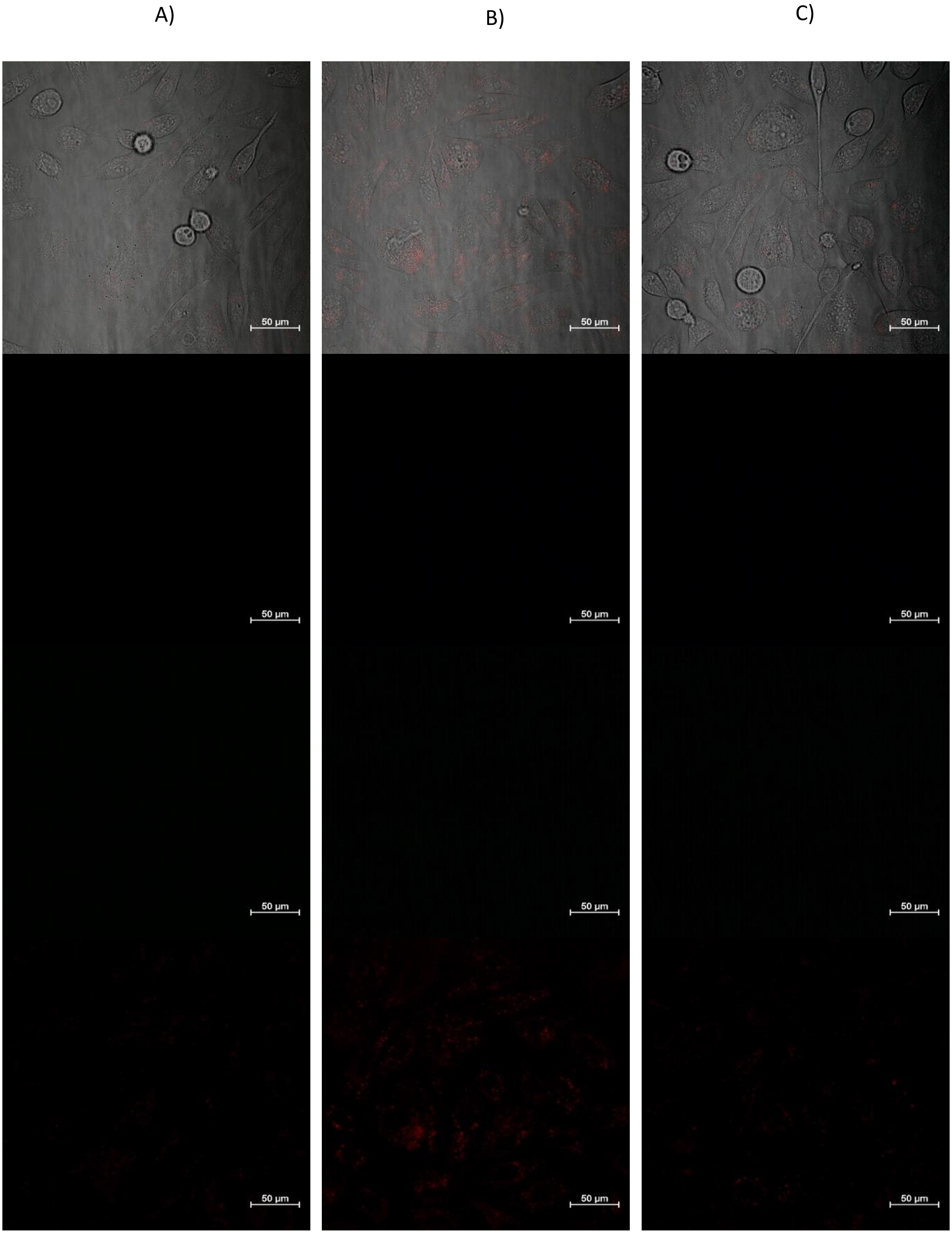

Figure S44. Confocal microscopy in PC3 cell lines using compounds **2** and **4** (10 µM, 1 h incubation). A) untreated control; B) incubation with **2**; C) incubation with **4**. From top to bottom: λ_{exc} = 405 nm, 488 nm, 561 nm, overlay with brightfield.

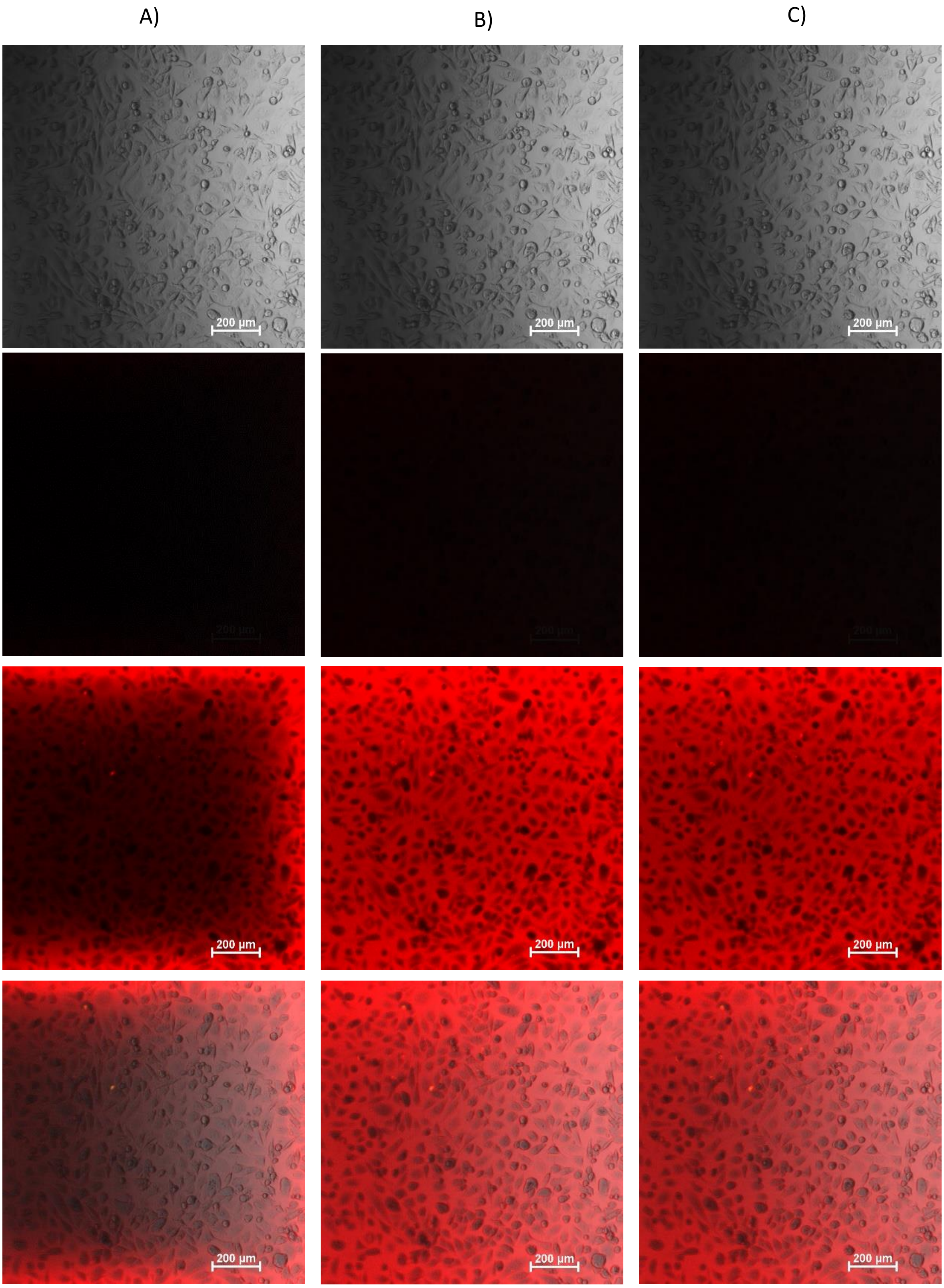

Figure S45. Uptake studies in PC3 cell lines using compounds **2** (10 µM). A) t = 0 min; B) t = 10 min C) t = 20 min. From top to bottom: Brightfield, green channel (λ_{exc} = 488 nm), red channel (λ_{exc} = 561 nm), overlay with brightfield.

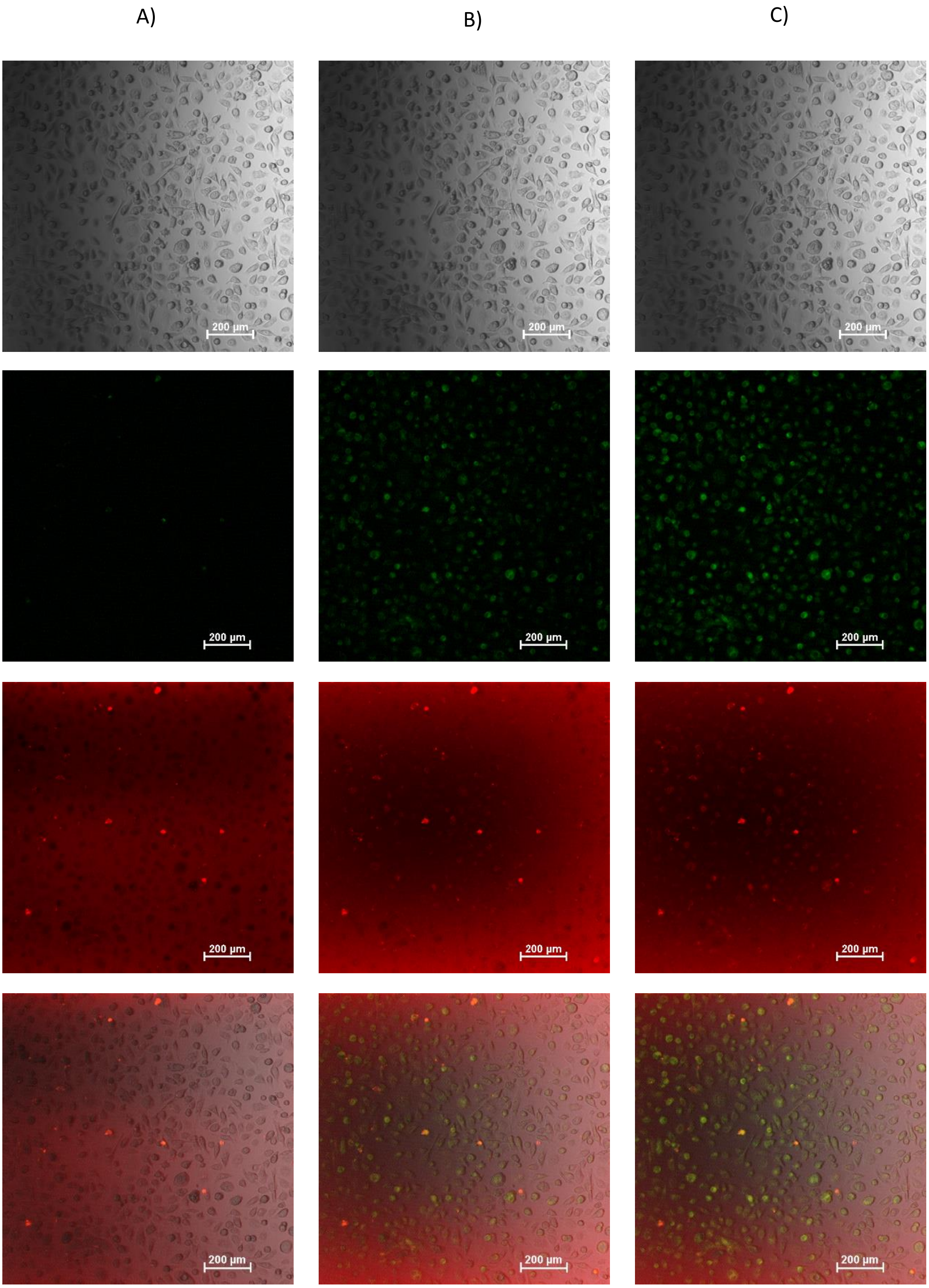

Figure S46. Uptake studies in PC3 cell lines with **MHI-148-O** (10 μM, 37°C). A) t = 0 min; B) t = 10 min C) t = 20 min. From top to bottom: Brightfield, green channel (λ_{exc} = 488 nm), red channel (λ_{exc} = 561 nm), overlay with brightfield.

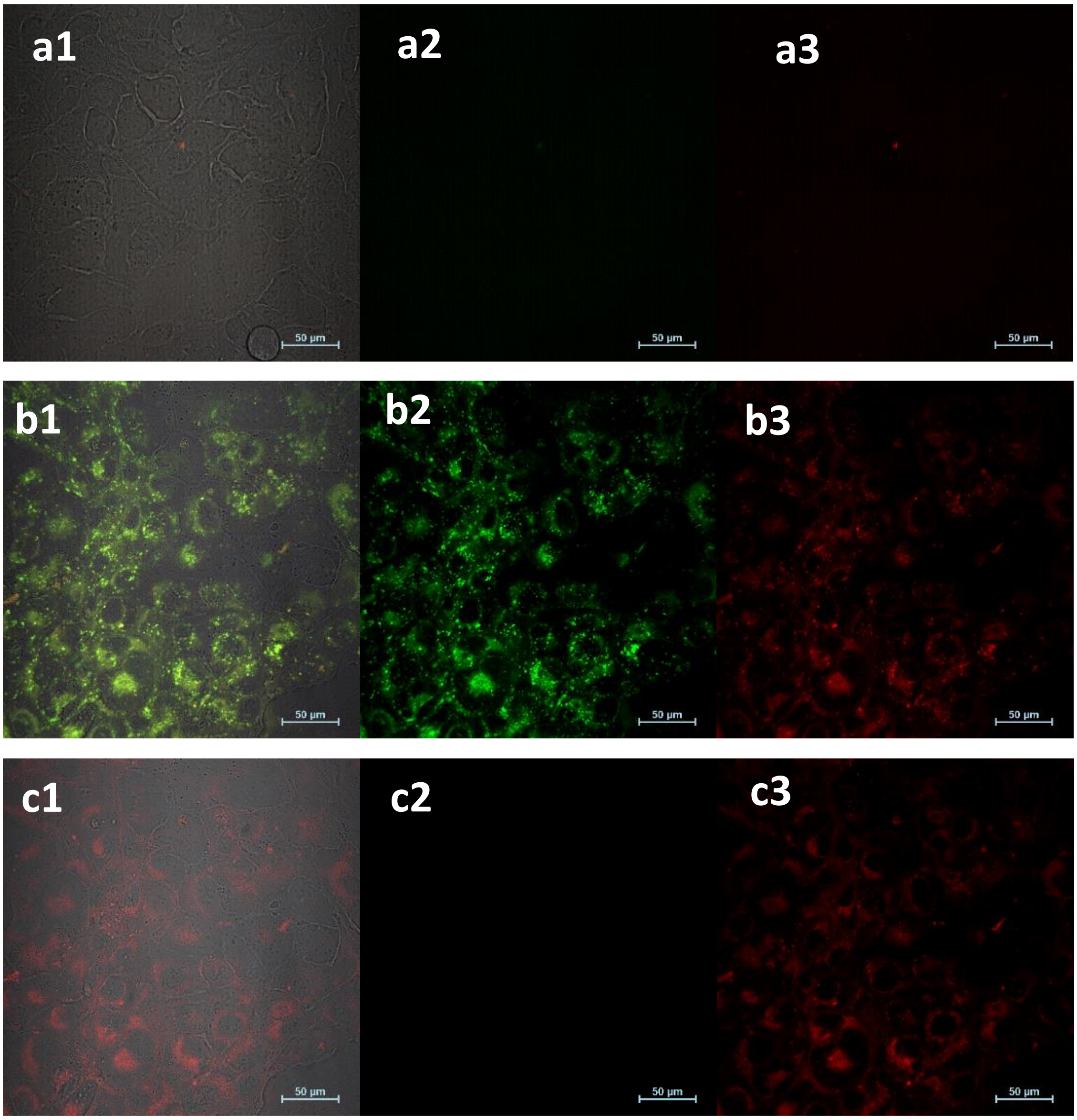

Figure S47. Confocal Microscopy Images of DU-145 cells incubated with either Compound **2** (a1-a3) or the corresponding oxo-form of MHI-148 (b1-b3, c1-c3). Cells were incubated with the respective compound (10 µM) for 20 min at 37 °C. Images of the same field of view are depicted, excited either at 488 nm (b1-b3) or 561 nm (c1-c3). From left to right: overlay with brightfield (1), green emission channel (2), red emission channel (3). Scalebar 50 μm.

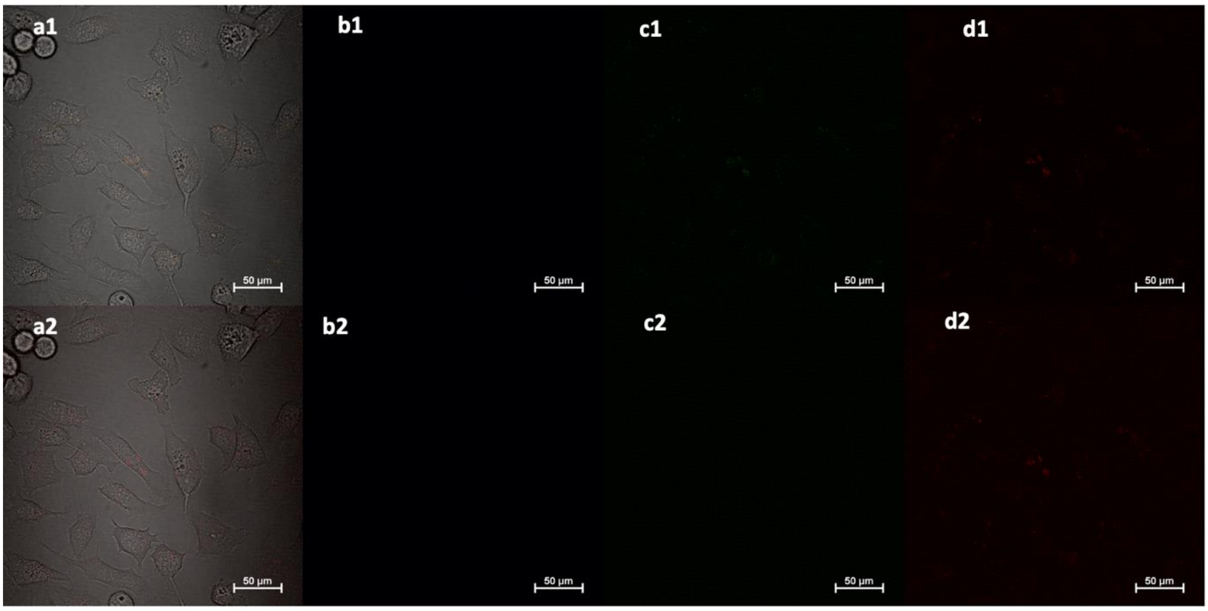

Figure S48. Confocal Microscopy Images of PC-3 cells as control, incubation time 2 h at 37 °C. Images of the same field of view are depicted, excited either at 488 nm (a1-d1) or 561 nm (a2-c3). From left to right: overlay with DIC (a1; a2), blue emission channel (b1, b2), green emission channel (c1, c2), red emission channel (d1,d2). Scalebar 50 *μ*m.

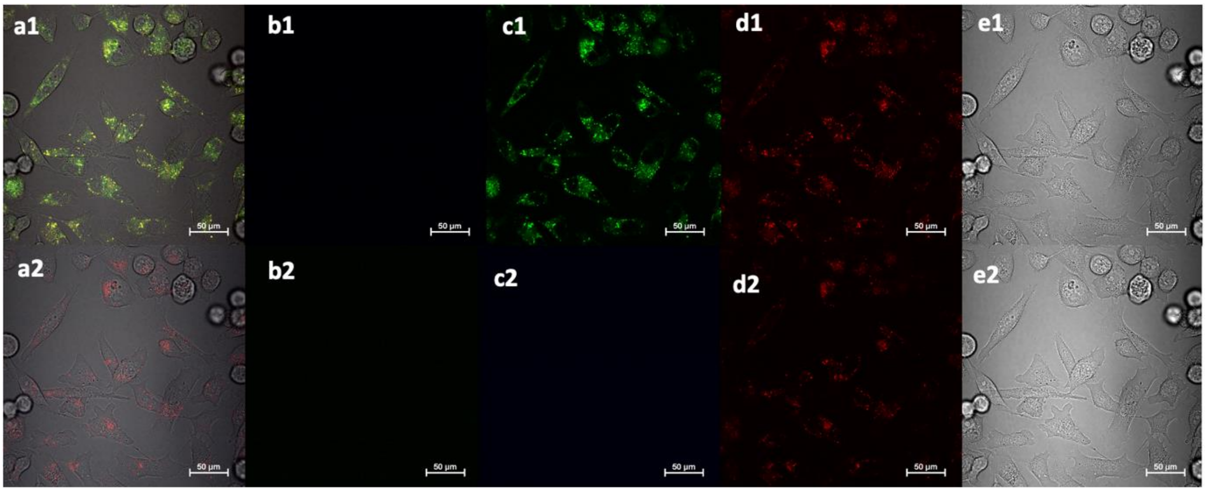

Figure S49. Confocal Microscopy Images of PC3 cells incubated with Compound **2** (100 μM) for 2 h at 37°C. To highlight the dual-emission behavior of this compound, images of the same field of view are depicted, excited either at 488 nm (a1-e1) or 561 nm (a2-e2). From left to right: overlay with DIC (1), blue emission channel (2), green emission channel (3), red emission channel (4) and DIC (5). Scalebar 50 *μ*m.

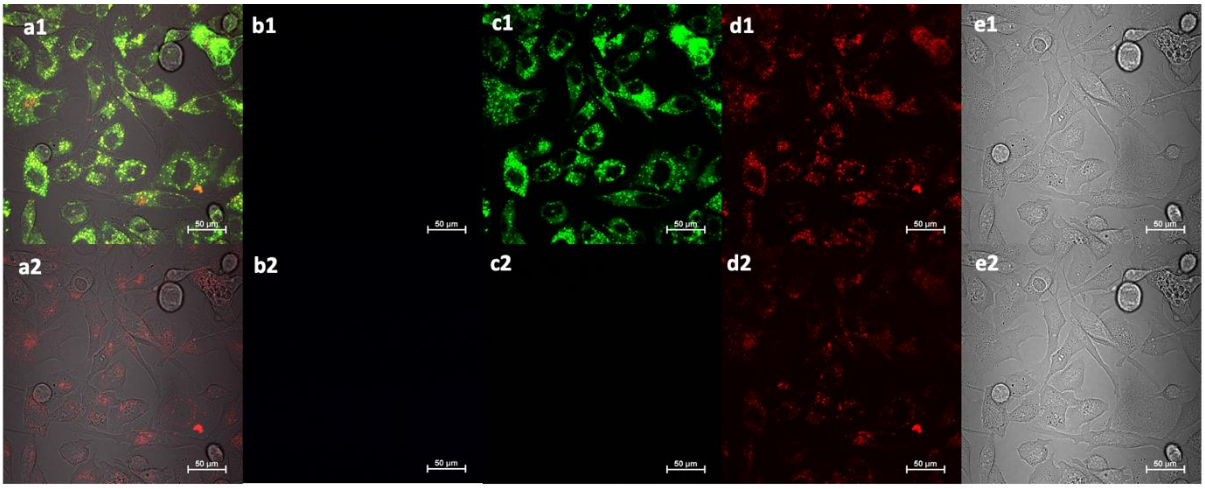

Figure S50. Confocal Microscopy Images of PC3 cells incubated with Compound **4** (100 µM) for 2 h at 37 °C. To highlight the dual-emission behavior of this compound, images of the same field of view are depicted, excited either at 488 nm (a1-e1) or 561 nm (a2-e2). From left to right: overlay with DIC (1), blue emission channel (2), green emission channel (3), red emission channel (4) and DIC (5). Scalebar 50 *µ*m.

Optical Properties in Biological Media

To investigate the stability and potential changes to the optical properties of **2** and **MHI-148-O** in biological media, a sample of the dye was dissolved in FBS, and the UV/VIS and fluorescence spectra were measured. The results indicate aggregation to proteins, particularly in the case of **MHI-148-O**, causing intense fluorescence in a further, blue-shifted region of the spectrum. This is likely due to interaction with hydrophobic surfaces of proteins or other biomacromolecules. Such interactions are not observed for the parent dye **1**. The charge difference between the meso-chloride dyes and the keto-polymethines may explain this difference.

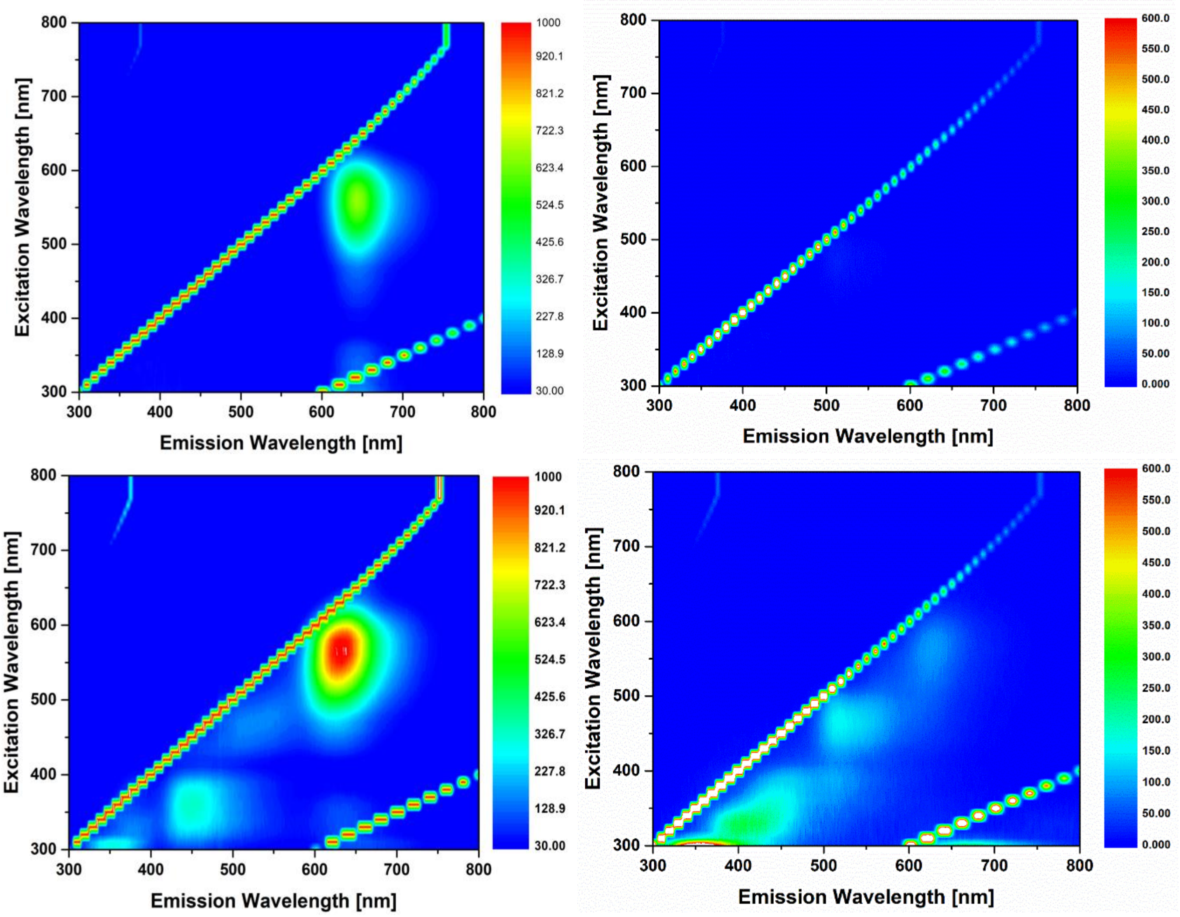

Figure S51. TOP Row: EEM maps at 2 μM conc. for **2** (left) and **MHI-148-O** (right) in PBS; Bottom Row: EEM maps of **2** (left) and **MHI-148-O** (right) at 2 μM conc 10% Serum Medium (10% FBS in RPMI, bottom).

DFT and TD DFT Calculations

Theoretical modelling was performed at the BLYP/TZP level of theory, using the ADF suite.[8, 9] Initially, simplified structures featuring methyl groups, rather than hexanoic acid chains, were created using the graphical user interface, and geometries optimized in the gas phase before analytical frequencies were calculated.[10, 11] These structures are given the suffix -Me to differentiate them from the synthesized compounds. **Figure S52** shows the optimized geometries of compounds denoted **1-Me** and **2-Me**. As would be expected, the C-O bond length in the *meso*-position of **2-Me** appears to be in the range of typical double-bonds (1.253 Å). Also, the angle of the central C-C-C motif in the *meso*-position reduces from 125.3° for **1-Me** to 118.5° for **2-Me**.

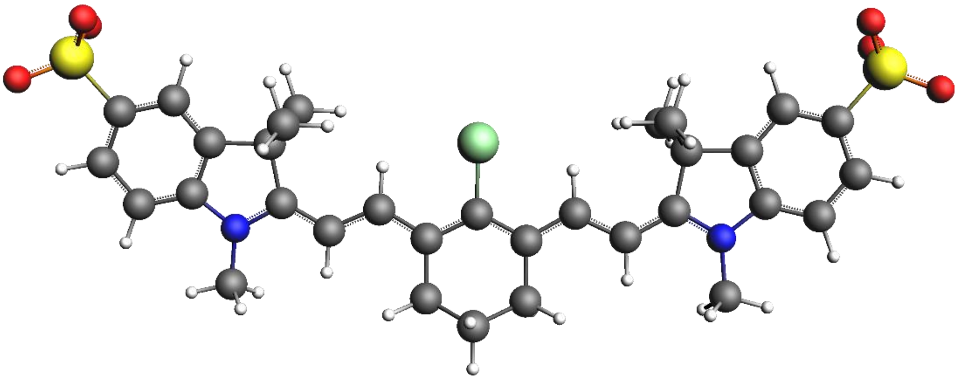

1-Me

Enthalpy = - 42015.98 kJ/mol

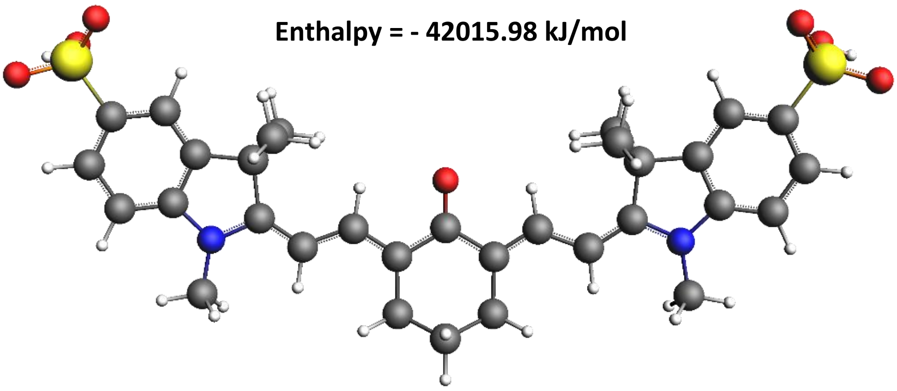

2-Me

Enthalpy = - 42955.70 kJ/mol

Figure S52. Optimized Geometries for **1-Me** (above) and **2-Me** (below) (BLYP/TZP, gas phase). Color code: Grey = C, Blue = N, Red = O, Yellow = S, Green = Cl.

If no imaginary frequencies were found the continuous solvation model COSMO (conductor-like screening model) was applied (simulating water, dielectric constant ε = 78.39, radius of the rigid sphere solvent molecules = 1.93 Å) and geometry optimizations and frequency calculations repeated.[12] Although results in the gas phase did not contain

imaginary frequencies, those applying the COSMO model usually contained several near-zero imaginary frequencies. Rescanning of these frequencies using numerical methods with finite replacement resolved this issue. **Figure S53** shows the reconstructed COSMO surfaces obtained for **1-Me** and **2-Me**.

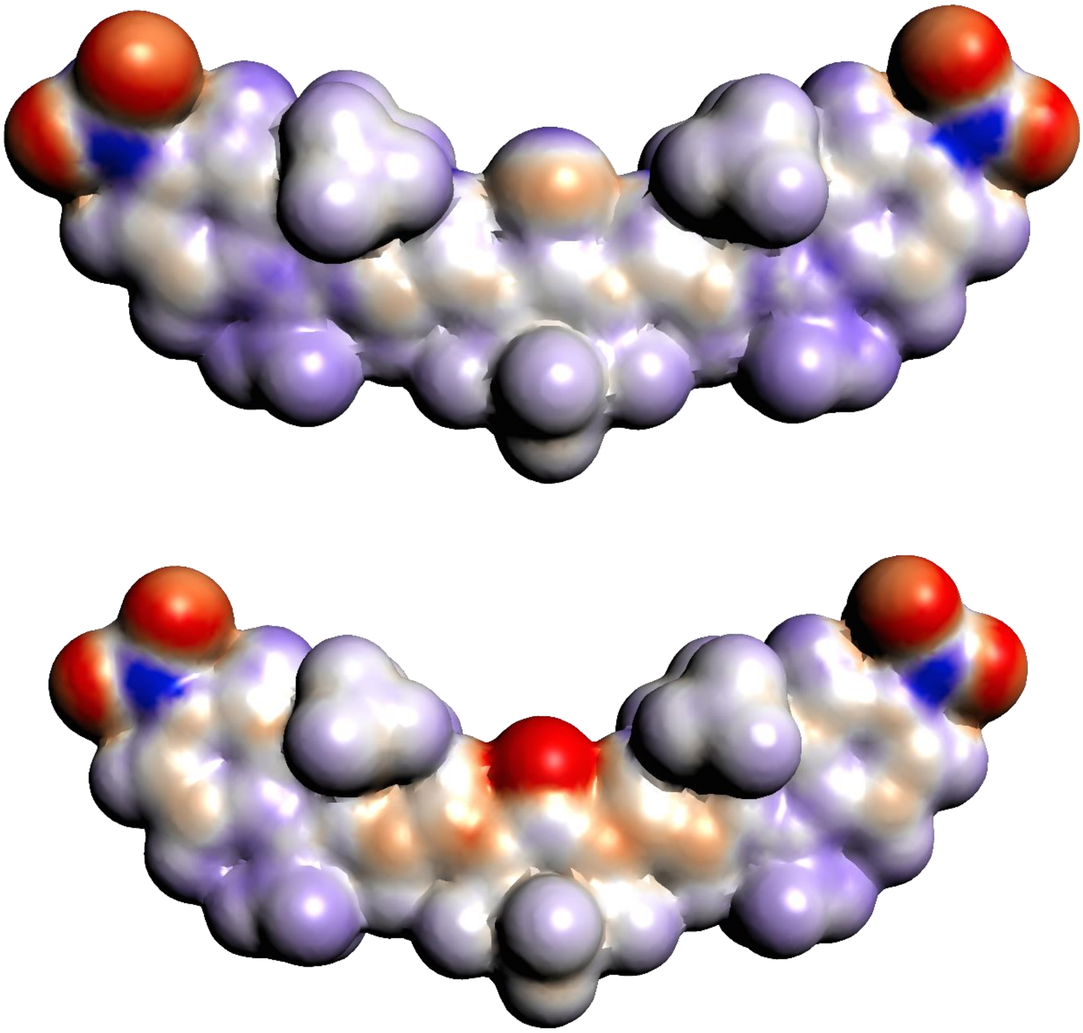

Figure S53. Reconstructed COSMO-surfaces of **1-Me** and **2-Me** (BLYP,TZP); COSMO charge densities displayed as a diverging color map (red = positive values, blue = negative values) with maximum values set to 0.015 and -0.015, respectively.

TD-DFT calculations were performed at the same level of theory, applying the Davidson algorithm or the tight binding (+TB) approximation.[13, 14] A comparison of the obtained results is depicted below. As is to be expected for cyanine dyes, significant deviations between theoretical and experimental results were observed.[15] The only exceptions to this are the calculations performed applying the continuous solvation model in combination with the Davidson algorithm. In TD-DFT calculations with continuous solvation models, non-equilibrium solvation was used. Although typical non-equilibrium solvation is performed by

only using the optical part of the dielectric constant ($\varepsilon_{NEQL} = \eta^2$, with η = refractive index of the respective solvent), here results closer to measured values could be obtained using a value of ε = 5. These results may suggest that it is necessary to use more sophisticated solvent models. Preliminary scanning of excitation energy of **1-Me**, as calculated by TDDFT, against ε_{NEQL} revealed an asymptotic relationship, with the linear HOMO → LUMO transition converging against a value close to the experimental values. As shown by Pascal *et al*, electron densities in this position are lower for the keto-polymethine than for the *meso*-Cl dye, indicating a shift from the cyanine limit towards a bis-dipole type structure.[16]

Figure S53 shows the structures of **1-Me** and **2-Me** colored by Voronoi deformation density (VDD) charges. Mulliken charges show a similar trend (charge in the *meso*-position 0.030 for **1-Me** and 0.499 for **2-Me**)

Table S3 lists calculated vertical (HOMO-LUMO) transition energies applying various either TDDFT+TB (time-dependent DFT using tight-binding methodology) or TDDFT using the Davidson algorithm.

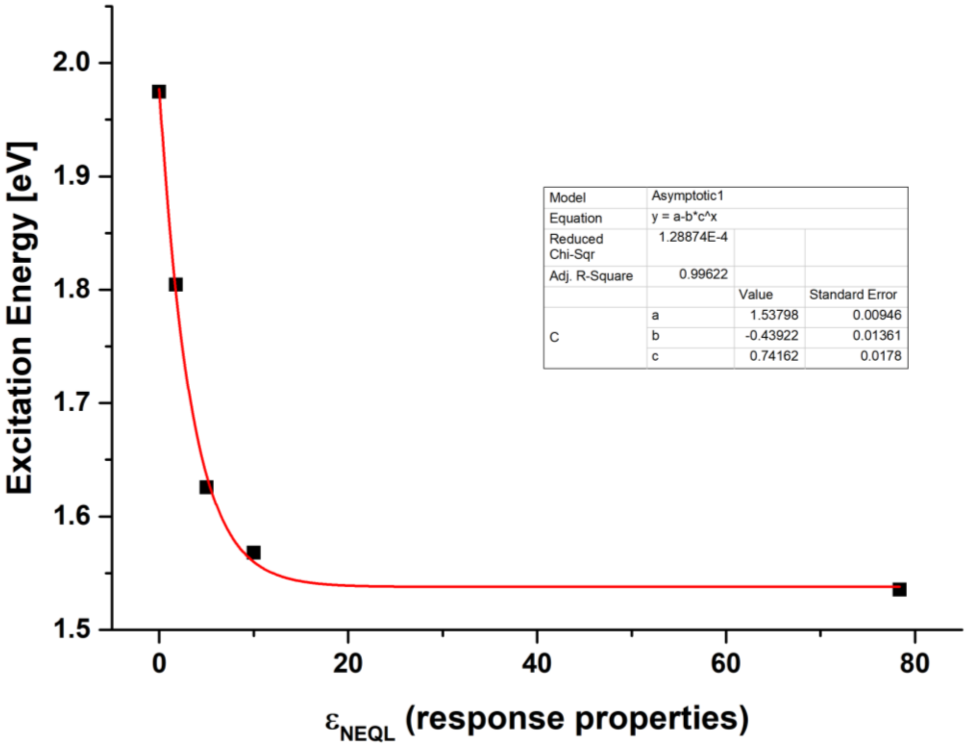

Figure S54. Plot of computed excitation energies of **1-Me** (TDDFT, BLYP/TZP) against various non-equilibrium dielectrics chosen for the calculation. From left to right: ε_{NEQL} = 0 (NOCSMRSP), 1.7689 (ε_{Opt}), 5, 10, 78.9 (ε_{aq}), depicted as black squares. Asymptotic fit indicated as a red line.

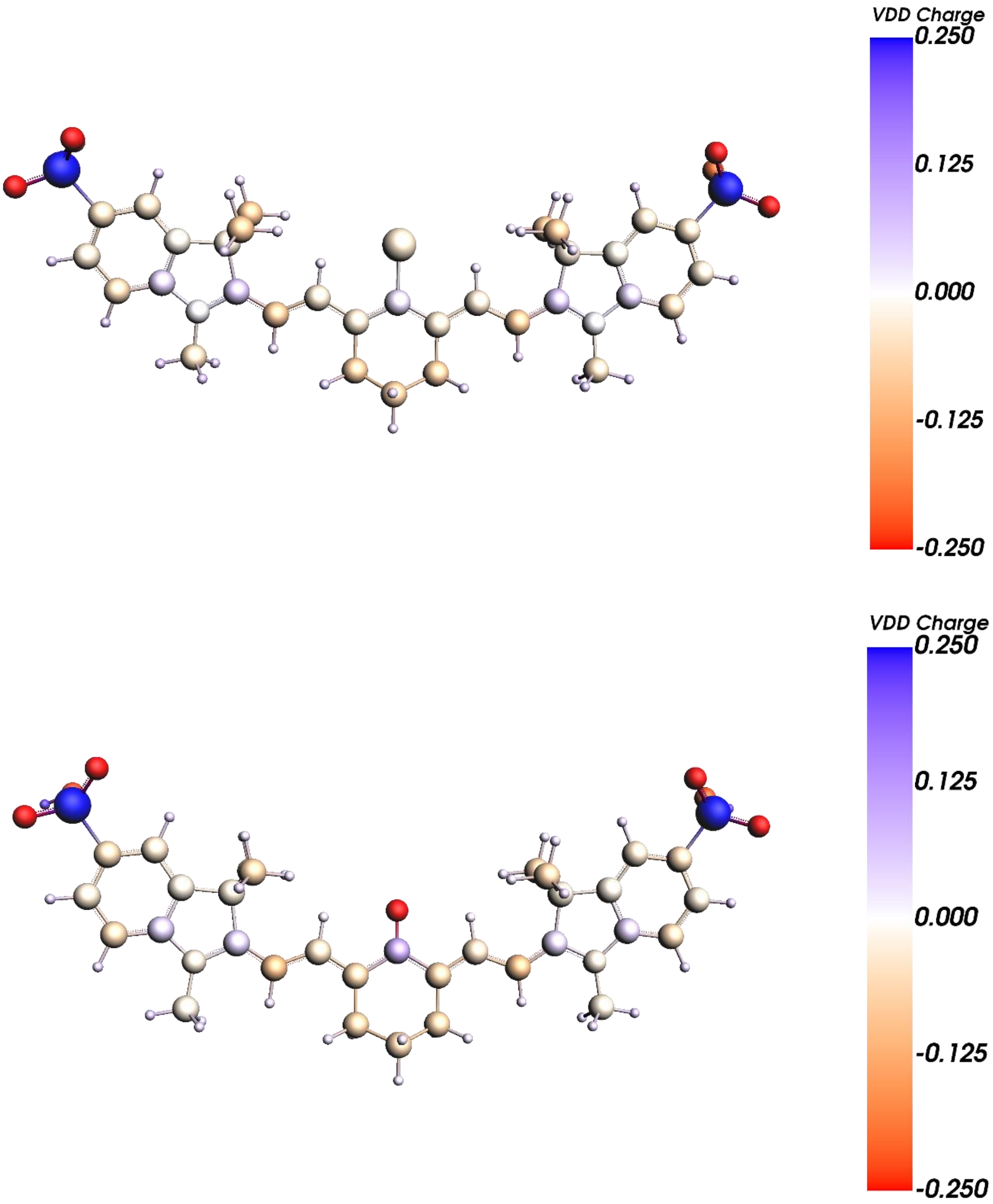

Figure S55. Depiction of **1-Me** and **2-Me** with atoms colored by Voronoi density deformation (VDD) charge (max. 0.25/min -0.25), indicating increased charge flow out of the *meso*-position of **2-Me** (BLYP/TZP, gas phase).

Table S3. HOMO-LUMO transitions (*169a* → *170a* for **1-Me**; *165a* → *166a* for **2-Me**, contribution of single orbital transitions in excess of 99 % in all cases) applying various methodologies (BLYP/TZP). [a] = in PBS. In calculations featuring COSMO-solvation a non-equilibrium solvation model (NEQL) was used for calculation of excitation energies with $\varepsilon_{NEQL} = 5$.

Compound	TDDFT+TB [nm/ev]	Davidson [nm/eV]	Experimental[a] [nm/eV]
**1** (Gas Phase)	616.3 / 2.0117	622.3 / 1.9922	-
**1** (COSMO; H_2O)	n.d.	762.7 / 1.6256	782 / 1.5855
**2** (Gas Phase)	550.6 / 2.2519	551.3 / 2.2490	-
**2** (COSMO; H_2O)	n.d.	598.7 / 2.0706	563 / 2.2022

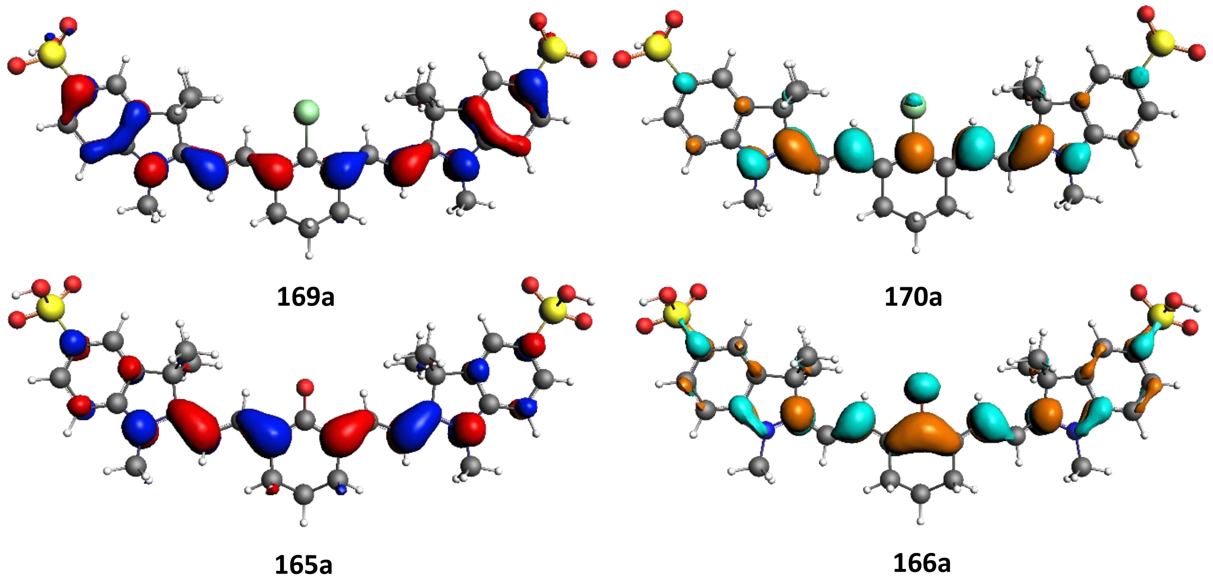

Figure S56. Depiction of HOMO/LUMO (red and blue / orange and cyan) of compounds **1-Me** and **2-Me** as isosurfaces at 0.03 au (BLYP/TZP, gas phase).

XYZ-Coordinates:

Model I-Me

1	C	0.000012	-0.000015	-0.000004
2	C	0.000001	-0.000006	0.000011
3	C	0.000007	0.000003	0.000013
4	C	-0.000014	0.000019	0.000012
5	C	0.000026	-0.000022	0.000007
6	C	0.000005	0.000010	-0.000001
7	C	0.000001	-0.000010	-0.000016
8	C	-0.000023	0.000022	-0.000002
9	C	0.000006	-0.000003	-0.000001
10	C	-0.000002	0.000037	-0.000004
11	C	0.000025	0.000027	-0.000004
12	N	-0.000010	-0.000001	0.000031
13	N	0.000005	-0.000004	0.000008
14	C	-0.000014	-0.000004	0.000001
15	C	-0.000004	-0.000018	0.000014
16	H	0.000010	-0.000006	0.000007
17	H	0.000004	-0.000004	-0.000001
18	H	-0.000001	-0.000012	0.000005
19	H	0.000002	0.000000	0.000005
20	H	0.000008	-0.000008	0.000000
21	H	-0.000003	-0.000001	0.000001
22	H	0.000002	0.000003	0.000001
23	H	-0.000010	-0.000066	0.000003
24	H	-0.000015	-0.000029	0.000000
25	H	0.000009	0.000010	-0.000003
26	H	-0.000001	-0.000004	0.000008
27	H	0.000006	-0.000005	-0.000001

28	C	-0.000013	0.000006	-0.000011
29	H	-0.000003	-0.000002	0.000004
30	H	-0.000001	-0.000003	0.000005
31	H	0.000009	-0.000003	0.000006
32	C	-0.000004	0.000035	-0.000012
33	H	0.000000	0.000000	-0.000011
34	C	0.000015	0.000088	-0.000010
35	C	-0.000012	-0.000033	-0.000027
36	C	-0.000006	0.000000	-0.000012
37	C	-0.000016	-0.000009	0.000014
38	H	-0.000018	0.000014	-0.000002
39	H	0.000001	0.000005	-0.000003
40	H	0.000004	0.000004	0.000001
41	C	0.000016	0.000021	0.000008
42	H	0.000002	0.000000	-0.000001
43	H	0.000000	-0.000002	-0.000001
44	H	0.000012	-0.000001	-0.000005
45	C	0.000013	0.000002	-0.000007
46	C	-0.000001	-0.000001	0.000010
47	C	0.000016	0.000019	0.000016
48	C	-0.000044	0.000006	-0.000001
49	H	0.000003	-0.000006	0.000004
50	H	-0.000006	-0.000002	-0.000005
51	H	-0.000002	-0.000003	-0.000002
52	Cl	-0.000011	-0.000011	0.000000
53	C	-0.000043	-0.000074	-0.000027
54	C	-0.000004	-0.000007	0.000018
55	C	0.000015	0.000038	0.000004
56	H	0.000003	-0.000001	0.000002
57	H	-0.000004	-0.000010	-0.000014
58	H	0.000002	-0.000003	0.000000

59 C   0.000000 0.000023 0.000000

60 H   -0.000006 0.000006 0.000003

61 H   -0.000003 0.000011 -0.000012

62 H   0.000001 0.000008 -0.000005

63 C   -0.000021 -0.000041 -0.000014

64 C   0.000003 -0.000008 -0.000003

65 C   -0.000012 0.000019 -0.000002

66 C   0.000015 0.000001 0.000007

67 H   0.000007 0.000007 0.000002

68 H   -0.000001 -0.000004 0.000000

69 H   -0.000002 0.000001 -0.000003

70 S   0.000031 -0.000023 -0.000008

71 O   0.000000 0.000007 0.000012

72 O   -0.000008 -0.000007 0.000003

73 O   0.000016 0.000032 -0.000002

74 S   0.000024 -0.000039 0.000004

75 O   0.000004 0.000046 -0.000006

76 O   0.000000 -0.000005 0.000001

77 O   0.000003 0.000001 -0.000014

78 H   0.000002 -0.000009 0.000002

79 H   -0.000006 -0.000006 0.000005

Model II-Me

1	C	-0.000003	0.000016	0.000007
2	C	-0.000029	-0.000033	-0.000023
3	C	0.000018	0.000017	-0.000002
4	C	-0.000016	-0.000025	0.000016
5	C	0.000011	0.000013	0.000012
6	C	-0.000011	-0.000009	-0.000003
7	C	0.000005	0.000018	-0.000002
8	C	-0.000003	-0.000012	-0.000020
9	C	-0.000006	0.000016	0.000001
10	C	0.000008	0.000001	-0.000013
11	C	-0.000001	0.000050	-0.000004
12	N	0.000020	-0.000003	-0.000005
13	N	-0.000005	-0.000012	0.000017
14	C	-0.000008	0.000014	-0.000002
15	C	0.000005	0.000004	-0.000008
16	H	0.000000	-0.000004	0.000005
17	H	0.000000	-0.000004	0.000003
18	H	0.000002	-0.000003	-0.000007
19	H	-0.000004	0.000002	-0.000001
20	H	0.000003	-0.000001	0.000003
21	H	0.000004	-0.000003	0.000000
22	H	0.000000	-0.000004	0.000001
23	H	0.000004	0.000024	0.000000
24	H	0.000004	0.000006	0.000002
25	H	-0.000001	-0.000004	0.000001
26	H	-0.000001	0.000003	-0.000004
27	H	0.000006	-0.000011	-0.000001
28	C	-0.000024	-0.000027	-0.000013
29	H	-0.000015	-0.000006	0.000011

30	H	0.000010	0.000031	-0.000006
31	H	0.000015	-0.000010	-0.000001
32	C	-0.000002	0.000000	-0.000001
33	H	0.000004	0.000005	0.000008
34	C	-0.000012	-0.000036	0.000002
35	C	-0.000017	-0.000051	-0.000001
36	C	-0.000008	-0.000015	0.000006
37	C	0.000004	0.000047	-0.000019
38	H	0.000003	-0.000007	-0.000003
39	H	-0.000001	-0.000004	-0.000002
40	H	0.000001	-0.000001	0.000001
41	C	0.000016	0.000022	0.000028
42	H	0.000001	-0.000002	0.000001
43	H	0.000001	0.000000	-0.000001
44	H	0.000007	0.000000	-0.000009
45	C	0.000016	0.000067	0.000005
46	C	-0.000009	-0.000003	0.000005
47	C	-0.000001	-0.000008	0.000002
48	C	0.000011	-0.000033	-0.000012
49	H	-0.000006	-0.000014	0.000006
50	H	0.000005	0.000002	0.000003
51	H	-0.000002	-0.000003	0.000001
52	O	0.000011	0.000010	0.000008
53	C	0.000026	0.000047	-0.000003
54	C	-0.000015	-0.000022	0.000007
55	C	-0.000015	-0.000019	-0.000004
56	H	0.000000	-0.000005	-0.000004
57	H	0.000000	0.000000	-0.000006
58	H	-0.000001	-0.000004	0.000003
59	C	-0.000001	-0.000010	0.000006
60	H	0.000000	0.000004	-0.000002

61 H 0.000002 0.000002 -0.000006

62 H 0.000000 -0.000003 0.000001

63 C -0.000014 -0.000024 0.000007

64 C -0.000010 0.000000 0.000011

65 C 0.000008 0.000036 -0.000008

66 C 0.000012 -0.000016 0.000004

67 H -0.000002 -0.000006 0.000003

68 H -0.000004 -0.000006 0.000000

69 H -0.000003 -0.000014 0.000003

70 S -0.000002 0.000035 -0.000003

71 O 0.000006 -0.000014 0.000005

72 O 0.000014 0.000019 -0.000009

73 O -0.000032 -0.000029 0.000007

74 S 0.000032 -0.000002 0.000001

75 O -0.000012 -0.000017 -0.000010

76 O 0.000008 0.000046 -0.000016

77 O -0.000005 -0.000009 -0.000001

78 H -0.000004 -0.000003 0.000012

79 H -0.000002 -0.000005 0.000007

References

[1] S. M. Usama, G. K. Park, S. Nomura, Y. Baek, H. S. Choi, K. Burgess, *Bioconjugate Chem.* **2020**, *31*, 248.

[2] S. Thavornpradit, S. M. Usama, G. K. Park, J. P. Shrestha, S. Nomura, Y. Baek, H. S. Choi, K. Burgess, *Theranostics* **2019**, *9*, 2856.

[3] D. H. Rich, J. Singh, in *Major Methods of Peptide Bond Formation, Vol. 1* (Eds.: E. Gross, J. Meienhofer), Academic Press, **1979**, 241.

[4] A. P. Gorka, R. R. Nani, M. J. Schnermann, *Org. Biomol. Chem.* **2015**, *13*, 7584.

[5] Y. Shi, X. Meng, H. Yang, L. Song, S. Liu, A. Xu, Z. Chen, W. Huang, Q. Zhao, *J. Mater. Chem. B* **2019**, *7*, 3569.

[6] A. H. Ponsford, T. A. Ryan, A. Raimondi, E. Cocucci, S. A. Wycislo, F. Fröhlich, L. E. Swan, M. Stagi, *Autophagy* **2021**, *17*, 1500.

[7] C.-M. Lin, S. M. Usama, K. Burgess, *Molecules* **2018**, *23*.

[8] G. te Velde, F. M. Bickelhaupt, E. J. Baerends, C. Fonseca Guerra, S. J. A. van Gisbergen, J. G. Snijders, T. Ziegler, *J. Comput. Chem.* **2001**, *22*, 931.

[9] E. Van Lenthe, E. J. Baerends, *J. Comput. Chem.* **2003**, *24*, 1142.

[10] A. Bérces, R. M. Dickson, L. Fan, H. Jacobsen, D. Swerhone, T. Ziegler, *Comput. Phys. Commun.* **1997**, *100*, 247.

[11] S. K. Wolff, *Int. J. Quantum Chem.* **2005**, *104*, 645.

[12] C. C. Pye, T. Ziegler, *Theor. Chem. Acc.* **1999**, *101*, 396.

[13] R. Rüger, E. van Lenthe, T. Heine, L. Visscher, *J. Chem. Phys.* **2016**, *144*, 184103.

[14] S. J. A. van Gisbergen, J. G. Snijders, E. J. Baerends, *Computer Physics Communications* **1999**, *118*, 119.

[15] B. Le Guennic, D. Jacquemin, *Acc. Chem. Res.* **2015**, *48*, 530.

[16] S. Pascal, A. Haefele, C. Monnereau, A. Charaf-Eddin, D. Jacquemin, B. Le Guennic, C. Andraud, O. Maury, *J. Phys. Chem. A* **2014**, *118*, 4038.

[17] U. Hennrich and M. Eder, *Pharmaceuticals*, 2021, **14**.

[18] A. W. Hensbergen, T. Buckle, D. M. van Willigen, M. Schottelius, M. M. Welling, F. A. van der Wijk, T. Maurer, H. G. van der Poel, G. van der Pluijm, W. M. van Weerden, H.-J. Wester and F. W. B. van Leeuwen, *J. Nucl. Med.*, 2020, **61**, 234.

[19] A. Iagaru, *J. Nucl. Med.*, 2017, **58**, 1883.

[20] K. P. Maresca, S. M. Hillier, F. J. Femia, D. Keith, C. Barone, J. L. Joyal, C. N. Zimmerman, A. P. Kozikowski, J. A. Barrett, W. C. Eckelman and J. W. Babich, *J. Med. Chem.*, 2009, **52**, 347.

[21] V.Ciaffaglione, P.A. Waghorn, R. M. Exner, F. Cortezon-Tamarit, S. P. Godfrey, S.Sarpaki, He. Quilter, R. Dondi, H. Ge, G. Kociok-Kohn, S. W. Botchway, I. M. Eggleston, J.R. Dilworth, and S. I. Pascu, *Bioconjugate Chem.* 2021, 32, 7, 1374.

HPLC Analysis Statement

Compound 1

Trace @ 780 nm

Purity (according to integration by Chromeleon software): >95 %

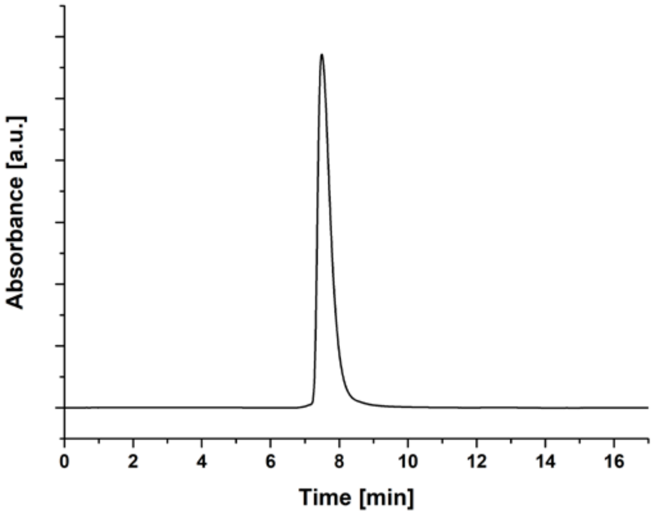

Compound 2

Trace @ 560 nm

Purity (according to integration by Chromeleon software): >95 %

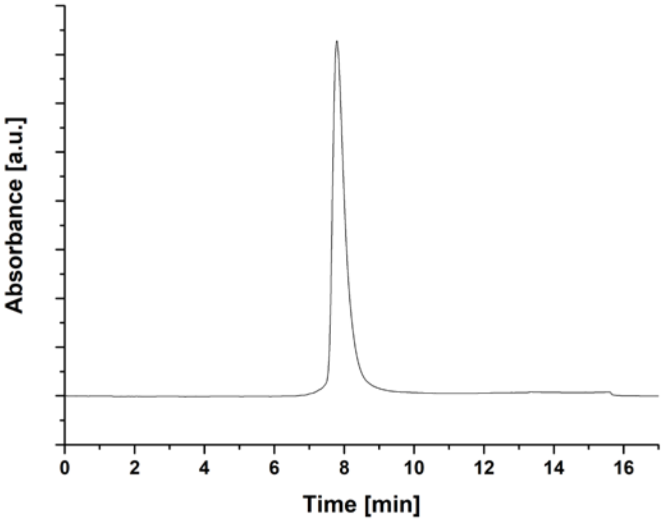

Compound 3

Trace @ 780 nm

Purity (according to integration by Chromeleon software): >95 %

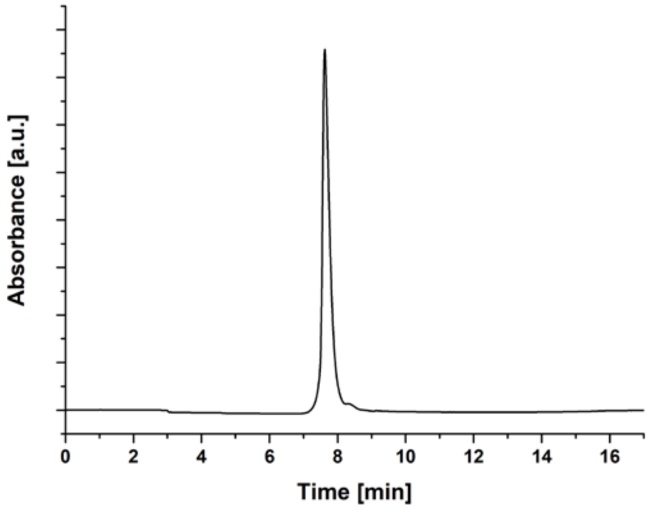

Compound 4

Trace @560 nm

Purity (according to integration by Chromeleon software): >95 %

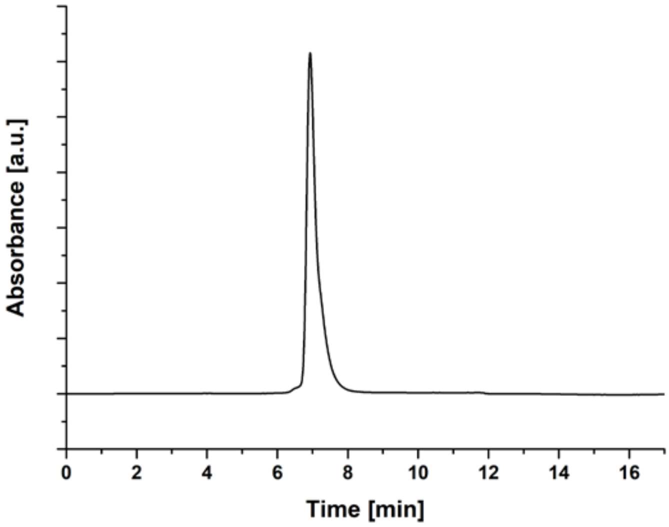

Compound 5

Trace @560 nm

Purity according to integration by Chromeleon software: >91 %; as discussed in Manuscript and extended Supplementary Information.

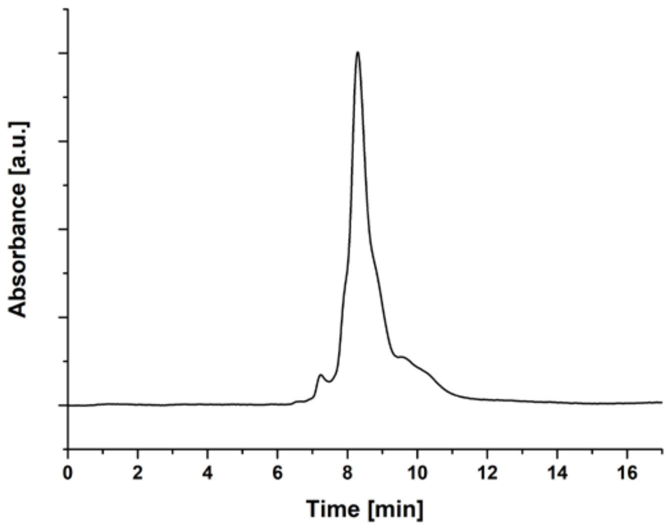

Supplement: Supplementary file 1 — bg2c00053_si_001.pdf [file bg2c00053_si_001.pdf]
